# Engineering the Defects and Microstructures in Ferroelectrics for Enhanced/Novel Properties: An Emerging Way to Cope with Energy Crisis and Environmental Pollution

**DOI:** 10.1002/advs.202105368

**Published:** 2022-03-03

**Authors:** Wen Dong, Hongyuan Xiao, Yanmin Jia, Long Chen, Huangfu Geng, Syed Ul Hasnain Bakhtiar, Qiuyun Fu, Yiping Guo

**Affiliations:** ^1^ State Key Laboratory of Metal Matrix Composites School of Materials Science and Engineering Shanghai Jiao Tong University Shanghai 200240 China; ^2^ Functional Ceramics of the Ministry of Education School of Optical and Electronic Information and Engineering Research Centre & Wuhan National Lab for Optoelectronics & Optical Valley Laboratory Huazhong University of Science and Technology Wuhan 430074 China; ^3^ School of Science Xi'an University of Posts & Telecommunications Xi'an 710121 China

**Keywords:** catalysis, defect engineering, energy harvesting, ferroelectric, microstructure engineering

## Abstract

In the past century, ferroelectrics are well known in electroceramics and microelectronics for their unique ferroelectric, piezoelectric, pyroelectric, and photovoltaic effects. Nowadays, the advances in understanding and tuning of these properties have greatly promoted a broader application potential especially in energy and environmental fields, by harvesting solar, mechanical, and heat energies. For example, high piezoelectricity and high pyroelectricity can be designed by defect or microstructure engineering for piezo‐ and pyro‐catalyst, respectively. Moreover, highly piezoelectric and broadband (UV–Vis–NIR) light‐responsive ferroelectrics can be designed via defect engineering, giving rise to a new concept of photoferroelectrics for efficient photocatalysis, piezocatalysis, pyrocatalysis, and related cocatalysis. This article first summarizes the recent developments in ferroelectrics in terms of piezoelectricity, pyroelectricity, and photovoltaic effects based on defect and microstructure engineering. Then, the potential applications in energy generation (i.e., photovoltaic effect, H_2_ generation, and self‐powered multisource energy harvesting and signal sensing) and environmental protection (i.e., photo‐piezo‐pyro‐ cocatalytic dye degradation and CO_2_ reduction) are reviewed. Finally, the outlook and challenges are discussed. This article not only covers an overview of the state‐of‐art advances of ferroelectrics, but also prospects their applications in coping with energy crisis and environmental pollution.

## Graphical abstract

The recent developments in defect/microstructure engineered ferroelectrics in terms of piezoelectricity, pyroelectricity, and photovoltaic effects are first summarized. Then, the applications in energy and environmental fields are reviewed. Finally, the outlook and challenges are discussed. This article not only covers an overview of the state‐of‐art advances of ferroelectrics, but also prospects their applications in coping with energy crisis and environmental pollution.

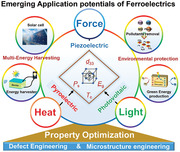

## 1 Introduction

**Figure 1:**
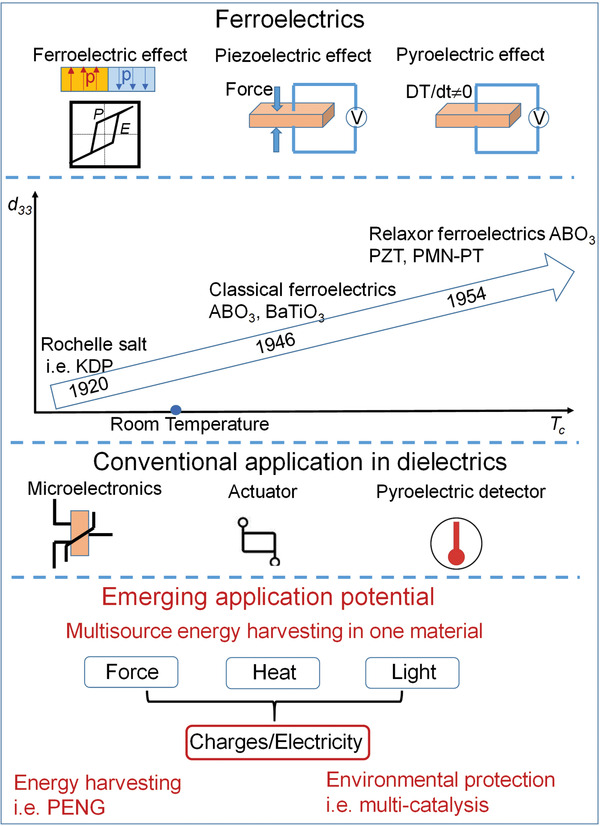
First part: Schematic illustration of ferroelectric, piezoelectric, and pyroelectric effects in ferroelectrics. Second part: Mapping of piezoelectric coefficient (*d*_33_) and *T*_c_ relative to some important time frame. Third part: Conventional application of ferroelectrics in dielectrics. Fourth part: Emerging application potential (i.e., energy harvesting and environmental protection).

Since the discovery of ferroelectricity in Rochelle salt by Valasek in 1920, ferroelectric, as an analog of ferromagnetic, has seen its development from fundamental properties to applications for a century.^[^ ^1^ ^]^ A ferroelectric is typically defined as a material with intrinsic polarization *P* that can be reversed under an external electric field *E*. Ferroelectrics usually have a phase‐transition temperature *T*_c_ above which they are paraelectric if they do not melt. As shown in the first part of **Figure** **1**, ferroelectric materials show very interesting properties such as ferroelectricity, piezoelectricity, and pyroelectricity owning to the nature of ionic displacement order in a noncentrosymmetric crystal.

As shown in the second part of Figure 1, the first discovered Rochelle salt ferroelectrics, such as potassium dihydrogen phosphate (KDP), shows relatively low ferroelectric phase transition temperature below ≈122 K due to their hydrogen bonding. The hydrogen bond was originally thought to be essential for ferroelectricity. This was changed from 1946 upon the discovery of robust perovskite oxide BaTiO_3_ with above room temperature ferroelectric transition and structural simplicity combined with theoretical work.^[^ ^2^, ^3^ ^]^ The high dielectric constant and stable ferroelectric properties above room temperature inspired their applications in electroceramic industry. Most ferroelectric families are not oxides, but the most studied ones are oxides because of their robustness and practical applications.

Before 1970, most researches focused on modeling ferroelectric phase transitions and exploring new ones. BaTiO_3_ shows high dielectric constant and has been widely used as capacitive materials in ceramic capacitors. PbZrO_3_–PbTiO_3_ (PZT), as one of the mainstay piezoelectrics, shows high piezoelectric constant (*d*_33_) on the order of 500 to 700 pC N^‐1^ and are widely used in microelectronics (i.e., ferroelectric‐field‐effect transistor) and piezoelectric actuators since its discovery in 1954.^[^ ^4^, ^5^ ^]^ Later on, the development of more complex PbTiO_3_‐based relaxor ferroelectrics, such as Pb(Mg_1/3_Nb_2/3_)O_3_–PbTiO_3_ (PMN–PT) realized a much higher *d*_33_ above 900 pC N^‐1^.^[^ ^6^, ^7^, ^8^ ^]^ These were very successful for actuators and piezoelectric transducers as well as for pyroelectric detectors as shown in the third part of Figure 1.^[^ ^9^ ^]^ Meanwhile, lead‐free ferroelectrics, such as titanate‐based system (i.e., NaBiTiO_3_‐BaTiO_3_)^[^ ^10^, ^11^, ^12^, ^13^, ^14^ ^]^ and alkali niobite‐based system (i.e., Na_0.5_K_0.5_NbO_3_–LiTaO_3_),^[^ ^2^, ^15^, ^16^, ^17^, ^18^, ^19^, ^20^ ^]^ have shown progresses in achieving high piezoelectricity in lead‐free ferroelectrics and their applications are promising.^[^ ^21^ ^]^ For example, the highest *d*_33_ can be ≈700 pC N^‐1^ in textured (K,Na)NbO_3_‐based lead‐free ceramics.^[^ ^16^ ^]^ Their applications can be exampled in sonar, bypass capacitor, and ferroelectric memories. Most ferroelectric devices were limited to ceramic forms due to the high cost of single crystals. Advanced methods and new mechanisms are developed in order to further enhance the ferroelectric properties and performance. For example, ultrahigh piezoelectricity in ferroelectric ceramics can be designed by judiciously introducing local structural heterogeneities to manipulate interfacial energies at morphotropic phase boundary (MPB) in relaxor ferroelectrics. The successful fabrication of Sm‐doped Pb(Mg_1/3_Nb_2/3_)O_3_–PbTiO_3_ (PMN–PT) relaxor achieved a much higher *d*_33_ for polycrystal ceramics (1200 to 2500 pC N^‐1^) and single crystal (3400 to 4100 pC N^‐1^), which is nearly an order of magnitude higher than that of PZT (500 to 700 pC N^‐1^).^[^ ^22^, ^23^, ^24^ ^]^ Ferroelectrics generally show high dielectric constant, which makes them promising for energy storage application due to their superior charge–discharge capabilities as well as its high temperature stability over electrochemical batteries.

These advancements do not suggest that the research in ferroelectric has stood still since then. Ferroelectrics remain a rich field of study. As shown in the bottom part of Figure 1, ferroelectric materials show emerging application potentials in clean energy harvesting and environmental protection. The main reason is that ferroelectrics can transfer solar, thermal, and vibration energies into charges/electricity, enabled by their photovoltaic, piezoelectric, and pyroelectric effects. The energy transfer can be exampled in piezoelectric nanogenerator (PENG) for energy harvesting^[^ ^25^, ^26^, ^27^, ^28^, ^29^, ^30^ ^]^ and catalysis for environmental protection.^[^ ^31^, ^32^, ^33^, ^34^ ^]^

**Figure 2:**
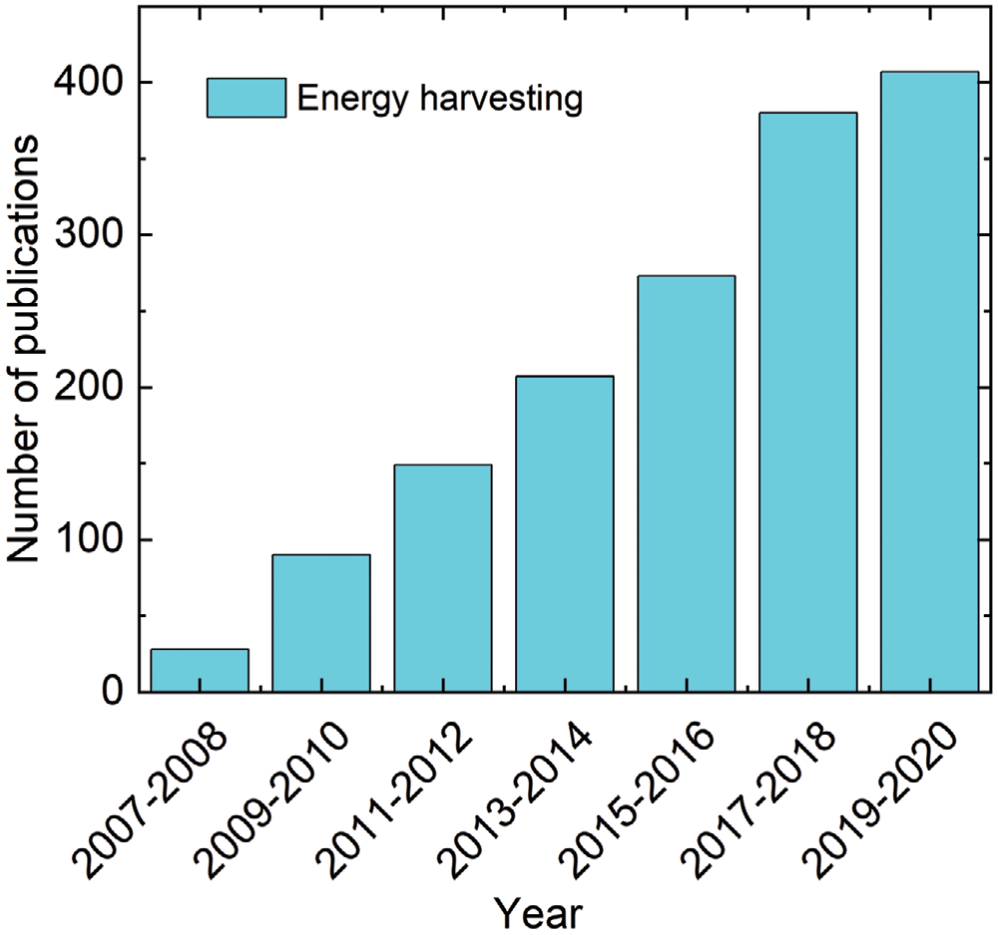
Number of publications on ferroelectric‐based energy harvesting from 2007 to 2020. Collected from Web of Science, search “ferroelectric/energy harvesting”.

By simultaneously harvesting illumination, vibration, and temperature fluctuation, ferroelectric‐based multisource energy harvester can also be achieved. By contrast, conventional energy harvesters, such as photovoltaic and thermoelectric, are mainly single‐source energy harvesters. In most cases, a variety of unstable or noncontinuous energy sources coexist. For example, kinetic/mechanical energy and thermal energy typically coexist in machines and human bodies. In outdoor environment, solar energy generally coexists with vibration/mechanical, and thermal energies. Therefore, ferroelectric‐based multisource energy harvesters would be advantageous over conventional single source ones to supply more stable energy.^[^ ^35^ ^]^ For example, as shown in **Figure** **2**, the research publications in ferroelectric‐based energy harvesting show a continuous increase in recent years. The merits of these multifunctional properties from ferroelectrics are expected to open an alternative way to cope with energy crises and environmental pollution especially when we are coming to the era of low‐carbon economy.

To clarify the issues on the emerging application of ferroelectrics in energy crisis and environmental protection, we summarized the state‐of‐art researches on defect/microstructure engineered ferroelectrics catered for energy harvesting, H_2_ generation, and catalysis. This review consists of six major sections. The second section talks about the potential application of ferroelectrics in energy and environmental pollution. The third section focuses on mechanism and research progress of defect and microstructure engineered ferroelectrics with novel/enhanced properties. The fourth section introduces research progresses about the synthesis and fabrication methods of ferroelectrics. The fifth section demonstrates the emerging energy and environmental applications in terms of coping with energy crisis and environmental pollution. The sixth section provides a summary about the emerging applications of ferroelectrics in environmental multisource energy‐driven generator and catalysis, pyro‐photoelectric catalytic H_2_ generation, and self‐powered multisource environmental signal sensing. Future challenges and chances are also prospected.

## 2 Potential Applications of Ferroelectrics in Energy and Environmental Pollution

Energy and environmental protection are becoming key global and societal challenges in our future. Semiconductor photocatalyst, such as TiO_2_, ZnO, ZrO_2_, ZnS, and CdS, have been investigated for over 50 years,^[^ ^36^ ^]^ but they generally suffer from low utilization of visible light. More importantly, their efficiency is limited by a high electron‐hole recombination rate, though which can be improved by forming heterogeneous structure with noble metals. In ferroelectrics, the charge separation due to spontaneous polarization‐induced electric field would be promising to improve the efficiency, making them potential candidates to make an impact in photocataysis.

**Figure 3:**
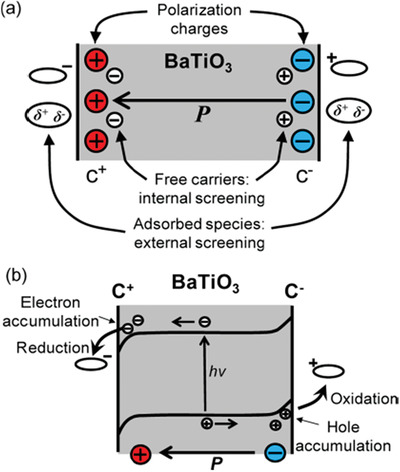
a) Schematic of a typical ferroelectric material showing internal polarization and screening mechanisms, b) the effect of free charge carrier reorganization on band structure and photoexcited carriers. Reproduced with permission.^[^ ^34^ ^]^ Copyright 2013, American Chemical Society.

Ferroelectric polarization leads to band bending and thus can efficiently reduce the recombination rate. Take ferroelectric BaTiO_3_ with a bandgap of 3.18 eV as a typical example, it has been reported to show organic dye degradation and water splitting.^[^ ^31^, ^32^, ^33^, ^34^ ^]^ As shown in **Figure** **3**, under light illumination, the spontaneous polarization induces macroscopic charges on the surface of ferroelectrics, which is compensated by free charge carriers and defects in the bulk and/or by the absorption of charged molecules from the environment. The sign of the charge is decided by the polarization direction.^[^ ^34^ ^]^ For example, the spontaneous polarization pointing from bulk to the surface will produce a positive charge on the surface, and vice versa. Moreover, it is suggested that the change of ferroelectric polarization direction can affect the absorption and desorption processes due to the different surface states, which gives the chance to overcome the limit of catalytic efficiency associated with Sabatier principle.^[^ ^37^, ^38^ ^]^ Other ferroelectric catalysts such as KNbO_3_,^[^ ^39^ ^]^ K_0.5_Na_0.5_NbO_3_,^[^ ^33^ ^]^ BiFeO_3_,^[^ ^40^, ^41^ ^]^ Bi_2_FeCrO_6_,^[^ ^42^ ^]^ and hybrid systems (i.e., TiO_2_/PbTiO_3_, and Ag/BiFeO_3_)^[^ ^43^, ^44^, ^45^, ^46^, ^47^, ^48^ ^]^ also show similar interesting photocatalytic behavior. More recently, new types of ferroelectrics, such as non‐oxide halide perovskite materials have also gained increasing attention in photochemical applications.^[^ ^49^ ^]^

Extensive researches have reported the combination of solar and mechanical vibration to promote photocatalytic behavior in BaTiO_3_ and Pb(Zr_0.52_Ti_0.48_)O_3_ (PZT), Bi_4_Ti_3_O_12_, MoS_2_, and nanocomposites, such as ZnO/TiO_2_ and MoS_2_/PDMS, etc.^[^ ^50^, ^51^, ^52^, ^53^ ^]^

**Figure 4:**
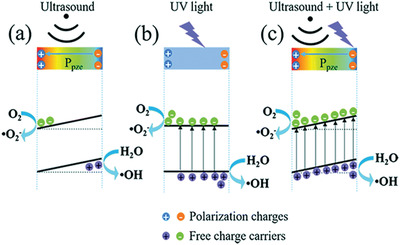
Schematic diagram of the catalytic mechanism under a) ultrasonic vibration only, b) UV irradiation only, and c) simultaneous ultrasonic vibration + UV light. Reproduced with permission.^[^ ^59^ ^]^ Copyright 2019, Royal Chemistry Society.

Actually, ferroelectrics show substantial piezocatalytic behavior in degradation of organic dyes and H_2_ generation under ultrasonic vibration,^[^ ^37^, ^54^, ^55^, ^56^, ^57^ ^]^ and even in tumor therapy.^[^ ^58^ ^]^ As shown in **Figure** **4**a, an impact force is exerted on the suspended ferroelectric Bi_2_WO_6_ particles via hydropressure caused by acoustic cavitation, and thus polarization charges are generated on the surface due to the piezoelectric effect.^[^ ^59^ ^]^ The free electrons and holes are attracted toward the opposite directions in the catalyst, then the electrons can react with the dissolved oxygen to yield superoxide radicals while holes can react with water to form hydroxyl radicals. Free electrons and holes can also be excited by UV light as shown in Figure 4b. The catalytic performance can be further enhanced by harvesting UV light and mechanical energy (Figure 4c).

**Figure 5:**
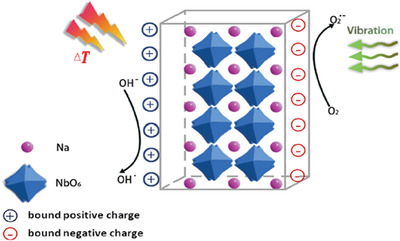
Schematic diagram for piezo‐pyro‐cocatalytic mechanism of NaNbO_3_ nanofiber. Reproduced with permission.^[^ ^61^ ^]^ Copyright 2018, Elsevier.

Considering the undiscovered field of pyrocatalysis, increasing attention has been put on using pyroelectric effect as an additional way for energy harvesting by coupling with the other two. Xu et al.^[^ ^60^ ^]^ reported a pyrocatalytic H_2_ generation and even CO_2_ reduction by harvesting cold‐hot alternating energy near room temperature using Ba_0.7_Sr_0.3_TiO_3_ nanoparticles. As shown in **Figure** **5**, You et al.^[^ ^61^ ^]^ reported a mechano‐pyro‐bicatalytic decomposition based on NaNbO_3_ nanofibers. The bicatalytic decomposition ratio of NaNbO_3_ nanofibers can reach up to 86.5%, which is higher than that of the pyrocatalysis (≈63.3%) and the piezocatalysis (≈75.8%). These researches suggest a higher efficiency and validate the multi‐catalytic approach based on ferroelectrics by harvesting multisource energies.

**Figure 6:**
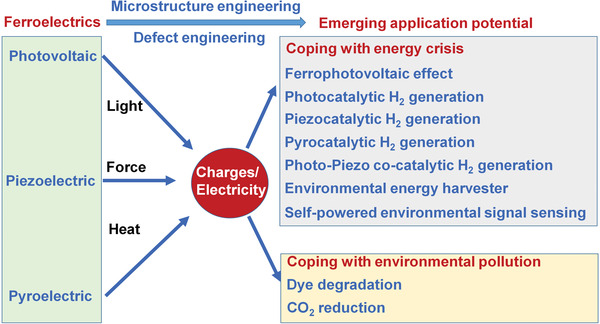
Outline of this review regarding the emerging application potential of ferroelectrics due to their photovoltaic, piezoelectric, and pyroelectric effect.

Therefore, as shown in **Figure** **6**, the photovoltaic, piezoelectric, and pyroelectric effects of ferroelectrics can transfer light, force, and heat into charges or electricity. Ferroelectrics enable multisource energy harvesting and show emerging application potential in coping with energy crisis and environmental pollution. For example, in this work, regarding energy crisis, we will review ferrophotovoltaic effect, photocatalytic H_2_ generation, piezocatalytic H_2_ generation, pyrocatalytic H_2_ generation, photo‐piezo cocatalytic H_2_ generation, environmental energy harvester, and self‐powered environmental signal sensing. For coping with environmental pollution, we will mainly talk about dye degradation and CO_2_ reduction

The multisource energy harvesting ability of ferroelectric is promising for multicatalytic applications. However, compared with commercial catalysts, there are still some obstacles that limit the efficiency of ferroelectrics. The main challenge is how to obtain ferroelectrics with simultaneously high‐performance piezoelectricity, pyroelectricity, and broadband light photovoltaic effects. First, ferroelectrics generally show the highest piezoelectricity at the MPB composition, but nanosize MPB ferroelectrics are hard to synthesize.^[^ ^62^, ^63^ ^]^ Second, ferroelectric materials are mainly UV photocatalyst due to their wide bandgaps over 3 eV. BiFeO_3_ shows especially lower bandgap (*E_g_*) of ≈2.7 eV among ferroelectric perovskite oxides,^[^ ^64^ ^]^ but it is still unable to absorb full visible spectrum and create large enough band edge position for H_2_ generation. Piezoelectric effect is suggested to be the main possible force to make the conduction band of BFO more negative than the H_2_/H_2_O redox potential (0 V) for H_2_ generation.^[^ ^65^ ^]^ Therefore, for semiconducting ferroelectric that we name photoferroelectric here, a broader absorption window is ideal for efficient photovoltaic and photocatalysis. Finally, pyrocatalysis generally requires high pyroelectric effect and chemical stability as well as high mobility at room temperature. However, most ferroelectrics have maximum pyroelectric effect around the Curie temperature (*T_C_*) the maximum polarization can be easily obtained.^[^ ^66^, ^67^, ^68^ ^]^

## 3 Defect and Microstructure Engineering

**Figure 7:**
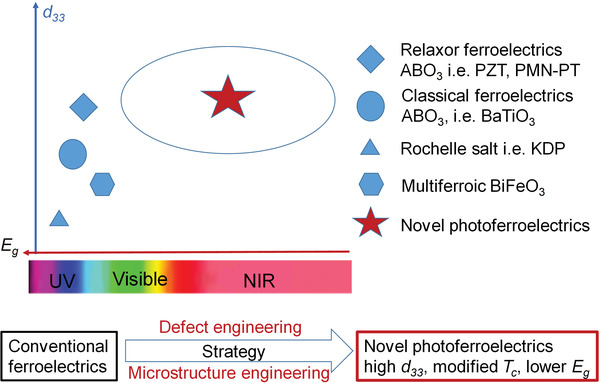
The strategy to transfer conventional ferroelectrics to novel photoferroelectrics where piezoelectric constant (*d*_33_), *T*_c_, and *E*_g_ are modified.

Ferroelectrics have been experimentally discovered a century ago, which have inspired tremendous researches on their fundamental properties and applications. From classic ferroelectrics (i.e., BaTiO_3_ and PbTiO_3_) to relaxor ferroelectrics, such as PbZrO_3_‐PbTiO_3_, PbMg_1/3_Nb_2/3_O_3_‐PbTiO_3_ (PMN‐PT), ferroelectrics generally show higher piezoelectricity *d*_33_ at MPB composition.^[^ ^4^, ^5^ ^]^ As shown in **Figure** **7**, ferroelectrics have been applied in piezoelectric actuator, nonvolatile memory devices. However, the bulk properties of ferroelectrics have reached a bottleneck via average structural design. Therefore, new approaches beyond average structure such as defect engineering and microstructure engineering are considered.

### 3.1 Defect Engineering

Point‐defect engineering in ferroelectrics has been a favorable way to break the bottleneck between structure and properties. Introducing dopants into host materials is a common approach to modify the properties.^[^ ^69^, ^70^, ^71^, ^72^ ^]^ Doping cannot only enhance the piezoelectricity, but also tune the *T*_C_, and the latter case also corresponds to an enhanced piezocatalytic activity.^[^ ^73^ ^]^

Piezoelectrics with *T*_C_ near room temperature have provided additional chances to enhance piezocatalysis. *T*_C_ is the point where the transition from ferroelectric to paraelectric state takes place, which is vital for electrical performance since the largest polarization change together with maximum piezo/pyroelectric properties are often found neat *T*_c_.^[^ ^73^, ^74^ ^]^ For example, the donor doping of Nb^5+^ on the B‐site (Ti^4+^ and/or Zr^4+^) in PZT can induce an enhanced piezoelectricity. The oxygen vacancies and “soften” effect of Nb in the PZT lead to lower *T*_c_ or coercive field *E*_c_.^[^ ^75^, ^76^, ^77^, ^78^ ^]^

The strategy for choosing the dopants is often based on the similarities between the dopants and host ions in terms of the size and electronegativity.^[^ ^79^ ^]^ Recently, novel doping strategies have been put forward, such as ionic codoping. This can be exampled in acceptor–donor codoped metal oxides (i.e., TiO_2_) where high‐performance colossal permittivity enabled by electron‐pinned defect‐dipoles can be obtained.^[^ ^71^, ^80^, ^81^, ^82^, ^83^ ^]^ Such electron‐compensated codoping strategy can also significantly improve the ferroelectricity, piezoelectricity, and temperature stability in ferroelectric perovskite oxides.^[^ ^84^, ^85^ ^]^ The codoping strategy overcomes the main challenge to introduce “difficult‐to‐dope” dopants into crystal structures at high concentrations especially through wet chemical synthesis.^[^ ^71^, ^86^, ^87^ ^]^ The “difficult‐to‐dope” dopants or sterically‐mismatched dopants can introduce structural frustrations, and thus provide a powerful chance to tremendously alter the chemical environment. The associated local structures surrounding the dopant/s can significantly change the properties of host materials and even create new functionalities.^[^ ^71^, ^80^, ^81^, ^82^, ^83^, ^86^, ^87^ ^]^

**Figure 8:**
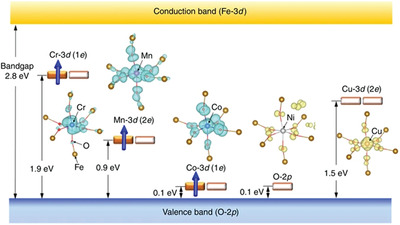
Schematic illustration of the bandgap engineered BiFeO_3_ doped with different transition metal ions according to DFT calculations. Reproduced with permission.^[^ ^89^ ^]^ Copyright 2017, Springer Nature.

**Figure 9:**
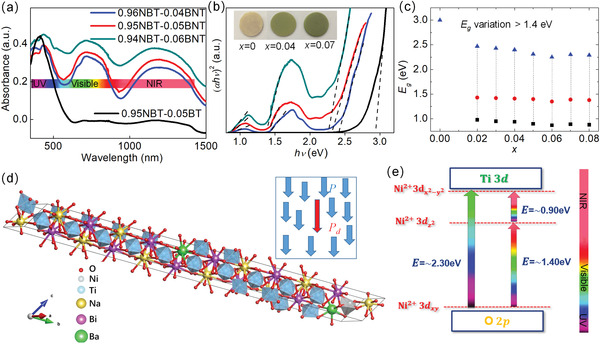
a) Photoabsorption, b) bandgap determination based on Tauc's relations,^[^ ^88^ ^]^ (c) calculated sub‐bandgap values of the defect‐engineered (1‐*x*)NBT‐*x*BNT (*x* = 0.04–0.06) with a 200 um thickness. d) 0.95NBT‐0.05BNT crystal structure used in DFT calculations. Inset image shows the schematic of the heterogeneous polar states of a ferroelectric where the arrow indicates the polar direction (*P* stands for host polarization, and *P*_d_ stands for defect‐induced polarization). e) Illustrated bandgap tuning mechanism with introduction of Ni^2+^, Ni 3d energy state plays a role as a scaffold to provide electron to Ti 3d (CBM) and receive the electron from O 2p (VBM). Reproduced with permission.^[^ ^90^ ^]^ Copyright 2018, Wiley.

Increasing the piezoelectricity in ferroelectrics is not enough to make full use of their properties in energy harvesting. An efficient light absorption is promising to enhance their photoelectric effect. By using defect engineering, the bandgap of the ferroelectrics such as BiFeO_3_, can be tunable by transition metal ion doping according to density functional theory (DFT) calculations (**Figure** **8**).^[^ ^89^ ^]^ However, the point‐defect doped ferroelectrics generally become leaky and loss room temperature ferroelectricity. In 2018, inspired by point‐defect‐mediated large piezoelectricity in ferroelectrics especially at the MPB region, we proposed an efficient strategy by judiciously introducing gap‐states at the MPB with a concrete example of Ni mediated (1‐*x*)Na_0.5_Bi_0.5_TiO_3_‐*x*Ba(Ti_0.5_Ni_0.5_)O_3_ (*x* = 0.02–0.08) (NBT‐BNT). The NBT‐BNT at the MPB region shows the best ferroelectricity/piezoelectricity (*d*_33_ = 151 pC N^‐1^, *P*_r_ = 31.2 µC cm^‐1^) among the doped NBT‐BNT compositions. As shown in **Figure** **9**a, the doped ceramics show an impressively wide UV–Vis–NIR absorption window due to the dopant‐induced gap‐states where the lowest one can be ≈0.9 eV (Figure 9b,c).^[^ ^90^ ^]^ Similar results have also been reported in similar relaxor ferroelectrics at MPB compositions. The reason for the reserved ferroelectricity is mainly attributed to the high interfacial energy at MPB. The high interfacial energy at MPB can drive the coupling between the local polar defect heterogeneities and ferroelectric polarization, leading to defect‐induced gap‐states whilst maintaining high piezoelectricity (Figure 9d).^[^ ^35^, ^91^, ^92^, ^93^, ^94^ ^]^ Sharma and Vaish^[^ ^95^ ^]^ reported a bandgap engineered Ba_0.85_Ca_0.15_(Ti_0.9_Zr_0.1_)_1‐_ *_x_* Fe*_x_* O_3_ ceramic with a low bandgap of 2.61 eV, and achieved piezo/pyro/photocatalytic activities, which validates the triple‐state multiple catalysts behavior of ferroelectric materials.

### 3.2 Microstructure Engineering

**Figure 10:**
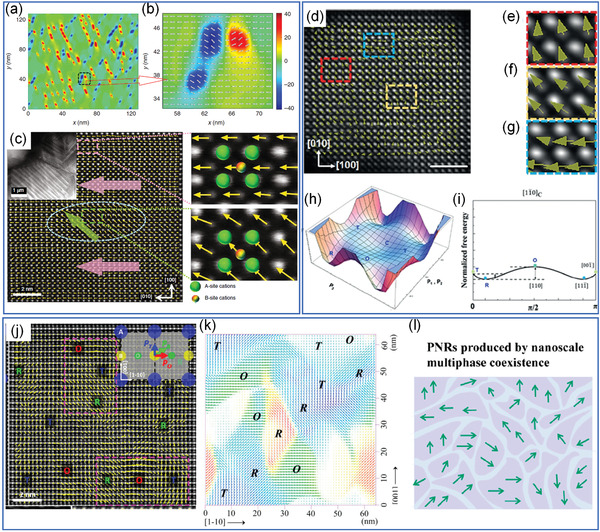
a) Microstructures for a [100]‐poled PNR‐ferroelectric composite at 100 K obtained by phase‐field‐simulation. b) Enlarged area of (a) with polar vectors. *x* and *y* axes represent the [100] and [010] crystallographic directions, respectively, the color bar denotes the angle between the polar vector of the grids and the [100] direction. Reproduced with permission.^[^ ^97^ ^]^ Copyright 2016, Springer Nature. c) STEM image recorded along [001] direction with polar vectors, the inset image was obtained in the dark‐field condition. The positions of the A‐site and B‐site atomic columns are indicated in the enlarged images on the right. Reproduced with permission.^[^ ^22^ ^]^ Copyright 2018, Springer Nature. d) Filtered STEM image of 10 nm BaTiO_3_ nanoparticles with polar vectors, showing multiphase. e–g) Enlarged images show the positions of A‐site and B‐site atomic columns. h) Schematic Landau free energy profiles for the simultaneous coexistence of three ferroelectric phases and its 2D projection as demonstrated in (i). Reproduced with permission.^[^ ^99^ ^]^ Copyright 2019, Wiley. j) Atomically resolved contrast‐reversed STEM ABF image along [110], with the displacement vector arrows are mapped; T, O, R regions are marked. k) 2D projection of the T‐O‐R three phase coexistence by phase field simulation on the (110) plane. l) Illustration of the polar nano regions with multiphase coexistence. Reproduced with permission.^[^ ^15^ ^]^ Copyright 2019, American Chemical Society.

MPB compositions generally possess optimum piezoelectric properties due to the weak energy barrier for polarization rotation seeded by chemical short‐range order, and local ferroic order.^[^ ^96^ ^]^ As shown in **Figure** **10**a,b, based on phase field simulation and dielectric analysis, Li et al.^[^ ^97^ ^]^ have reported that the contribution of local polar nanoregions in relaxors can facilitate the polarization rotation and high piezoelectric response. Later on, as shown in Figure 10c, by scanning transmission electron microscopy image (STEM) with atomic resolution, Li et al.^[^ ^22^, ^24^ ^]^ have further confirmed that the combination of different microstructures corresponds to different local nano polar regions (NPRs) coexist in the domain structure, forming a high interfacial energy. The stabilization of the NPRs can be achieved by introducing local structural heterogeneities to manipulate interfacial energies. The defect‐induced local heterogeneities will couple with the domain manipulated by the higher free energy at the MPB, leading to flattened free energy profiles. Upon this phenomenological principle, the ferroelectricity and piezoelectricity of MPB relaxors can be further enhanced by doping with sterically mismatched rare earth elements.^[^ ^23^, ^90^ ^]^ Similar microstructure engineering in ceramics can also be achieved by grain‐orientation engineered MPB compositions. For example, Li et al.^[^ ^98^ ^]^ developed a novel method to synthesize <111> textured Na_0.5_Bi_0.5_TiO_3_–Sr_0.7_Bi_0.2_TiO_3_ (NBT‐SBT) ceramics, leading to a reduced failure probability and improved Weibull breakdown strength. The above‐mentioned microstructure engineering will be promising to improve the energy harvesting of ferroelectrics and accelerate the development of more powerful ferroelectric‐based energy harvesters.

In addition to ferroelectric ceramics, microstructure engineering has also gained attention in nanoparticles for catalytic applications. Su et al.^[^ ^99^ ^]^ reported microstructure‐engineered BaTiO_3_ nanoparticles (NPs) with multiphase by hydrothermal synthesis without doping and orientation engineering. Figure 10d shows the STEM images of the nanoparticles, the polarization mapping suggests that there is coexistence of thee ferroelectric phases, including rhombohedral (R), tetragonal (T), and orthorhombic (R). BaTiO_3_ NPs show strong size‐related effects and thus give a more flexible change of local structure as well as an enhanced size‐dependent boundary effect. These effects lead to local structure deviations and coexistence of T, O, and R NPRs. As indicated in Figure 10h,i, the multiphase NPRs behave thermodynamically similar as the MPB relaxors with low energy profiles and facilitate the local polarization reorientation, leading to high piezoelectricity. For Alkali niobate‐based ferroelectrics, Wu et al.^[^ ^15^ ^]^ demonstrated that by microstructure engineering, emergent new phase boundary plays a key role in improving the piezoelectricity. The STEM image in Figure 10j shows coexistence of T, O, and R NPRs, which is further illustrated in Figure 10k,l. The rich phase boundary by microstructure engineering provides a guidance for the design of highly piezoelectric lead‐free ferroelectric ceramics. More related researches on microstructure‐engineered ferroelectric nanoparticles are highly expected in future to further improve catalytic performance.

## 4 Progress in Synthesis and Fabrication Method

The synthesis method of ferroelectrics is important to realize the defect and microstructure engineering. Solid‐state reaction (SSR) is a cost‐effective way to synthesize ferroelectrics at the MPB. However, to promote energy harvesting and catalytic performance, ferroelectrics generally need to be in the form of thin films or nanoparticles.

**Figure 11:**
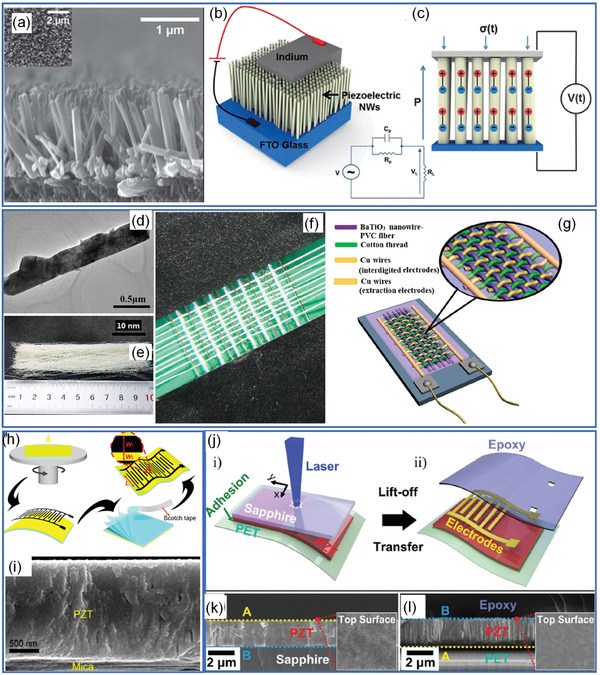
a) Cross‐sectional SEM image of BaTiO_3_ nanowire arrays (inset shows the top view) synthesized by two‐step hydrothermal method. b) Schematic diagram of the nano‐electromechanical system (NEMS). c) Schematic illustration of the energy harvesting (*P*, *V*, and *σ* indicates the polarization direction, piezo‐voltage, and stress). The inset at the bottom shows the electrical circuit representation of the piezoelectric energy harvester where the voltage (*V*_L_) measured with the load resistor (*R*_L_) indicates the AC power delivered to the load, *C*_p_ is the capacitance of the source and *R*_p_ is the leakage resistance. Reproduced with permission.^[^ ^101^ ^]^ Copyright 2014, Royal Society of Chemistry. d) TEM image of <001> oriented BaTiO_3_ nanowires. e) Optical image of a cluster of the BaTiO_3_ nanowire‐PVC composite microfibers. f) Optical image of the fiber nanogenerator. g) Schematic structure of the fiber nanogenerator. Reproduced with permission.^[^ ^103^ ^]^ Copyright 2015, Elsevier. h) Schematic illustration of the fabrication process of the flexible piezoelectric nanogenerator based on flexible mica substrates through wet‐chemical method through spin coating, annealing, electrode capping, mechanical exfoliation, and final device fabrication. i) A cross‐sectional image of PZT thin film coated on mica substrate. Reproduced with permission.^[^ ^109^ ^]^ Copyright 2018, Elsevier. j) The schematic illustration of the LLO process. k) Cross‐sectional image of the PZT film deposited on sapphire. l) Cross‐sectional image of the PZT film deposited on plastic substrate after LLO process. Reproduced with permission.^[^ ^110^ ^]^ Copyright 2014, Wiley.

For energy harvesting, ferroelectrics generally can be prepared into low dimensional nanowires or thin films by either wet‐chemical method or thin‐film deposition methods.^[^ ^100^ ^]^ For example, as shown in **Figure** **11**a–c, Koka et al.^[^ ^101^ ^]^ reported a vertically aligned BaTiO_3_ nanowire array on conducting substrates through a two‐step hydrothermal method. The BaTiO_3_ nanowire arrays were caped with top electrodes for piezoelectric energy harvesting.^[^ ^101^, ^102^ ^]^ Zhang et al.^[^ ^103^ ^]^ have reported a topochemical method to prepare BaTiO_3_ nanowires and then fabricated BaTiO_3_ nanowire‐PVC composite fiber fabrics to achieve flexible and wearable fabric piezoelectric nanogenerator (Figure 11d–g). This preparation method is quite similar as piezoelectric‐ZnO‐based nanogenerator for energy harvesting.^[^ ^100^, ^104^, ^105^, ^106^ ^]^ Thin‐film piezoelectric nanogenerator can be prepared on flexible substrates (i.e., flexible metal foil, and inorganic mica) through chemical solution deposition^[^ ^107^ ^]^ or sol‐gel method.^[^ ^108^, ^109^ ^]^ As shown in Figure 11h,i, Wang et al.^[^ ^109^ ^]^ have reported an all‐inorganic flexible piezoelectric energy harvester by a sol‐gel‐prepared PZT thin film on mica. By exfoliation of mica, flexible PZT thin films and flexible nanogenerator can be fabricated. Flexible ferroelectric thin films can also be prepared on inorganic substrates and then transferred to flexible substrate.^[^ ^110^, ^111^ ^]^ Park et al.^[^ ^110^ ^]^ proposed a method to fabricate large‐scale PZT thin films on flexible substrates by a laser lift‐off (LLO) process as shown in Figure 11j. The PZT thin film was first deposited on a double‐side polished sapphire substrate by a sol‐gel method combined with spin coating. The PZT thin film crystalized after annealing and then placed on a receiver plastic substrate coated with UV‐sensitive polyurethane as an adhesive. The PZT and the sapphire substrate can be separated with a beam. The laser beam with an energy of 4.03 eV is located between the bandgap energies of sapphire (10 eV) and PZT ceramics (3.2–3.6 eV), which can pass through the transparent sapphire and then vaporize the interface between the sapphire and PZT layer. This LLO‐assisted fabrication is promising to realize ferroelectric‐based flexible nano generator. Recently, even freestanding ferroelectric thin films can be obtained through conventional thin film deposition process combined with damage‐free lifting process, which paves the way to design novel substrate‐free flexible sensors.^[^ ^112^, ^113^ ^]^

For catalyst, in order to have high surface area, hydrothermal and sol‐gel methods are generally used to synthesize macro‐/nanosize ferroelectrics.^[^ ^65^, ^74^, ^99^, ^101^, ^114^, ^115^ ^]^ As mentioned in Section 3.2, the successful preparation of novel MPB BaTiO_3_ NPs will shed light on highly piezoelectric nano ferroelectrics for piezocatalysis and UV photocatalysis. Many more successful cases on synthesis of other ferroelectrics using hydrothermal method are expected. Considering that most ferroelectrics only show UV absorption, it would be favorable to further tune the bandgap in order to improve light absorption and the photocatalytic performance.

However, except from the successful synthesis of MPB BaTiO_3_ NPs and simple MPB PZT NPs, wet‐chemical methods are generally hard to synthesize more complex MPB relaxors. More importantly, aliovalent transition ions especially with larger size in combination with oxygen vacancies are not easy to enter the matrix to form desired defect states.^[^ ^116^ ^]^ SSR reaction has its unique advantages in synthesizing high piezoelectric ferroelectrics at MPB compositions whilst introducing gap‐states. However, conventional SSR can only synthesize gap‐state‐engineered relaxor ferroelectrics at MPB compositions in microscale size. We reported a SSR‐prepared lead‐free (K_0.48_Na_0.52_)NbO_3_‐(Bi_0.5_Na_0.5)_ZrO_3_ (KN‐BNZ) piezoelectric ceramics with specific cuboid shape in microscale and found an efficient oxidative desulfurization behavior.^[^ ^117^ ^]^ Compared with microscale KN‐BNZ particles, the research on the catalytic performance of the nanoscale particles is highly expected. Sharma and Vaish^[^ ^95^ ^]^ successfully prepared Fe doped Ba_0.85_Ca_0.15_(Ti_0.9_Zr0.1)_1‐_ *_x_* Fe*_x_* O_3_ and engineered the bandgap from 3.14 to 2.61 eV via SSR. Though Fe doping can induce substantial piezo/pyro/photocatalytic activities, the ceramic form has greatly limited the multicatalytic performance due to its small surface area. Meanwhile, the easy deformation of nanoscale ferroelectric materials can provide larger piezoelectric potential to enhance the catalytic performance.^[^ ^118^ ^]^ Therefore, a novel/improved method for both nanoscale and MPB relaxors is highly expected. For example, it is urgent to find a suitable method to synthesize the defect‐engineered nanoscale photoferroelectrics to realize and optimize their properties.

**Figure 12:**
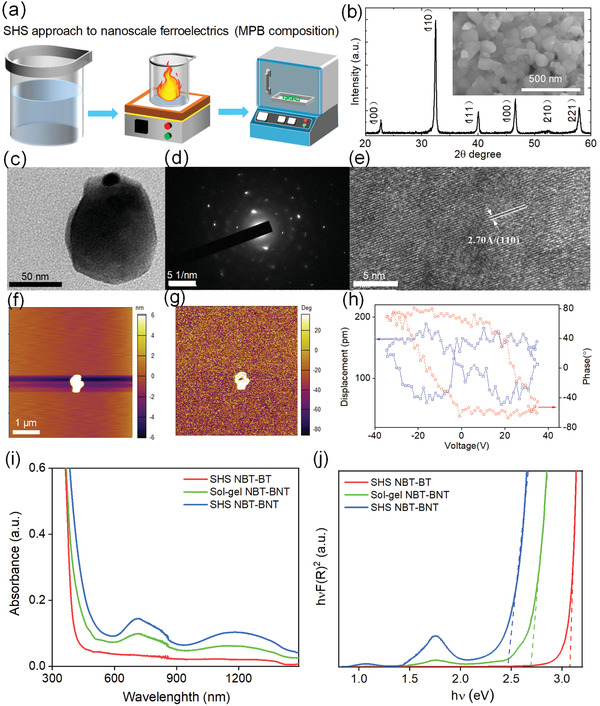
a) Illustration of the SHS process. b) XRD pattern of the NBT‐BNT NPs. The inset shows the SEM image. c) TEM bright‐field image, d) electron diffraction pattern, e) high resolution TEM image. f) Morphology image, g) phase image, and h) hysteresis loop measured by PFM. i) Photoabsorption spectrum of SHS‐prepared Ni doped NBT‐BNT (SHS BNT‐NBT), undoped NBT‐BT (SHS NBT‐BT), hydrothermal‐prepared NBT‐BNT NPs, and the sol‐gel NBT‐BNT NPs measured in the range of 300–1500 nm. The latter two samples were measured for a comparison. j) Bandgap determination of SHS NBT‐BNT NPs based on absorption data and Tauc's relations. Reproduced with permission.^[^ ^119^ ^]^ Copyright 2021, Elsevier.

In order to facilitate the doping of transition element, new synthesis method should consider both nanoscale synthesis and diffusion doping process. Recently, we reported a cost‐effective self‐propagating high‐temperature synthesis (SHS) to obtain Ni doped (Na_0.5_Bi_0.5_)TiO_3_‐Ba(Ti_0.5_Ni_0.5_)O_3_ nanoparticles (NBT‐BNT NPs).^[^ ^119^ ^]^ The SHS method can enable high‐temperature to promote the diffusion of Ni in the matrix while obtain nanoscale.^[^ ^120^ ^]^ **Figure** **12**a schematically shows the SHS method. The precursor solution first goes through a combusting process under high temperature that is large enough to drive the propagating of Ni into the MPB composition. In the meantime, a short combustion time and post‐annealing process facilitate the nanoscale synthesis and crystallization. Figure 12b shows pure phase with optimized size distribution and homogeneous doping of Ni in the NBT‐BNT NPs, which is further confirmed by TEM analysis (Figure 12c–e). Piezoforce microscopic (PFM) analysis confirmed the ferroelectric properties (Figure 12f–h). Moreover, As shown in Figure 12i,j, compared with sol‐gel or hydrothermal method, it is obvious that the SHS‐prepared NBT‐BNT NPs demonstrate much better UV–Vis–NIR absorption. Moreover, the high temperature provided by the SHS method drives the diffusion of the transition metal elements to form designed gap states. However, the obtained size distribution is poor compared with hydrothermal method. New synthesis method needs to be developed for defect‐engineered nanoscale photoferroelectrics.

## 5 Emerging Energy and Environmental Applications

Currently, the rapid development of modern society and industrialization has given rise to organic dye wastewater and energy crisis. Therefore, it is urgent to cope with energy crisis and environmental pollution.^[^ ^121^, ^122^ ^]^ Ferroelectrics provide a promising way to harvest energy by photovoltaic effect, or photocatalytic H_2_ generation, piezocatalytic H_2_ generation, pyrocatalytic H_2_ generation, and cocatalytic H_2_ generation. Ferroelectrics can also enable environmental multisource energy harvester and self‐powered environmental signal sensing. In addition to photocatalytic H_2_ generation, ferroelectric‐based catalytic dye degradation and CO_2_ reduction are hot research points in recent years.

### 5.1 Coping with Energy Crisis

#### 5.1.1 Ferrophotovoltaic Effect

**Figure 13:**
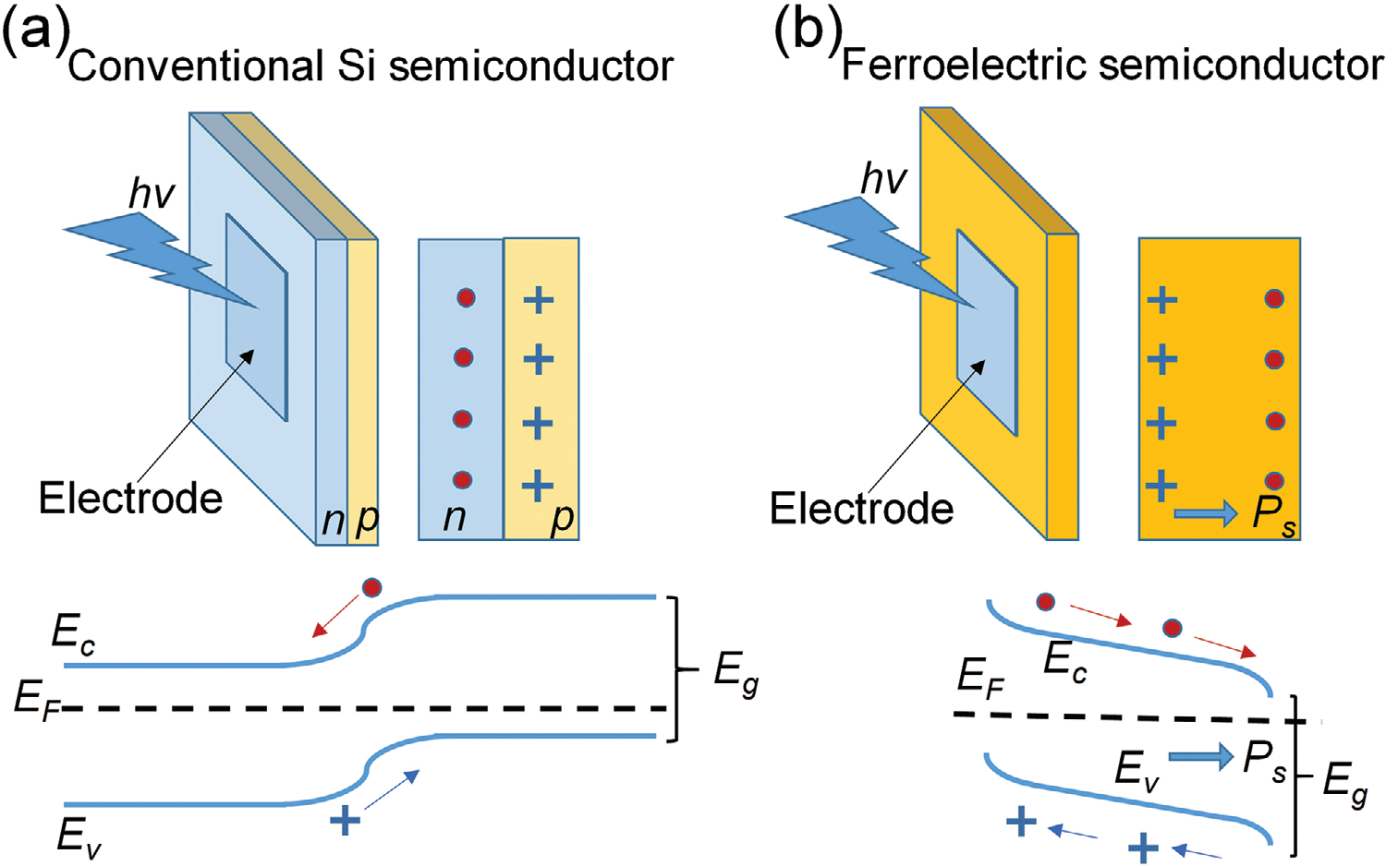
Schematic illustration of photovoltaic effect mechanism and bandgap diagram for a) conventional semiconductor and b) ferroelectric semiconductor.

**Figure 14:**
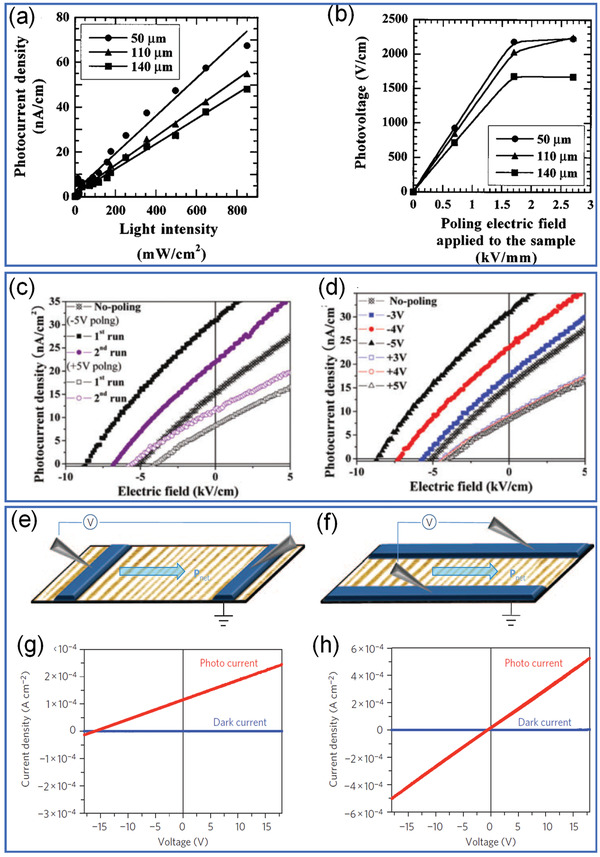
a) Light intensity dependent photocurrent density in 0.5 at% WO_3_ doped PLZT(3/52/48) bulk ceramics poled at 2 kV mm^‐1^ with different thicknesses (50 µm, 110 µm, and 140 µm). b) Poling electric field dependent photovoltage. Reproduced with permission.^[^ ^124^ ^]^ Copyright 1998, American Institute of Physics. The photocurrent density of KNN polycrystalline thin films c) under ±5 V poling states and d) various poling voltage. Reproduced with permission.^[^ ^126^ ^]^ Copyright 2005, American Institute of Physics. Schematic illustrations of the epitaxial BiFeO_3_ thin‐film based photovoltaic device geometries with e) polarization perpendicular and f) parallel to the measurement direction. Reproduce with permission.^[^ ^129^ ^]^ Copyright 2010, Springer Nature.

In early research, ferrophotovoltaic effect has been first demonstrated in (Pb,La)(Zr,Ti)O_3_ ceramics and then on thin films.^[^ ^123^, ^124^, ^125^ ^]^ As shown in **Figure** **13**a, in conventional semiconductors such as Si, their photovoltaic effects are mainly attributed to their low bandgaps. The photoexcited charge separation is mainly driven by the build‐in electric potential *V*_0_ related to p–n junction. The open‐circuit voltage *V*_oc_ is ideally equal to the *V*_0_. The *V*_oc_ is definitely lower than the *E*_g_/e where *E*_g_ is the bandgap. By contrast, in ferroelectrics, their photovoltaic effects are mainly attributed to the bandgap and separation of photoexcited charge carriers by spontaneous polarization (*P*_s_) (Figure 13b). Therefore, above bandgap *V*_oc_ can be generally obtained, giving rise to ferrophotovoltaic effect. However, the conventional semiconductor can generally show short‐circuit current (*J*_sc_) in the mA level, while the *J*_sc_ of conventional ferroelectrics is generally in nA level for ceramics and µA for thin films. One of the main reasons is due to their generally large bandgap (>3 eV). For example, as shown in **Figure** **14**, (Pb,La)(Zr,Ti)O_3_ (PLZT) (a,b) and (K_0.5_Na_0.5_)(Mn_0.005_Nb_0.995_)O_3_ (KNN) (c,d) thin films show few nA cm^‐2^ under one standard solar irradiation (AM1.5, 100 mW cm^‐2^).^[^ ^123^, ^124^, ^125^, ^126^ ^]^ In the PLZT, a linear relation between light intensity and photocurrent can be observed as that in conventional semiconductor. While the increase of photovoltage as a function of poling voltage indicates a unique effect of polarization on photovoltaic response. The photocurrent of polycrystalline thin film is generally larger than the ceramic form due to the much thinner thickness relative to the depletion region. Both the poling electric field and direction have effects on the photocurrent output. Then, investigation was focused on multiferroic BiFeO_3_ with lower bandgap of 2.5 to 2.7 eV.^[^ ^64^, ^127^, ^128^, ^129^, ^130^, ^131^ ^]^ The polarization‐dependent ferrophotovoltaic effect was nicely demonstrated in epitaxial BiFeO_3_ thin film devices where the photocurrent is only obvious when the polarization direction is parallel to the measurement direction as shown in Figure 14e,f. For the epitaxial BiFeO_3_ thin film, its *J*_sc_ can be as high as 0.12 mA cm^‐2^ with *V*_oc_ as large as 16 V under 285 mW cm^‐2^ irradiation as shown in Figure 14g,h. The polarization‐direction‐dependent photocurrent greatly inspired researches on ferrophotovoltaic effect.^[^ ^64^, ^127^, ^128^, ^129^, ^130^, ^131^ ^]^ Polycrystalline BiFeO_3_ thin films show *J*_sc_ in the level of 0.13 mA cm^‐2^ but with generally much lower *V*_oc_ below 1 V.^[^ ^64^, ^131^ ^]^ It is obvious that the much higher photocurrent of the BiFeO_3_ than that of the PLZT benefits from its much lower bandgap. However, the absorption window of BiFeO_3_ ceramics mainly locates at UV range. As we know, more than 80% of the sunlight corresponds to wavelength larger than 460 nm. Therefore, an intrinsic way to further increase the photocurrent is to enlarge the absorption window.

**Figure 15:**
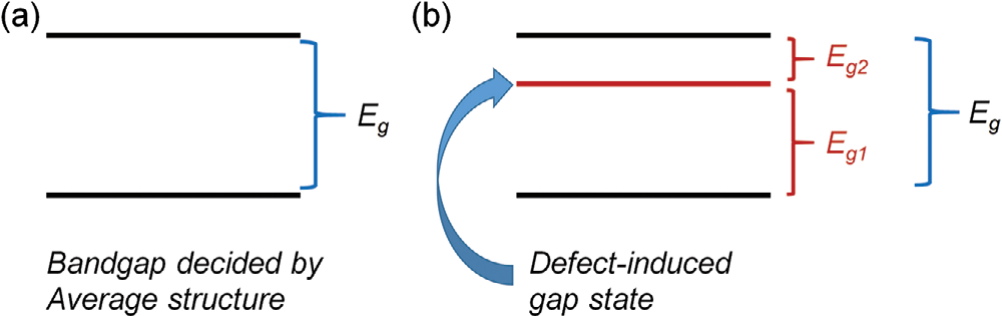
a) Illustration of bandgap (*E*_g_) decided by average structure, b) Illustration of the bandgap modified by defect‐induced gap‐state (*E*_g1_ and *E*_g2_).

To enlarge the absorption window, one needs to modify the bandgap of ferroelectrics. As shown in **Figure** **15**a, the bandgap (*E*_g_) of ferroelectrics generally corresponds to UV range. By doping transition elements into the ferroelectrics, gap‐states can be introduced to improve light absorption.

**Figure 16:**
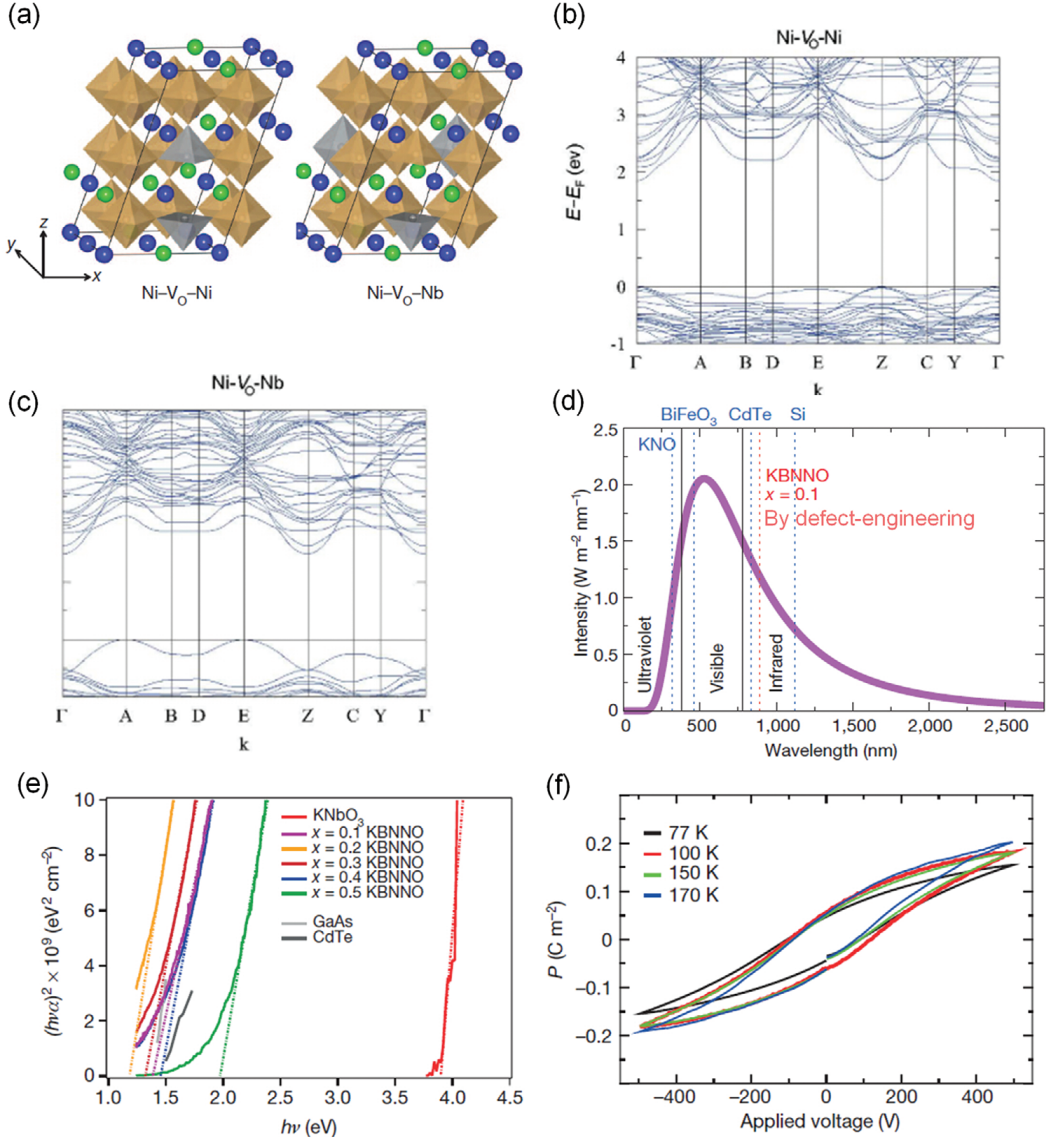
a) Defect structure model for calculation, K and Ba are shown by blue and green sphere, respectively, Nb–O_6_ and Ni–O_6_ are shown as brown and grey octahedra with O atoms at the vertices. b) The solar spectrum and *E*_g_ values for Si, CdTe, BiFeO_3_ and *x* = 0.1 KBNNO. c) Ellipsometry measurements for KBNNO oxides with *x* = 0.0–0.5, showing bandgaps of 1.18–3.8 eV. d) Ferroelectric hysteresis loops for a 20 µm thick *x* = 0.1 KBNNO film at 10^−7^ torr and 77–170 K. Reproduce with permission.^[^ ^70^ ^]^ Copyright 2013, Springer Nature.

In 2013, Grinberg et al.^[^ ^70^ ^]^ have done a pioneer work in designing visible light absorption ferroelectrics by defect engineering. A defect‐driven‐ferroelectricity and low bandgap state (even down to 1.1 eV) in perovskites (KNbO_3_)_1‐_ *_x_* (BaNi_1/2_Nb_1/2_O_3‐*δ*_)*_x_* (KN‐BNN) were demonstrated by doping two different transition‐metal cations (i.e., Ni, and Nb) at the B‐site in highly oxygen‐vacancy‐tolerable perovskite oxides (**Figure** **16**a). One cation (i.e., Nb) provides off‐center distortion and the other (i.e., Ni) decreases the different electronegativity within the perovskite B—O bonds to create electronic states in the gap (Figure 16b,c). The designed KN‐BNNO shows an impressively large absorption window extended to visible range that is comparable with the CdTe and far larger than conventional ferroelectrics such as BiFeO_3_ and KNO (Figure 16d,e). However, the KN‐BNN samples only show ferroelectricity at low temperature (77–170 K) (Figure 16f). Though KN‐BNNO loss room temperature ferroelectricity, the work inspired bandgap‐engineered ferroelectrics for efficient light harvesting.

**Figure 17:**
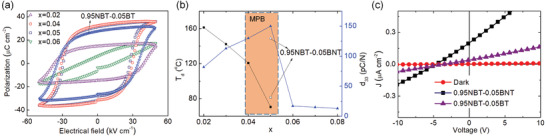
*P–E* loops of (1‐*x*)NBT‐*x*BNT (*x* = 0–0.6) ceramics measured at a frequency of 1Hz. b) The development of the depolarization temperature and piezoelectric coefficient (*d*_33_) as a function of the *x* in the composition (1‐*x*)NBT‐*x*BNT (*x* = 0.02–0.08), in which the values correspond to undoped 0.95NBT–0.05BT were provided as a comparison. Reproduced with permission.^[^ ^90^ ^]^ Copyright 2018, Wiley.

To save the room temperature ferroelectricity while introduce gap‐states in defect engineered ferroelectrics, we proposed an effective strategy. By doping transition metal cations in relaxor ferroelectrics, such as Ni‐doped 0.95Na_0.5_Bi_0.5_TiO_3_‐0.05BaT_0.5_Ni_0.5_O_3_, highly piezoelectric (*d*_33_ = 151 pC N^−1^, *P*_r_ = 31.2 µC cm^−2^) and UV–Vis–NIR light‐responsive relaxor ferroelectrics can be obtained (Figure 9).^[^ ^90^ ^]^ As shown in **Figure** **17**, both the ferroelectric polarization and piezoelectricity can be obviously improved by Ni doping. These results suggest that doping in the MPB composition will effectively save the room temperature ferroelectricity and improve the leaky behavior. The photocurrent density (≈200 nA cm^‐2^) increases approximately by two orders of magnitude compared with classic ferroelectric PLZT. Similar highly piezoelectric photoferroelectrics with UV–Vis–NIR light absorption were also reported in other relaxors at MPB compositions, such as (1‐*x*)(K_0.48_Na_0.52_)NbO_3‐_ *_x_* (Bi_0.5_Na_0.5_)(Zr_0.55_Ni_0.45_)O_3‐_ *_*δ*_* (KNN‐BNZN),^[^ ^132^ ^]^ PbTiO_3_‐Bi(Ni_2/3_Nb_1/3_)O_3_ (PT‐BNN),^[^ ^133^ ^]^ PbTiO_3_‐Bi(Ni_1/2_Ti_1/2_)O_3_.^[^ ^134^ ^]^ The photocurrent of the novel photoferroelectrics is an order of magnitude higher compared with PLZT and KNN ceramic or polycrystalline thin films. However, most of the researches are based on ceramics, the large thickness and grain boundary limit the photocurrent. Therefore, future research on the photovoltaic properties of the thin film is highly expected.

#### 5.1.2 Photocatalytic H_2_ Generation

**Figure 18:**
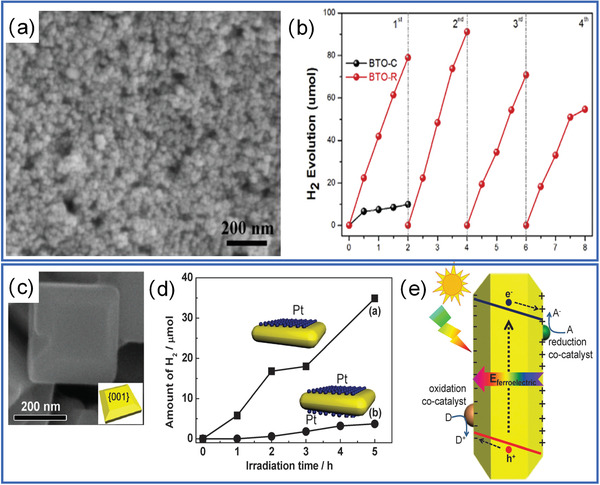
Morphology of a) BaTiO_3_ nanoparticles and b) their photocatalytic H_2_ generation. Reproduced with permission.^[^ ^136^ ^]^ Copyright 2018, Elsevier. c) Morphology of PbTiO_3_ nanoplates, d) H_2_ generation performance, and e) catalytic mechanism. Reproduced with permission.^[^ ^47^ ^]^ Copyright 2014, Royal Chemical Society.

Photocatalytic water splitting is promising for green H_2_ generation to cope with energy demands and environmental protection. Sunlight‐driven water splitting is sustainable and has been widely reported by using oxide semiconductors.^[^ ^135^ ^]^ Nowadays, ferroelectrics has attracted increasing attention in photocatalytic H_2_ generation/water splitting. Similar as conventional semiconductor, electrons in the valence band of ferroelectrics are first excited and then transformed to the conduction band, the inducing holes are left in the valence band.^[^ ^34^ ^]^ These electron‐hole pairs are separated by the internal electric field to the opposite sides of catalyst particles, which generate radicals for pollutant degradation and H_2_ production. As shown in **Figure** **18**a,b, by using BaTiO_3_ nanoparticles,^[^ ^136^ ^]^ substantial and reproducible H_2_ production can be obtained. The corresponding reaction processes can be summarized in the following Equations ((1), (2), (3)):

(1) $$\mathrm{BTO}\overset{hv}{\to }\left(h^{+}+e^{-}\right)$$

(2) $$h^{+}+H_{2}O\to 2H^{+}+\frac{1}{2}O_{2}$$

(3) $$2H^{+}+2e^{-}\to H_{2}$$

The photocurrent of ferroelectrics is restricted by the process of light adsorption.^[^ ^137^, ^138^, ^139^ ^]^ The electrons and holes move randomly in the solution, leading to a high recombination rate and low photocatalytic performance. Therefore, tremendous researches have focused on restricting the recombination rate.^[^ ^139^, ^140^, ^141^ ^]^ For example, by forming metal/ferroelectric junction, Pt/PbTiO_3_ nanoplate can generate a plasmonic effect to facilitate the charge separation, leading to an improved photocatalytic H_2_ generation (Figure 18c–e).^[^ ^47^ ^]^

**Figure 19:**
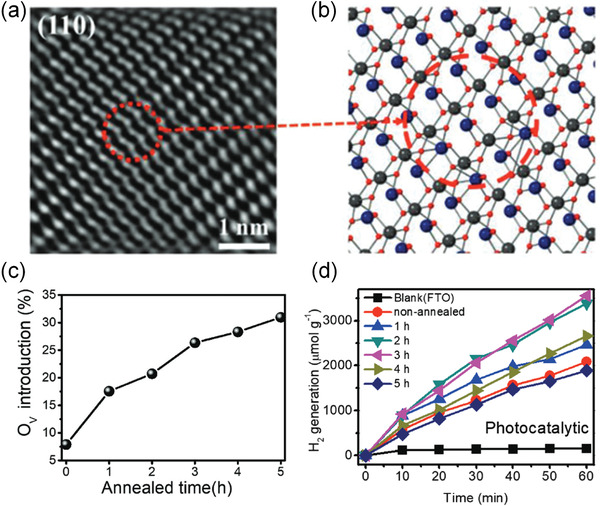
a) Magnified HRTEM image of ZnSnO_3_ nanowires for (110) plane. b) The corresponding schematic atom arrangement of the magnified area in (a). c) Percentage of oxygen vacancies introduced as a function of annealing time. d) H_2_ generation amount as a function of time for samples with different oxygen vacancy concentration. Reproduced with permission.^[^ ^142^ ^]^ Copyright 2020, Wiley.

In addition to the formation of metal/ferroelectric junction, doping has been considered to improve the photocatalytic performance. For example, Wang and Wu.^[^ ^142^ ^]^ reported the effect of controlled oxygen vacancy concentration on H_2_ generation in ZnSnO_3_ nanowires (NWs). **Figure** **19**a shows the high‐resolution transmission electron microscopy (HRTEM) image, Figure 19b illustrates the structures with large number of oxygen vacancies after annealing the sample under H_2_. Figure 19c shows the oxygen vacancy concentration as a function of annealing time. Figure 19d shows a maximum H_2_ generation rate can be achieved with moderate oxygen vacancy concentration under light illumination. The results suggest that a moderate oxygen vacancy concentration significantly improves the photocatalytic performance by nearly two times compared with that before annealing.

**Figure 20:**
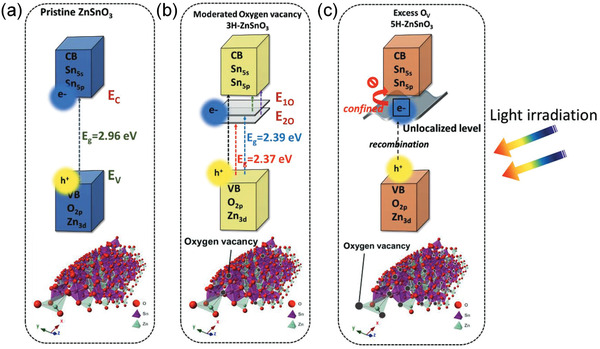
A schematic illustration of the simplified diagram of band structure for a) pristine ZnSnO_3_ NWs, b) 3H‐ZnSnO_3_ NWs (H means annealing hours) with moderated oxygen vacancies, and c) 5H‐ ZnSnO_3_ NWs with excess oxygen vacancies. Reproduced with permission.^[^ ^142^ ^]^ Copyright 2020, Wiley.

**Figure** **20** shows the mechanism for the enhanced photocatalysis after introducing oxygen vacancies. For the pristine sample, the photocatalytic efficiency is decided by the bandgap of 2.96 eV. While, for 3H‐ZnSnO_3_ with moderated oxygen vacancy concentration, oxygen vacancies serve as donor sites. The donor‐induced deep levels serve as donor bands (2.39 and 2.37 eV) and thus can enhance the lifetime of the photoexcited electrons. While, for excess oxygen vacancies, unlocalized levels will be generated and result in quenching traps where the decay rate and recombination rate would be high.

**Figure 21:**
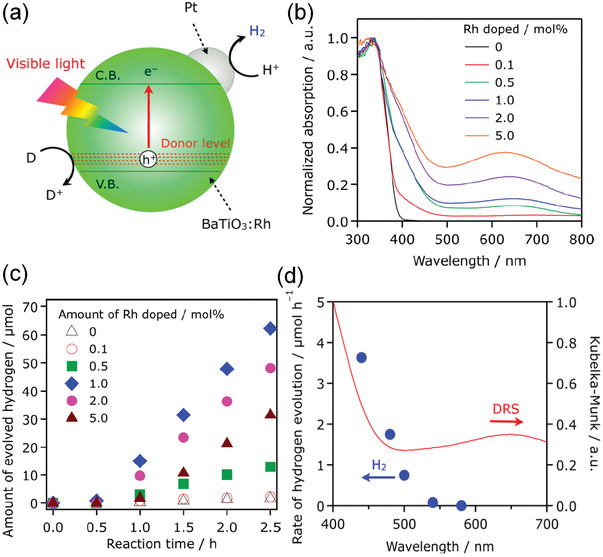
a) Schematic illustration of the photocatalytic H_2_ generation mechanism in Rh doped BaTiO_3_. b) Absorption spectra of BaTiO_3_ with different doping levels. c) Amount of H_2_ generation as a function of reaction time for samples with different doping levels. d) Dependence of the H_2_ generation rate from an aqueous methanol solution (10 vol%, 100 mL) using 25 mg Pt/BaTiO_3_:Rh (1.0 mol%) on the cutoff wavelength of incident light (300 W with a cold minor and a cutoff filter, Pyrex top‐irradiation type). Reproduced with permission.^[^ ^143^ ^]^ Copyright 2014, American Chemical Society.

Not only the oxygen vacancies can serve as donor bands to assist the separation of electrons, other dopant ions, such as transition metal or rare earth element, can also introduce gap states and enlarge the absorption window to visible range.^[^ ^143^, ^144^ ^]^ For example, as shown in **Figure** **21**a, doping Rh in BaTiO_3_ can provide a donor level.^[^ ^143^ ^]^ Similar as the oxygen vacancy doped ZnSnO_3_, an optimal light absorption (Figure 21b) and corresponding higher amount of H_2_ generation in the Rh doped BaTiO_3_ with a moderate doping level of 1.0 mol% (Figure 21c). Figure 21d indicates that the longest wavelength available for H_2_ generation was 540 nm, which coincides with the absorption threshold derived from Rh dopant.

The above examples suggest the important role of donor levels to enhance the light absorption. Therefore, both ion doping and heterojunction constructing are promising to enhance light absorption.^[^ ^145^, ^146^ ^]^ In this consideration, by synthesizing nanosize NBT‐BNT, dramatic enhancement of H_2_ generation performance can be obtained compared with that of the undoped NBT‐BT.^[^ ^119^ ^]^

#### 5.1.3 Piezocatalytic H_2_ Generation

Piezocatalysis provides a promising way to convert mechanical energy into chemical energy for renewable H_2_ generation or water splitting. Piezocatalysis involves the separation, transfer, and depletion of electrons and holes generated by piezoelectric effect. Compared with photocatalysis, the band tilting in piezocatalysis under force‐induced piezoelectric field will make the conduction band of ferroelectrics more negative than the H_2_/H_2_O redox potential for H_2_ generation.

**Figure 22:**
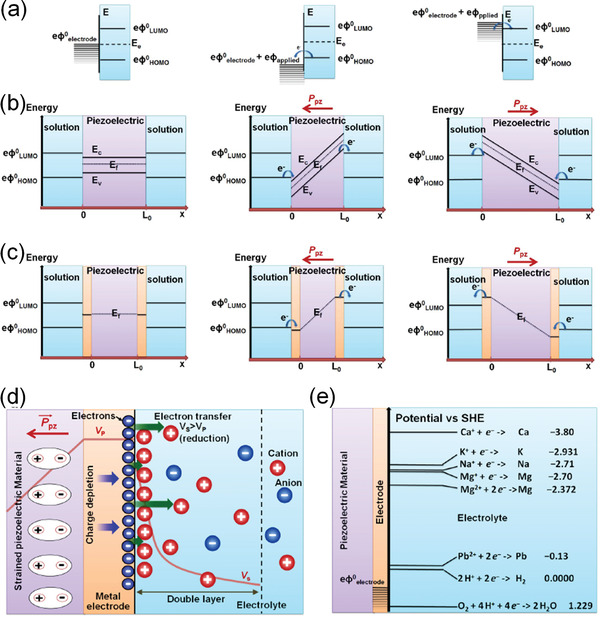
a) The change of the band structure from original (left) to lowered electronic energy levels compared with that of the HOMO (middle) and to higher occupied states compared with that of the LUMO (right). The change of the energy levels of b) conduction band (*E*_c_) and valence band (*E*_v_) and c) Fermi levels, corresponding to the cases in (a). d) Schematic illustrations of the charged surface that induces both capacitive double layer effects and electron transfer processes at the interface. e) Typical potential of the ions that can be thermodynamically reduced in the solution at high negative piezoelectric potentials by the electrode. Reproduced with permission.^[^ ^147^ ^]^ Copyright 2013, Springer Nature.

In order to comprehensively understand piezocatalysis, Starr and Wang^[^ ^147^ ^]^ proposed a general theoretical model by consideration of piezoelectric, semiconductor, molecular orbital, and electrochemistry. As shown in **Figure** **22**a, the band structure changes from original state (left) to either lowered electronic energy levels of unoccupied states compared with that of the highest occupied molecular orbital (HOMO) in solution (middle) or higher occupied states (right) compared with that of the lowest unoccupied molecular orbital (LUMO) in solution. For the middle case, the oxidation of the solution would happen when electrons leave the HOMO and transfer to unoccupied states within the electrode. In the right case, the solution will be reduced as the electrons transfer to the LUMO from the electrode. For nonmetal electrode, the *eφ*_HOMO_ and *eϕ*_LUMO_ logic will expand to the valence (*eφ*_VB_) and conduction (*eφ*_VB_) band edges, respectively. Piezocatalysis is driven by the piezoelectric‐induced polarization potential. For an ideal and simple piezoelectric material, the total energy shift (*V*_total_) by mechanical deformation is given by:

(4) $$V_{\mathrm{total}}=w_{x}T_{n}d_{xn}/\left(\varepsilon_{0}\varepsilon_{r,n}\right)$$

where *T*_n_ is an applied stress in the *n* dimension, *w_x_* is the width of the piezoelectric material in the *n* dimension, *d_xn_* is the piezoelectric modulus, *ε*_0_ and *ε_r,n_* are vacuum permittivity and relative permittivity in the *n* dimension, respectively. Assuming that the strain does not change the magnitude of the bandgap, the electrons will be energetically enabled to leave the VB and transfer to LUMO as shown in Figure 22b when the e*φ*_VB_ is close to *eϕ*_LUMO_. While the electrons will leave the HOMO and transfer to the CB. In this case, the electrode will provide continuous density of states in relation to their Fermi energies between the piezoelectric materials and solution corresponding to the cases in Figure 22b,c. Due to the charge transfer to and from the piezoelectric or electrode surfaces, the system acts as a capacitor, and the change of the piezoelectric potential depends on both the properties of piezoelectric materials and their surrounding solution. As shown in Figure 22d, the application of electrical potential from an external force drives the electron transfer reactions. The charged reactant ions are capacitively coupled to the electrode surface, which can effectively reduce the surface potential before electrochemical reduction or oxidization. Figure 22e shows the typical potential of the ions that can be thermodynamically reduced in the solution at high negative piezoelectric potentials by the electrode. Therefore, the piezocatalytic performance is related to the ions that are involved in the reaction.

**Figure 23:**
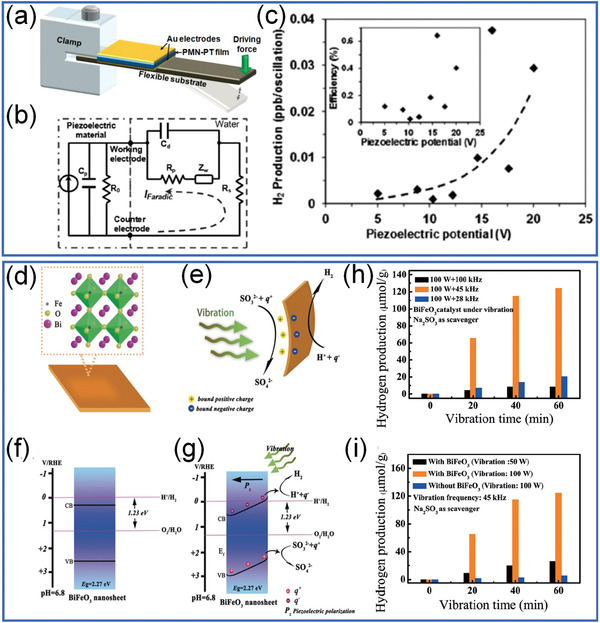
a) The schematic of the experimental setup for piezocatalysis by using a piezoelectric cantilever. b) The proposed equivalent circuit based on both piezoelectric circuitry element and a charge‐transfer/double‐layer analog for an electrochemical system. c) H_2_ production per oscillation versus peak piezoelectric‐induced electric potential. Reproduced with permission.^[^ ^148^ ^]^ Copyright 2012, Wiley. Schematic diagram of d) the BiFeO_3_ nanosheet and the piezoelectric‐induced surface charges under mechanical vibration and e) the piezocatalytic H_2_ generation reaction. f) Energy band diagram without mechanical vibration. g) Tilted energy bands under strong piezoelectric‐induced electric field and related redox reactions under mechanical vibration. The piezocatalytic H_2_ generation amount under ultrasound vibration via h) BiFeO_3_ nanosheets and at i) different vibration frequencies with different mechanical powers. Reproduced with permission.^[^ ^65^ ^]^ Copyright 2019, Wiley.

Early report of piezocatalysis can be found in bulk ceramic, as shown in **Figure** **23**, Starr et al.^[^ ^148^ ^]^ reported piezocatalytic reactions by using Au‐coated highly piezoelectric Pb(Mg_1/3_Nb_2/3_)O_3_‐32PbTiO_3_ (PMN‐PT) single crystal with a computer‐controlled actuator to induce strain. Figure 23a,b shows the experimental setup based on a piezoelectric cantilever and a proposed electric circuit. From Figure 23c, it is obvious that both the H_2_ generation rate and efficiency improve with the increase of piezoelectric potential. Later on, the advances in triggering ultrasound vibration in a sonicator make piezocatalysis easier to be applied. As shown in Figure 23d–i, You et al.^[^ ^65^ ^]^ have reported a high H_2_ generation rate of ≈124.1 µmol g^−1^ h^−1^ under 100 W ultrasonic vibration by using BiFeO_3_ nanosheets. The nanosheet shape facilitates the bending under vibration and increases the built‐in‐electric field. The negative electric charges (*q* ^−^) generated on the surface of BiFeO_3_ nanosheets will effectively react with the H^+^ in the water to produce H_2_. At the same time, the positive charges (*q* ^+^) are transferred to the opposite side of BiFeO_3_ nanosheets and then react with the sacrificial agents of SO_3_^2−^ as shown in Equations ((5), (6), (7)):

(5) $$\mathrm{BiFe}O_{3}\overset{\mathrm{vibration}}{\to }\mathrm{BiFe}O_{3}+q^{+}+q^{-}$$

(6) $$2H^{+}+2q^{-}\to H_{2}$$

(7) $$q^{+}+S{O_{3}}^{2-}\left(\mathrm{sacrificial}\;\mathrm{agents}\right)\to S{O_{4}}^{2-}$$

The substantial piezocatalytic performance of BiFeO_3_ has gained increasing interest for ferroelectric‐based catalysts. Meanwhile, another method is to form heterostructure that can facilitate the piezocatalysis. Inspired by the photocatalysis of Pd, Yang et al.^[^ ^149^ ^]^ deposited Pd on BiFeO_3_ nanosheets to facilitate the charge carrier separation and lowered the activation energy/overpotential through supplying highly active sites for the proton reduction reaction.

**Figure 24:**
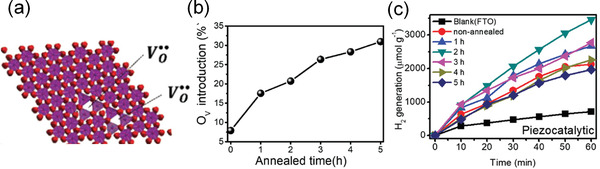
a) Illustration of the structure with oxygen vacancies. b) Oxygen vacancy concentration as a function of annealed time (*h*). c) H_2_ generation as a function of reaction time (min) for samples under different annealed time (*h*). Reproduced with permission.^[^ ^142^ ^]^ Copyright 2020, Wiley.

The piezocatalytic performance in BiFeO_3_ inspired the research in the field of piezocatalysis. To further enhance the piezocatalytic performance, one effective way is to engineer the bandgap by defect doping. For example, as we mentioned above, by doping moderate oxygen vacancy concentration in ferroelectric ZnSnO_3_, both the photocatalytic and piezocatalytic performance can also be improved as shown in **Figure** **24**.^[^ ^142^ ^]^ Figure 24a illustrates the structure with oxygen vacancies, by annealing the sample in H_2_ under different time, the oxygen vacancy concentration can be controlled. A moderate oxygen vacancy concentration under 1h annealing time would be optimal to obtain enhanced H_2_ generation (Figure 24c).

**Figure 25:**
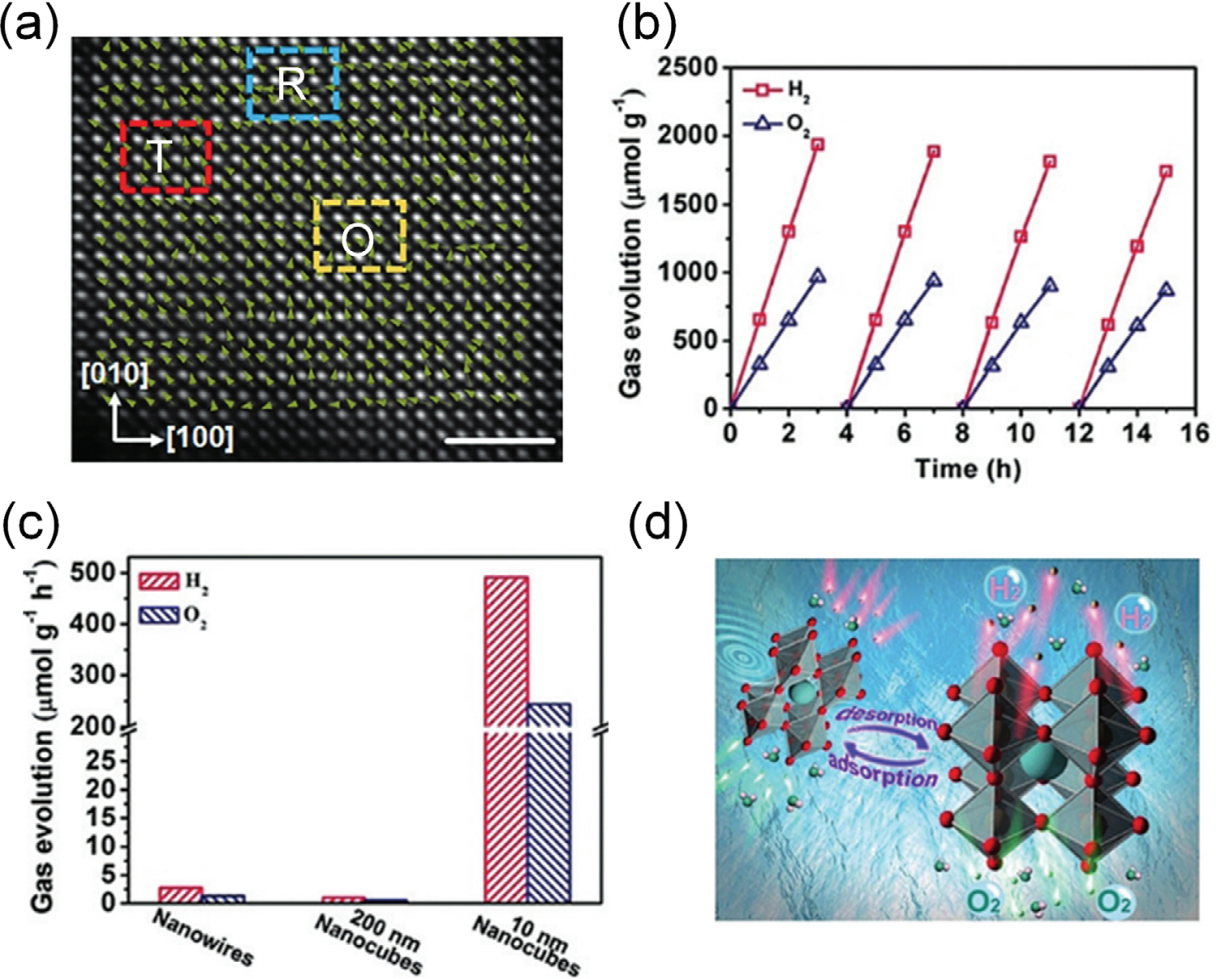
a) Filtered HAADF STEM image of 10 nm BaTiO_3_ nanoparticle showing Ba and Ti atomic positions with local spontaneous polarization direction (denoted by arrows) and multiphase coexistence (denoted by R, O, and T), scale bar, 2 nm. R: rhombohedra; O: Orthorhombic; T: Tetragonal. b) Typical time course of H_2_ and O_2_ generation of 10 nm nanoparticles under 60 kHz ultrasonic vibration. c) H_2_ and O_2_ generation rates on 10 nm BaTiO_3_ nanoparticles, 200 nm BaTiO_3_ nanoparticles, and BaTiO_3_ nanowires under 40 kHz ultrasonic vibration, respectively. d) Schematic illustration of the reaction mechanism by ultrasonic wave‐generated piezoelectric effect. Reproduced with permission.^[^ ^99^ ^]^ Copyright 2019, Wiley.

In addition to defect engineering, microstructure engineering is another effective method to enhance the piezocatalytic performance by improving the piezoelectricity, For example, Su et al.^[^ ^99^ ^]^ synthesize BaTiO_3_ nanoparticles with R, O, and T multiple phases as shown in the filtered HAADF STEM images (**Figure** **25**a). The multiple phase BaTiO_3_ nanoparticles with enhanced piezoelectricity demonstrate a high H_2_ production rate of 655 µmol g^−1^ h^−1^, which highlights the real potential of microstructure‐engineered piezoelectric catalysis for H_2_ production.

#### 5.1.4 Pyrocatalytic H_2_ Generation

Pyrocatalytic H_2_ generation is another application potential of ferroelectrics in addition to their photocatalytic and piezocatalytic H_2_ generation. Pyrocatalysis arises from the pyroelectric effect that creates an electric potential under the condition of temperature variation. Temperature variation is a common but rarely used energy source in our daily life. For pyrocatalysis, a sufficiently high electric potential difference for water splitting is a prerequisite. The electric potential is directly related to the charges on the surface and is intrinsically related to the pyroelectric coefficient *P*_pryo_. *P*_pryo_ is defined as the change of the spontaneous polarization vector *P* with temperature *T* as *P*_pyro_ = d*P*/d*T*.^[^ ^150^ ^]^ So *P* is not a constant but varies with the temperature.

**Figure 26:**
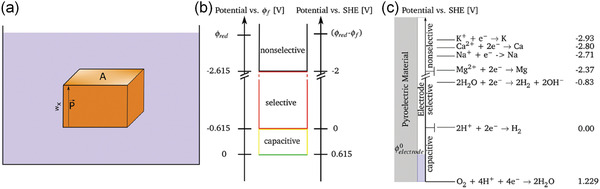
Schematic illustration of the model system for a) a pyroelectric material surrounded by a medium, b) reduction potentials of selected chemicals, and c) resting surface potential. Reproduced with permission.^[^ ^153^ ^]^ Copyright 2019, Royal Chemical Society.

Early researches on pyrocatalysis mainly focus on theoretical analysis. Xie et al.^[^ ^151^ ^]^ have investigated a range of pyroelectric materials and geometries for water electrolysis, and calculated the minimum thickness to generate a critical potential for water decomposition. Kakekhani et al.^[^ ^37^, ^56^, ^152^ ^]^ pointed out that the switchable surface polarization can be used in catalysis. In combination with the fields of pyroelectricity, electrochemistry, diffusion, and semiconductor theory, Schlechtweg et al.^[^ ^153^ ^]^ proposed a comprehensive and fundamental model to describe the thermally excited pyroelectric behavior in pure water. **Figure** **26**a shows the model system composed of a cuboid shape pyroelectric material in deionized water. The pyroelectric material has a thickness *w_x_* and two opposing surfaces (*A*) perpendicular to the polarization vector. The surface has an electrochemical equilibrium contact with the surrounding medium, and the energetic level is attached to the Fermi level of the electrode. Spontaneous polarization induces polarization charges on *A* in relation with temperature variation, which is screened by the dissolved ions or OH^−^/H_3_O^+^.^[^ ^37^, ^154^ ^]^ The surface potential *ϕ*_pyro_ can be given below:^[^ ^153^ ^]^

(8) $$\phi_{\mathrm{pyro}}=w_{x}\Delta TP_{\mathrm{pyro}}/\left(\varepsilon_{0}\varepsilon_{r}\right)$$

where Δ*T* is the temperature variation, *P*_pyro_ is the pyroelectric coefficient, *ε*_0_ and *ε*_r_ are vacuum and relative permittivity, respectively. Under symmetric polarization, the potential change on the reducing site is as below:

(9) $$\phi_{\mathrm{red}}=w_{x}\Delta TP_{\mathrm{pyro}}/\left(2\varepsilon_{0}\varepsilon_{r}\right)$$

The derivation of *ϕ*_red_ with respect to *t* is given by:

(10) $$\phi_{\mathrm{red}}^{\prime }\left(t\right)=w_{x}T^{\prime }\left(t\right)P_{\mathrm{pyro}}/\left(2\varepsilon_{0}\varepsilon_{r}\right)$$

where *T′(t)* is the temperature gradient at time *t*. The electric potential at the surface affects the electrochemical equilibrium state in contact with the medium. Figure 26b shows the *ϕ*_red_ values of some selected chemicals.^[^ ^155^ ^]^ In DI water, the resting surface potential is 0.615 V versus SHE, i.e., corresponds to the H_2_ and oxygen generation potentials. As a result, the surface potential is shifted by a pyroelectric‐induced electric potential from one side to negative value and to positive value on the other side. When the surface potential reaches above a critical level and the potential decreases below the highest HOMO of species in solution, oxidation process starts. While reduction reactions start if the surface potential becomes larger than the level of LUMO of the chemicals. Therefore, during the pyrocatalysis as shown in Figure 26c, the electric potential range changes according to the portion of faradic reactions. For example, in capacitive region, there is minor amount of faradaic processes. While, in the selective regime, partial faradaic process and capacitive process coexist. In nonselective reaction, an increasing amount of reaction sites are thermodynamically enabled due to the increased electric potential. Each chemical reaction has its potential range. Take DI water as an example, the reaction in the potential range of 0 to 1.23 V, in which 0.615 V on the reduction side and ‐0.615 V on the oxidizing side. Therefore, pyrocatalysis is a combination of thermodynamic and kinetic processes

**Figure 27:**
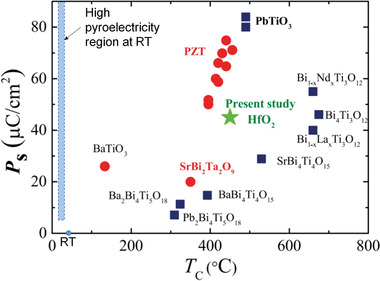
Mapping of *T*_C_ values of the normal perovskite oxide ferroelectrics. Ferroelectrics with high pyroelectricity for RT pyrocatalysis should ideally locate at the rectangular region. Reproduced with permission.^[^ ^156^ ^]^ Copyright 2016, Springer Nature.

Generally, in order to have large pyrocatalytic performance, ferroelectrics need to have *T*_c_ close to room temperature (RT) in order to have high pyroelectric coefficient. However, most ferroelectrics show *T*_c_ either much lower or much higher than RT, which limits their pyrocatalytic H_2_ generation performance. As shown in **Figure** **27**, most known perovskite oxide ferroelectrics show much higher *T*_C_ than RT, but ferroelectrics with high pyroelectricity should ideally locate in the rectangular region.^[^ ^156^ ^]^ Therefore, defect engineering and microstructure engineering as we described earlier, are emerging strategies to tune the *T*_C_ beyond average structure.

**Figure 28:**
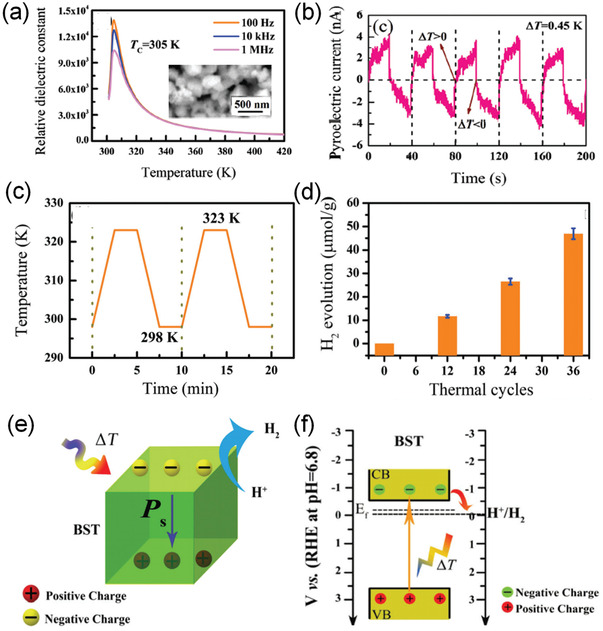
a) Temperature‐dependent dielectric constant of Ba_0.7_Sr_0.3_TiO_3_ (BST). b) The pyroelectric current under the temperature variation. c) Designed temperature variation in the experiments. d) H_2_ evolution amount as a function of thermal cycles. e) Illustrated working mechanism of pyrocatalytic H_2_ generation. (d) Schematic illustration of the energy band level in pyrocatalysis. Reproduced with permission.^[^ ^74^ ^]^ Copyright 2018, Royal Chemical Society.

Recently, Xu et al.^[^ ^74^ ^]^ reported a pyrocatalytic H_2_ generation by Ba_0.7_Sr_0.3_TiO_3_ (BST) nanoparticles. As shown in **Figure** **28**a, the *T*_C_ of the BST has been tuned to 305 K. Substantial and reproducible pyroelectric current can be achieved under thermal cycles (Figure 28b). Under temperature variation between 298 K to 323 K (Figure 28c), a high yielding of 46.89 µmol g^−1^ per thermal cycle can be achieved. The thermal cycles lead to the changes in spontaneous polarization. The pyroelectric‐induced electric potential makes the minimum of the conduction band of BST more negative than the conduction band of H^+^/H_2_. Then, the pyroelectric BST generates positive and negative charges on the surface of the material, as shown in Equation (7). The pyroelectric‐induced positive charges (*q* ^+^) can oxidize water molecules on the surface of BST to produce H^+^ and O_2_. The H^+^ can further react with the pyroelectric‐induced negative charge (*q* ^−^) to produce H_2_ (Equation 8).

(11) $$BST\overset{\Delta T}{\to }\mathrm{BST}+q^{+}+q^{-}$$

(12) $$2H^{+}+2q^{-}\to H_{2}$$

In addition to tuning the *T*_C_, the pyrocatalytic system can also be optimized to improve the pyrocatalytic H_2_ generation. Recently, Zhang et al.^[^ ^157^ ^]^ used a high impedance 0.5 m KOH electrolyte with working electrodes connected to a rectified pyroelectric harvester. A highest voltage of 2.34 V can be produced to drive H_2_ generation reactions. Besides the electrolyte, reducing the frequency of the temperature oscillations can also increase the pyroelectric charge and lead to higher potential difference. To this end, pyrocatalytic H_2_ generation provides a promising eco‐friendly way to harvest cold‐hot temperature fluctuation energy.

#### 5.1.5 Photo‐Piezo‐Cocatalytic H_2_ Generation

Conventionally, the photocatalytic efficiency is usually hindered by the photoinduced carrier recombination.^[^ ^158^ ^]^ There are only few carriers that can move to the surface of the ferroelectrics to develop the oxidation and reduction. Hence, it is still challengeable to obtain excellent catalytic activity for single photocatalysis.^[^ ^159^ ^]^ Ferroelectric‐based catalytic H_2_ generation plays as a game changer own to photo‐piezo cocatalysis.

**Figure 29:**
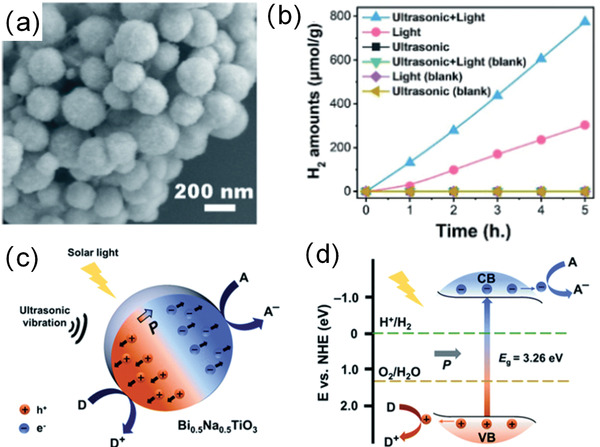
a) Morphology of Bi_0.5_Na_0.5_TiO_3_ nanoparticles and b) their photo‐piezo‐cocatalytic H_2_ generation under ultrasound+light conditions. c) Schematic illustration of the catalytic process and mechanism in Bi_0.5_Na_0.5_TiO_3_. Reproduced with permission.^[^ ^160^ ^]^ Copyright 2020, Royal Society of Chemistry.

As shown in **Figure** **29**, based on Bi_0.5_Na_0.5_TiO_3_ nanoparticle, Zhao et al.^[^ ^160^ ^]^ reported an enhanced H_2_ generation performance under light + ultrasonic vibration. The H_2_ generation performance increases as the size of the nanoparticle decreases, suggesting that proper size and shape are significant to improve the photo‐piezo‐cocatalytic properties. By applying the mechanical stress on the noncentral‐symmetric structures of ferroelectrics, piezoelectric‐induced electric potential can be obtained. The electric potential can promote the separation and transfer of charge carriers, including photo‐induced and piezoelectric‐induced charge carriers.^[^ ^161^, ^162^, ^163^ ^]^ The piezoelectric effect leads to band bending and high electric potential that is large enough to trigger an enhanced H_2_ generation in the name of photo‐piezo cocatalysis.

By choosing organic molecules with different charge properties, Feng et al.^[^ ^164^ ^]^ reported that the apparent quantum efficiency (AQE) of photocatalytic water splitting increases by 6.5 times via a synergistic modulation of the internal carriers and the interaction with the matrix. It has been proved that the size, shape, and crystal orientation have large influences on the piezoelectric properties of ferroelectrics by theoretical and experimental studies. The nanoscale ferroelectrics have large surface area and abundant active sites. Meanwhile, the easier deformation of nanosize ferroelectrics provides much larger piezoelectric potential, which can promote charge separation and further enhance the photo‐piezocatalytic performance.^[^ ^118^ ^]^ Yu et al.^[^ ^118^ ^]^ have chosen KNbO_3_ as a representative material to show that modulating the shape and size of ferroelectrics can induce different catalytic performances.

To optimize the performance, strategies such as forming semiconductor/ferroelectric hybrid structures in addition to the shape of the ferroelectric nanoparticle, have also been considered. Wang and Song^[^ ^158^ ^]^ have investigated a coupling effect through piezoelectric and photonic properties based on Ag_2_O/BaTiO_3_ hybrid heterostructures, which gives the name of photopiezoelectric effect. However, their photoexcited charge carriers are mainly from Ag_2_O. Therefore, the recombination rate of photopiezocatalysts can be effectively suppressed to enhance the performance. Guo et al.^[^ ^165^ ^]^ reported an enhanced photocatalytic H_2_ generation by plasmonic and piezotronic effects based on periodic Al/BaTiO_3_ heterostructures.

**Figure 30:**
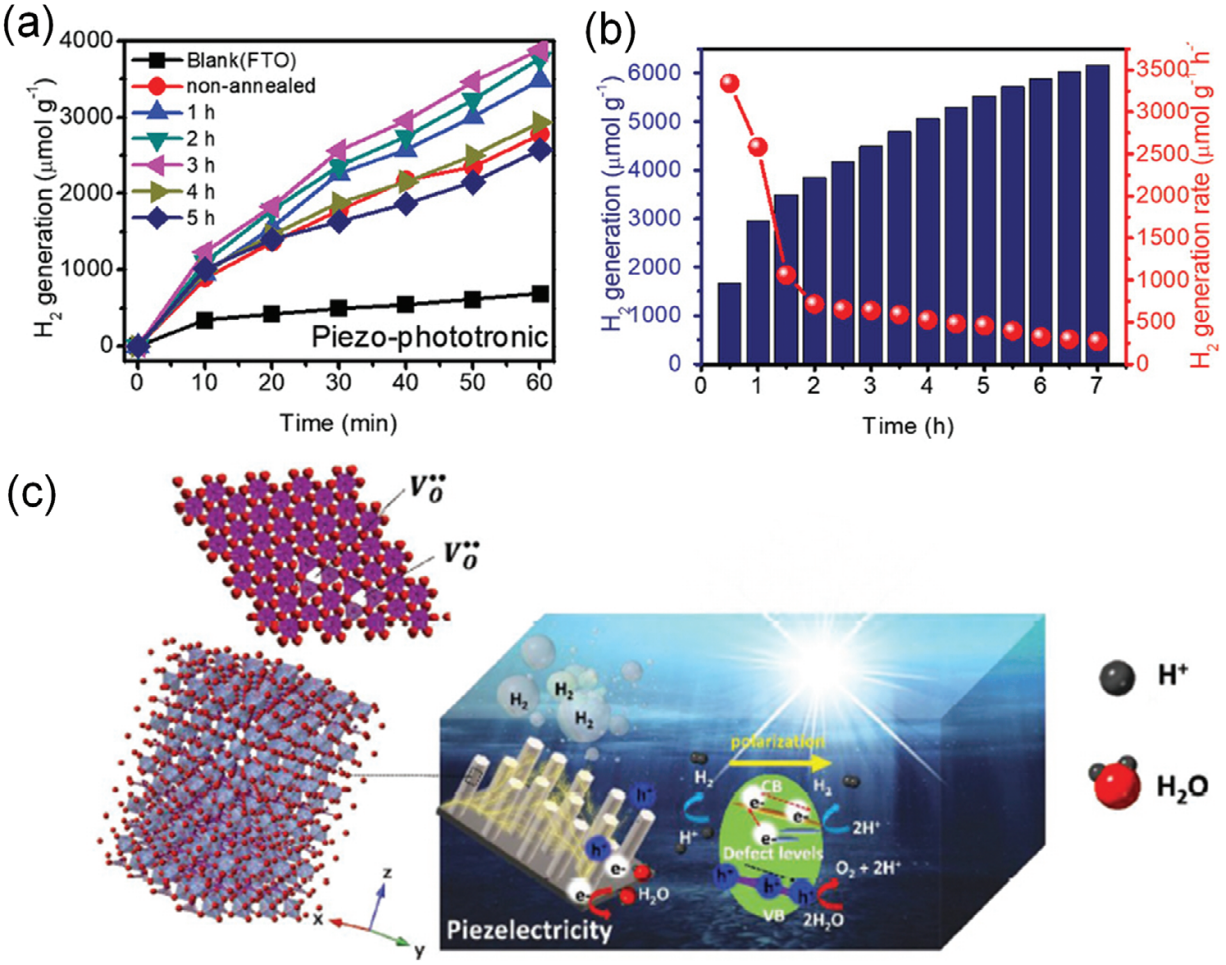
a) H_2_ generation amount as a function of reaction time by H_2_‐annealed samples with different oxygen vacancy concentrations controlled by annealing time b) H_2_ generation rate for samples with different annealing time and oxygen vacancy concentrations. c) The schematic illustration of photo‐piezo cocatalytic H_2_ generation mechanism. Reproduced with permission.^[^ ^142^ ^]^ Copyright 2020, Wiley.

However, in addition to the formation of semiconductor/ferroelectric heterostructure, an intrinsic way is to either increase the light absorption or piezoelectricity. For example, by introducing moderate oxygen vacancy concentration in ZnSnO_3_ controlled by H_2_‐annealing time, the H_2_ generation performance of ZnSnO_3_ can be significantly improved after annealing for 1 h (**Figure** **30**a).^[^ ^142^ ^]^ Figure 30b demonstrates that the maximum amount of H_2_ generation reaches ≈6000 µmol g^‐1^, which suggests the sample is able to withstand mechanical energy vibrations.

**Figure 31:**
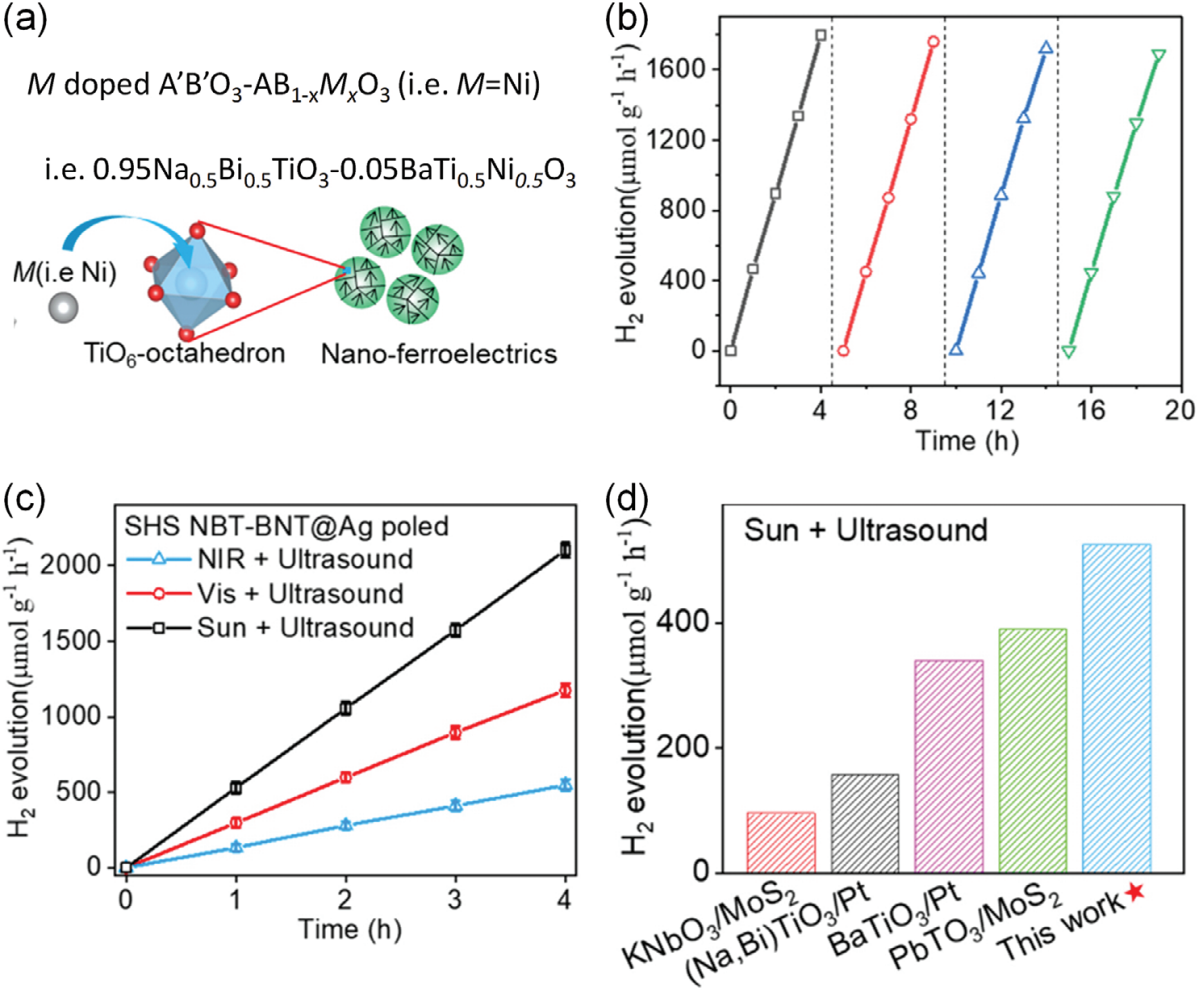
a) Illustration of doping in MPB compositions as *M* doped A'B’O_3_‐AB_1‐_ *_x_* M*_x_* O_3_ (i.e., M = Ni). Reproduced with permission.^[^ ^119^ ^]^ Copyright 2021, Elsevier.

In our recent work, we consider enhancing both the piezoelectricity and light absorption by using bandgap engineered ferroelectrics at the MPB composition.^[^ ^90^ ^]^ **Figure** **31**a demonstrates the defect‐engineered MPB composition as M doped A'B’O_3_‐AB_1‐_ *_x_* M*_x_* O_3_ (i.e., M = Ni). Ni doped 0.95Na_0.5_Bi_0.5_TiO_3_–0.05BaTi_0.5_Ni_0.5_O_3_ (0.95NBT‐0.05BNT) was taken as an example and gives a high H_2_ production rate of ≈450 µmol g^−1^ h^−1^ (Figure 31b).^[^ ^119^ ^]^ The photo‐piezo cocatalytic H_2_ generation is significantly higher compared with that reported in other systems without defect engineering, such as (Na,Bi)TiO_3_, BaTiO_3_, and so on. The research on the nano photoferroelectrics is anticipated to provide a new horizon to novel efficient photo‐piezo‐cocatalysts.

#### 5.1.6 Environmental Energy Harvester

The arrival of the Internet of Things (IoTs) has increasing demand for wireless, sustainable, and independent operation of sensor network and systems. Therefore, sensors with the ability to harvest environmental energy are promising. The most famous ferroelectric‐based energy harvester is known as piezoelectric nano generators (PENGs). The development of PENG has obtained rapid growth after the discovery of substantial transformation of mechanical force to electricity in piezoelectric 1D ZnO nanowires by Wang et al.^[^ ^161^ ^]^ The large group of ferroelectric materials provides chances to explore efficient PENGs.

**Figure 32:**
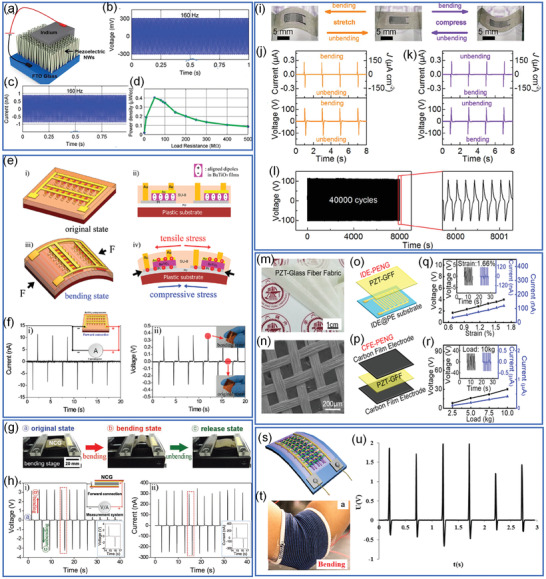
Schematic illustration of the nanogenerator based on a) vertically aligned BaTiO_3_ nanowire arrays. b) Voltage, c) current, and d) power density output measured at a resonant frequency of 160 Hz. Reproduced with permission.^[^ ^101^ ^]^ Copyright 2014, Royal Chemical Society. e) Schematic illustration of the flexible PENG based on patterned BaTiO_3_ thin films deposited on polyurethane‐coated plastic substrate (Kapton film). i,ii) Schematic illustrations of PENG’ and with longitudinally aligned dipole arrays in the original state without bending. iii,iv) An output voltage can be built through the Au electrodes between the interdigital electrodes. f) The output current i) and voltage ii) of the BaTiO_3_ based PENG during periodic bending‐unbending cycles when forward‐connected to the current meter (left inset shows the measurement setup and the right two insets show the nanogenerator in the bent and original states). Reproduced with permission.^[^ ^166^ ^]^ Copyright 2010, American Chemical Society. g) Photos of the BaTiO_3_/polymer nanocomposite‐based PENG in the original, bending, and release state. h) Output voltage and current signals of the PENG in the forward‐connection during the periodic bending‐unbending cycles. Reproduced with permission.^[^ ^167^ ^]^ Copyright 2012, Wiley. i) Photos of the flexible PENG based on PZT thin films deposited on 2D mica in original (left), flat (middle), and downward‐bending states (right). Output current (with cross‐sectional current density) and voltage of j) the PENG in stretch and k) compress mode, respectively. l) Output voltage under fatigue measurement condition (left) and a magnified plot of the output (right). Reproduced with permission.^[^ ^109^ ^]^ Copyright 2018 Elsevier. m) Photo and n) SEM image of superflexible PENG based on PZT–glass fiber fabric composite. Schematic illustration of the PENG fabricated with o) IDEs and p) top‐bottom electrodes. The output voltage as a function of the strain under the bending mode with q) bending angle varying from ≈62°–163.6° (corresponds to the strain range of 0.68%) and r) load. Reproduced with permission.^[^ ^168^ ^]^ Copyright 2019, Elsevier. s) Schematic diagram of the textile made from the BaTiO_3_ nanowires/PVC nanocomposites. t) Photo of the PENG made into a wearable cloth. u) The voltage output of the cloth‐like PENG. Reproduced with permission.^[^ ^103^ ^]^ Copyright 2015, Elsevier.

The early study on ferroelectric‐based PENGs mainly focuses on BaTiO_3_ nanoparticles and thin films. For example, as shown in **Figure** **32**a, Koka et al.^[^ ^101^ ^]^ have reported a vertically aligned BaTiO_3_ nanowire array on inorganic fluorine‐doped tin oxide‐coated (FTO) glass substrate. By poling the BaTiO_3_ nanowire arrays, an average power density of ≈6.27 W cm^‐3^ from 1 *g* acceleration can be obtained, which is ≈16 times larger than that with conventional ZnO nanowire arrays under the same acceleration input. To design efficient PENG, in addition to using nanowires with easy deformation and high piezoelectric output, depositing piezoelectric thin films on plastic substrates is another method. As shown in Figure 32e, Lee's group^[^ ^166^ ^]^ did a pioneering work by using BaTiO_3_ on a flexible plastic substrate to fabricate PENGs. The BaTiO_3_ thin films were first deposited on Si substrates and then transferred onto flexible substrate by photolithograph and etching process. The flexible PENG shows an output current density of 0.19 µA cm^‐2^ and a power density of ≈7 mW cm^‐2^, which is promising for flexible displays in touchable technologies. To further increase the flexibility of the PENG, Lee's group further reported^[^ ^167^ ^]^ a flexible nanocomposite generator made of BaTiO_3_ nanoparticles and graphitic carbons as shown in Figure 32g. The output voltage and current were measured under original, bending, and releasing states, respectively. An output voltage of ≈3.2 V and a current of ≈350 nA under periodic mechanical deformation to a strain of ≈0.33% (Figure 32h) were obtained, and a commercial light‐emitting diode can be lighted up by storing the energy in capacitors.

In addition to classical ferroelectric BaTiO_3_, highly piezoelectric ferroelectrics such as PZT at MPB composition have been widely used. As shown in Figure 32i–l, Wang et al.^[^ ^109^ ^]^ deposited PZT thin films on flexible 2D mica and fabricated an all‐inorganic flexible piezoelectric energy harvester. An outstanding performance (*V*_out_ ≈ 120 V, *I*_out_ ≈ 150 A cm^‐2^, and power density of 42.7 W cm^‐3^) can be obtained, which is comparable with those PENGs through conventional “growth‐transfer” method from inorganic rigid substrates to organic soft substrates. The all‐inorganic flexible piezoelectric energy harvester shows highly efficient transformation of mechanical force to electricity. However, the flexibility of the PENG is limited by the flexibility of mica where the bending angle is generally less than 90°. To break the limited bending angle of the all‐inorganic energy harvester, we proposed a superflexible substrate with 180° bending angle based on glass fiber fabric.^[^ ^168^ ^]^ Figure 32m,n shows the optical and SEM image of the PZT‐fiber fabric composite in the macroscale and microscale, respectively. The flexible PENG can be fabricated using either interdigital electrodes (IDEs) or top‐bottom electrodes. By using a 3.5 cm × 1.5 cm scale fabric, an efficient energy harvesting performance (≈60 V, ≈500 nA) with 180° bending angle was obtained. An 8 cm × 8 cm scale PENG can simultaneously light up 20 commercial green LEDs successfully as the human leg is in a bent and straightened position. Moreover, the superflexible PENG shows promising application in force sensor since there are linear voltage–load and voltage–strain relationships as shown in Figure 32q,r. Similarly flexible fabric‐based PENG but with organic matrix has also been proposed as shown in Figure 32s,t where the PENG can be made into wearable cloth (Figure 32u).^[^ ^103^ ^]^

**Figure 33:**
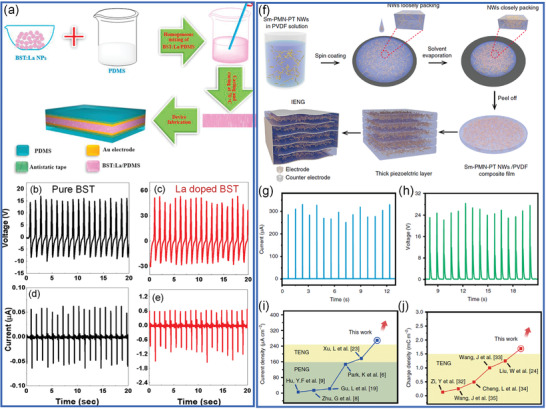
a) Illustrated preparation process for La doped BaSrTiO_3_ nanoparticles (BST:La NPs) and its PENG device. b,c) *V*_OC_ and d,e) *I*_SC_ as a function of time with the application of constant mechanical loading of 5 N based on the same composite thickness and active area (1.2 cm × 1.2 cm). Reproduced with permission.^[^ ^169^ ^]^ Copyright 2021, Elsevier. f) Illustrated 3D multilayer PENG preparation process by Sm doped PMN‐PT nanowires (Sm‐PMN‐PT NWs) and PVDF matrix. g) Output current and voltage of the multilayer PENG. Comparison of i) the current density and j) charge density of the multilayer PENG devices with other nanogenerators. Reproduced with permission.^[^ ^170^ ^]^ Copyright 2020, Springer Nature.

To improve the energy harvesting performance of PENG, defect‐engineered ferroelectrics have gained increasing attention in PENG devices. As shown in **Figure** **33**a, Khatua et al.^[^ ^169^ ^]^ prepared a BST:La NPs/PDMS nanocomposite through a solution casting method. It is found that the power density with the La‐doped BST increases by ≈10.5 times, compared with that of the undoped case (Figure 33b–e). More recently, as shown in Figure 33f, Gu et al.^[^ ^170^ ^]^ even reported a novel 3D design of flexible PENG by forming multilayer Sm‐PMN‐PT/PVDF nanocomposite structure. The novel design demonstrates a current density of ≈290 µA cm^‐2^, which is ≈1.93 and ≈1.61 times higher than the record values of PENGs and triboelectric nanogenerators (TENGs) (Figure 33g–j), respectively. These researches would be promising for the design of high‐performance flexible PENGs.

**Figure 34:**
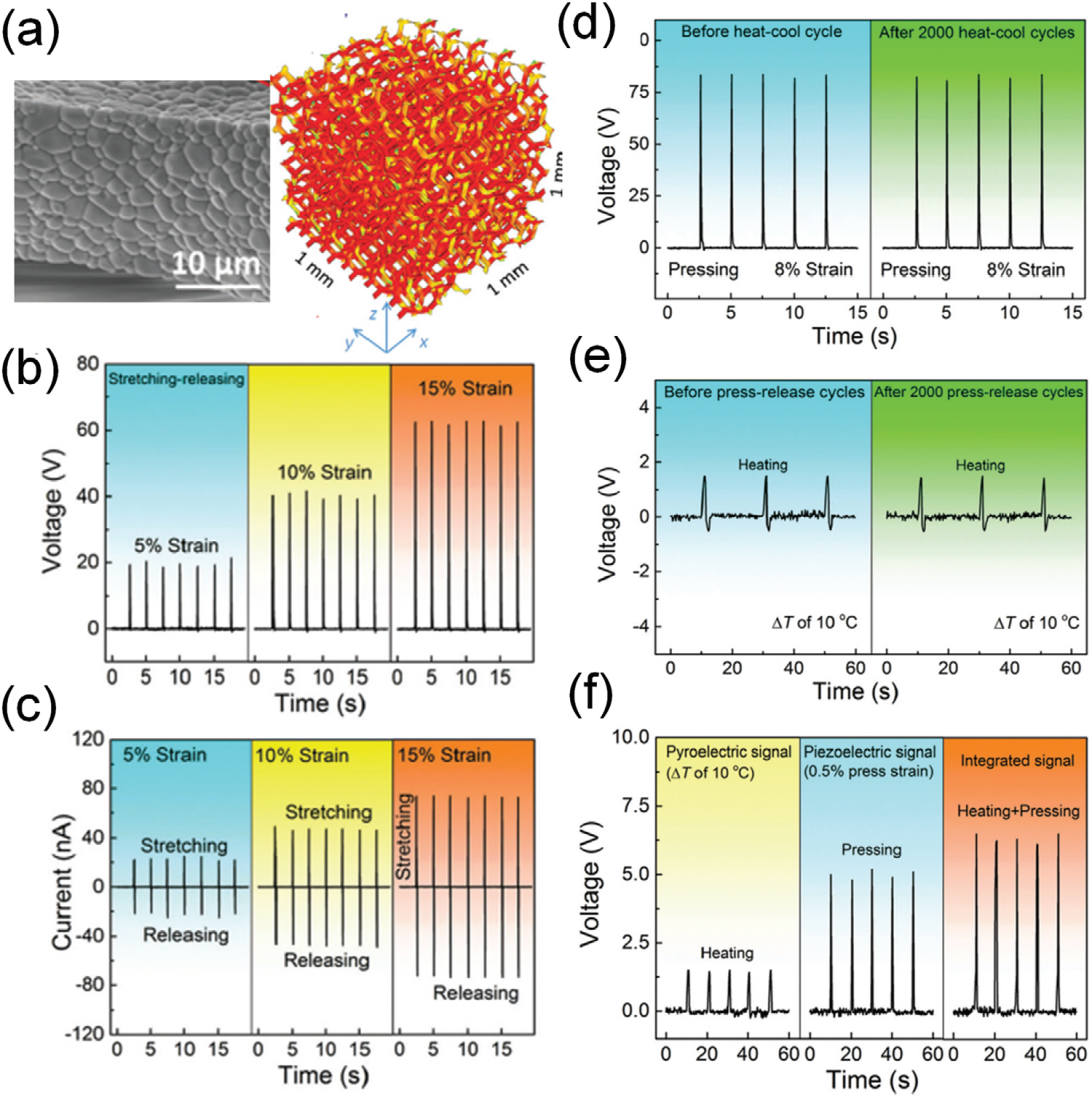
a) Magnified SEM image and schematic illustration of the 3D ceramic foam nanocomposite. The b) output voltage and c) current of the 3D composite under different stretching strain. The piezoelectric and pyroelectric voltages of the 3D composite before and d) after 2000 heating–cooling cycles (Δ*T* of 10 °C) and e) 2000 pressing‐releasing cycles (8% strain). f) The individual and superimposed piezoelectric‐pyroelectric induced output voltage of the 3D composites. Reproduced with permission.^[^ ^171^ ^]^ Copyright 2018, Royal Chemical Society.

In order to improve the number of energy sources, Zhang et al.^[^ ^171^ ^]^ recently reported a flexible 3D PENG based on 3D interconnected piezoelectric ceramic foam. **Figure** **34**a shows the SEM image of the foam and its schematic illustration. From Figure 34b,c, substantial output voltage *V*_out_ (≈22 to ≈65 V) and output current *I*_out_ (≈25 to ≈75 nA) were achieved. Figure 34d shows the performance of the 3D nanocomposite under pressing/strain cycles and heating–cooling cycles, the results are reproducible as proved by Figure 34e. Different from the current ceramic/polymer nanocomposites added with ferroelectric nanoparticles which is limited by the poor load‐transfer efficiency, the 3D foam based on 3D interconnected piezoelectric microfoams exhibits exceptional piezoelectric response while maintaining high mechanical durability. More importantly, the design shows excellent pyroelectricity, leading to a promising/novel design of ferroelectric‐based energy harvester for harvesting mechanical force and heat.

**Figure 35:**
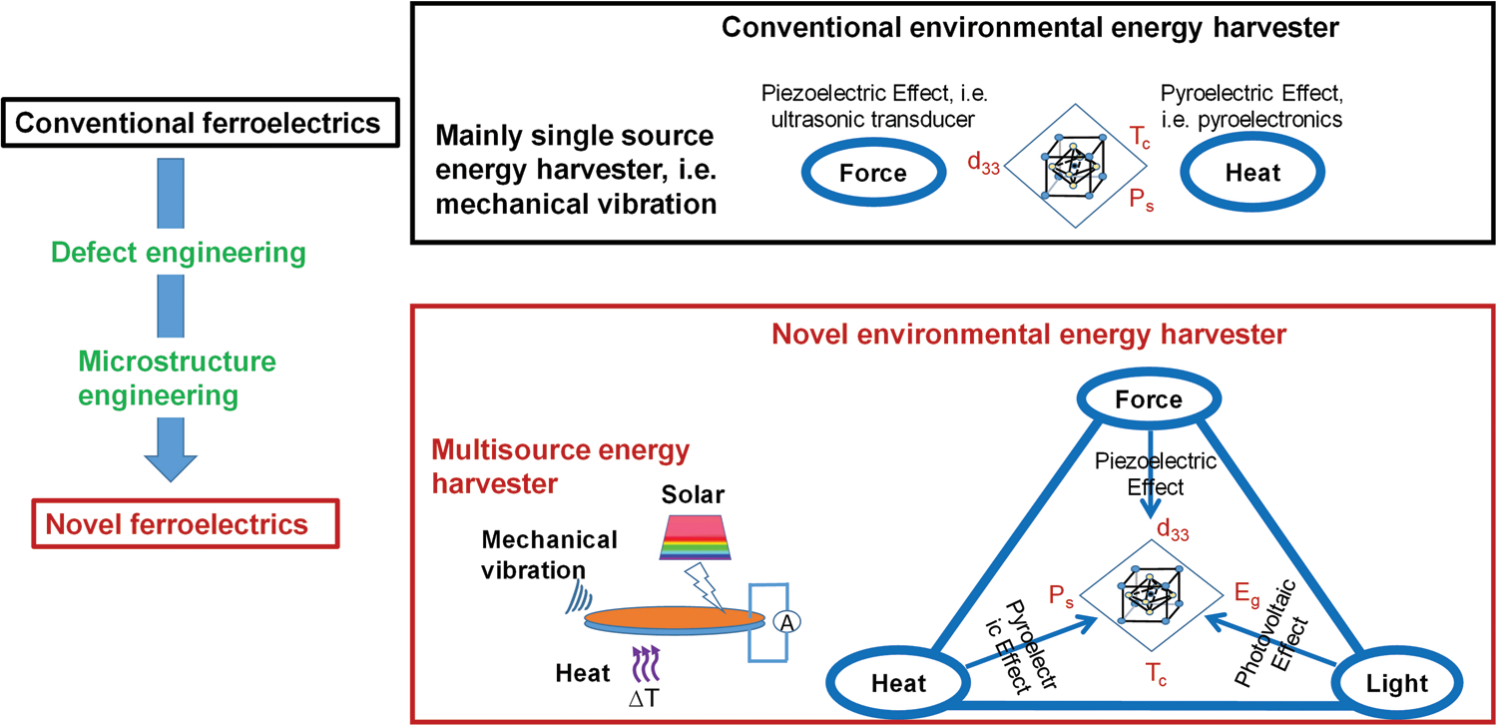
Schematic evolution of environmental energy harvester from mainly single‐source energy harvester to multisource energy harvester enabled by the defect/microstructure engineered novel ferroelectrics.

**Figure 36:**
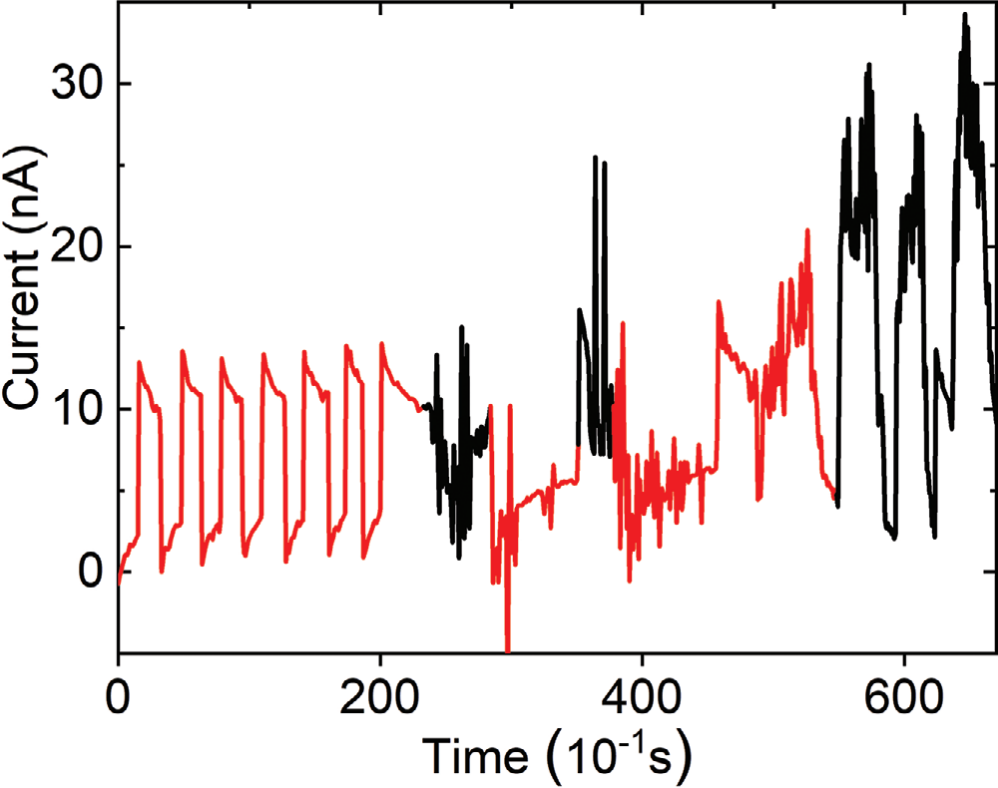
Multisignal response current of 0.95NBT‐0.05BNT. The energy signal including light, impact, and hot wind. The lines (red, blue, and green) belong to response current to single energy signal while the dark lines correspond to multienergy signals (impact + light and hot wind + light). Reproduced with permission.^[^ ^90^ ^]^ Copyright 2019, Wiley.

As shown in **Figure** **35**, the defect/microstructure engineered ferroelectrics give the chance to develop multisource energy harvester, compared with conventional single‐source energy harvester. This is further promoted by the discovery of highly piezoelectric and UV–Vis–NIR light‐responsive photoferroelectrics.^[^ ^92^, ^93^, ^132^ ^]^ As shown in **Figure** **36**, by engineering the bandgap of NBT‐BNT with Ni doping, we successfully demonstrated a multisource energy harvesting of light, impact, and heat.^[^ ^90^ ^]^ Similar multisource energy harvester has been also found in Ni‐doped PZT, and Ni‐doped KN‐BNNO ceramics.^[^ ^91^, ^92^, ^93^ ^]^

#### 5.1.7 Self‐Powered Environmental Signal Sensing

**Figure 37:**
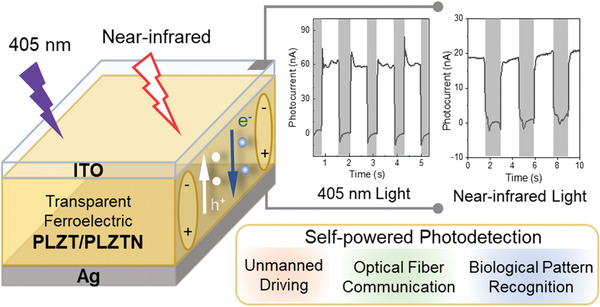
Visible or near‐infrared light self‐powered photodetectors based on transparent ferroelectric ceramics. Reproduced with permission.^[^ ^172^ ^]^ Copyright 2020, American Chemical Society.

The enhanced/novel properties of photoferroelectrics make environmental stimulations (light, mechanical vibration, and heat) as energy sources to be detectable. For example, as shown in **Figure** **37**, we reported a visible or near‐infrared light self‐powered photodetector based on transparent photoferroelectrics by using Ni doped lead lanthanum zirconate titanate (PLZNT) transparent ceramics.^[^ ^172^ ^]^ By using Sr doped BaTiO_3_ thin films deposited on glass fiber fabrics, we reported a superflexible PENG based self‐powered sensor that is highly sensitive to human motion monitoring.^[^ ^173^ ^]^ Bai et al.^[^ ^91^, ^93^ ^]^ have reported that the photoferroelectrics can also be used for multisource energy harvesting‐sensing.

These recent works suggest that the discovery of photoferroelectrics with outstanding light absorption significantly pave the way for conceptually novel multisource‐energy‐powered signal sensing.

### 5.2 Coping with Environmental Pollution

#### 5.2.1 Dye Degradation

##### Photocatalytic Dye Degradation

**Figure 38:**
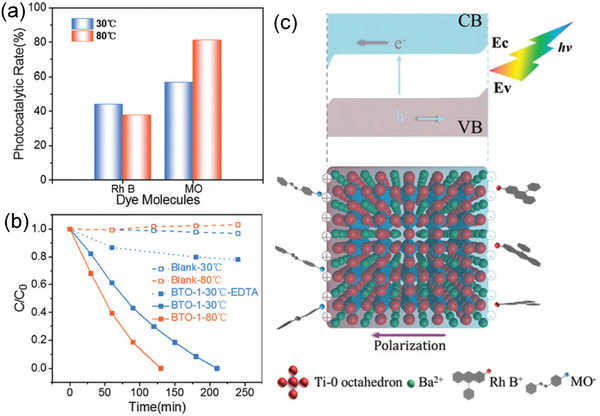
a) Photocatalytic degradation rate of MO and RhB after 90 min irradiation at 30 °C and 80 °C, respectively, the BaTiO_3_ NPs were synthesized with surfactant (BTO‐1). b) Photodegradation curves of MO with BTO‐1. c) Schematic illustration of the photocatalytic mechanism of BTO‐1. Reproduced with permission.^[^ ^178^ ^]^ Copyright 2018, Royal Society of Chemistry.

The spontaneous polarization of ferroelectrics facilitates the separation of charge carriers and back reactions in case of semiconductors and heterogeneous catalysts.^[^ ^174^, ^175^ ^]^ The continuousness of electric dipole–dipole interactions in surface chemistry makes ferroelectrics promising for photocatalysis.^[^ ^47^, ^176^, ^177^ ^]^ He et al.^[^ ^178^ ^]^ reported substantial photocatalytic methyl orange (MO) and RhB degradations by using BaTiO_3_ nanoparticles synthesized with surfactant (BTO‐1) measured in the temperature range of 30 °C to 80 °C (**Figure** **38**a,b). As shown in Figure 38c, the reaction involves the charge separation and preferential adsorption of charged dye species in the aqueous solution on the polar surfaces of BaTiO_3_ particles. The result suggests that polarity match should be considered in the experimental design.

In photocatalytic dye degradation, active agents such as superoxide radicals (•O_2_^−^) and hydroxyl radicals (•OH) react with organic dyes (i.e., RhB). The series of chemical reactions for the photocatalysis can be expressed as Equation 13:

(13) $$\mathrm{BTO}\overset{\mathrm{irridiation}}{\to }\mathrm{BTO}\left(h^{+}+e^{-}\right)$$

The general *h* ^+^ can directly attract the OH^−^ to produce ^•^OH and the O_2_ dissolved in the solution can react with the generated *e* ^−^ to produce O_2_^−^ as shown in Equations ((14), (15)):

(14) $$OH^{-}+h^{+}\to {}^{•}\mathrm{OH}$$

(15) $$O_{2}+e^{-}\to {O_{2}}^{-}$$

^•^OH and O_2_^−^ are strongly active species and will further react with the dyes to decompose the organic molecules based on Equation 16:

(16) $${}^{•}\mathrm{OH}/{O_{2}}^{-}+\mathrm{dyes}\to \mathrm{dye}\;\mathrm{decomposition}$$

However, most ferroelectrics are UV photocatalysts, which limit their applications. Tremendous methods have been used to improve its photocatalytic performance. For example, by doping BaTiO_3_ with some rare earth elements (i.e., Ce), strong redox with oxygen transport properties can be obtained. Acceptor doping can form defect pairs to improve redox properties.^[^ ^179^ ^]^ An intrinsic way to increase the photocatalytic performance is to engineer the bandgap of the ferroelectrics. Recently, we proposed a bandgap‐engineering strategy for highly piezoelectric and UV–Vis–NIR light‐responsive photoferroelectrics by doping the MPB relaxor ferroelectric ceramics with transition metal ions.^[^ ^90^ ^]^ Inspired by this kind of photoferroelectrics, based on SHS methods, we synthesized nanosize photoferroelectrics via SHS method. The nanosized photoferroelectrics show a significantly enhanced photocatalytic organic degradation rate, compared with that before bandgap engineering.^[^ ^119^ ^]^

**Figure 39:**
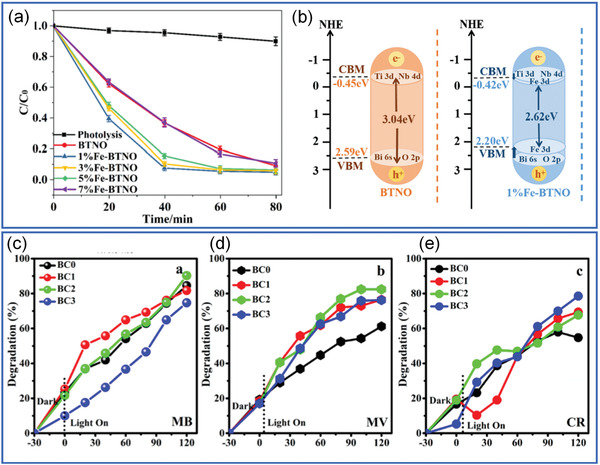
a) Photocatalytic degradation curves of RhB under visible‐light for Fe doped Bi_3_Ti_1‐_ *_x_* Fe*_x_* NbO_9_ (Fe‐BTNO, *x* = 0, 1%, 3%, 5%, and 7%). b) Energy band diagram after Fe doping. Reproduced with permission.^[^ ^183^ ^]^ Copyright 2022, Elsevier. Photocatalytic degradation of c) RhB, d) MV, and e) CR. MB: methylene blue; MV: methyl violet; (CR): congo red. Reproduced with permission.^[^ ^182^ ^]^ Copyright 2019, American Chemical Society.

By defect engineering, both the photocatalytic degradation and energy harvesting of ferroelectrics can be further enhanced.^[^ ^119^, ^143^, ^180^, ^181^, ^182^, ^183^ ^]^ As shown in **Figure** **39**a, Cui et al.^[^ ^183^ ^]^ reported an obviously enhanced photodegradation of RhB by using Fe doped BTNO. Figure 39b shows that the doping of Fe in the system can narrow the bandgap due to the defect‐induced gap‐states, leading to an enlarged photocatalytic performance. Similar results have also been reported in Ce doped BaTiO_3_.^[^ ^182^ ^]^ As shown in Figure 39c,d, moderate doping concentration of Ce in BaTiO_3_ can obviously enhance the photocatalytic degradation of MB, MV, and CR, compared with the pure one and the one with excess doping. Therefore, defect engineering is very helpful to introduce gap‐states and enhance the solar light absorption.

##### Piezocatalytic Dye Degradation

**Figure 40:**
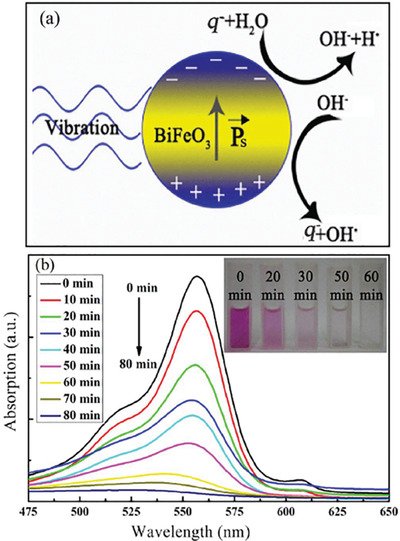
a) Schematic illustration of the piezocatalytic dye degradation mechanism, b) the absorption spectra of RhB solutions under ultrasonic vibration with BiFeO_3_ catalysts. Reproduced with permission.^[^ ^114^ ^]^ Copyright 2017, Elsevier.

By harvesting vibration energy, piezocatalysis can directly transfer mechanical energy into chemical energy.^[^ ^184^, ^185^ ^]^ It is well accepted that piezoelectric potential could be induced in the noncentrosymmetric materials under vibration.^[^ ^186^ ^]^ An internal electric field was induced and extended across the entire grain. As shown in **Figure** **40**, You et al.^[^ ^114^ ^]^ reported that a strong piezo‐electrochemical effect can be obtained based on multiferroic BiFeO_3_ square micro‐sheets, in which ≈95% mechano‐catalytic RhB decomposition ratio is obtained (≈10 mg L^‐1^). Middle active species such as OH**_˙_** were observed, which suggests a strong piezo‐electrochemical coupling in BiFeO_3_ microsheets. As shown in chemical reaction Equation 17, the negative charge on the BiFeO_3_ can react with H_2_O and generate H^•^and OH^−^:

(17) $$q^{-}+H_{2}O\to OH^{-}+H^{•}$$

Then OH**_˙_** can be released by the positive charges on the surface of the ferroelectrics:

(18) $$OH^{-}\to \;q^{-}+4OH^{•}$$

Since OH**_˙_** is strongly oxidative and can decompose dye molecules, the dyes can be completely degraded as shown in Equation 19:

(19) $$\;OH^{•}+\mathrm{dye}\;\mathrm{molecules}\to \mathrm{dyes}\;\mathrm{decomposition}$$

The free charge carriers are separated by the strong depolarization electric field, which resembles the process promoted by a constant electrical bias. The electrons and holes formed on the opposite surfaces of the catalysts can produce superoxide radicals or hydroxyl radicals with dissolved oxygen and water, respectively.^[^ ^187^, ^188^ ^]^ Besides, the built‐in electric field contributes to the bending of the valence band and conduction band, which is considered to play a vital role in promoting the charge transportation efficiency and catalytic performance.^[^ ^189^ ^]^ As reported by Wu et al.,^[^ ^190^ ^]^ the slope for the tilt of the band is proportional to the piezoelectric potential of the material.

**Figure 41:**
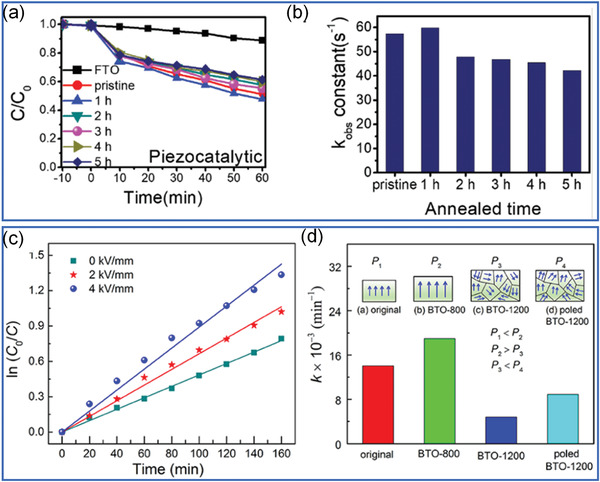
a) Piezocatalytic degradation amount and b) rate of ZnSnO_3_ with different oxygen vacancy concentrations controlled by different annealing time in H_2_ atmosphere. Reproduced with permission.^[^ ^142^ ^]^ Copyright 2020, Wiley. c) Piezocatalytic degradation behavior of microstructure‐engineered BaTiO_3_ NPs under different annealing temperature. Reproduced with permission.^[^ ^191^ ^]^ Copyright 2018, Royal Chemistry Society.

For piezocatalysis, the key role is the piezoelectricity. For example, as shown in **Figure** **41**a,b, by doping moderate oxygen vacancy concentration in the ZnSnO_3_ system, the donor levels can facilitate the separation of charges induced by piezoelectric effect.^[^ ^142^ ^]^ In addition to defect engineering, Wu et al.^[^ ^191^ ^]^ prepared BaTiO_3_ with different microstructures by different annealing temperatures and poling conditions as shown in Figure 41c,d. It is found that the piezocatalytic degradation performance can be improved by optimizing the microstructure as well. Phuong et al.^[^ ^192^ ^]^ demonstrated an enhanced piezocatalytic performance for water treatment near *T*_C_ since where large changes in polarization under stress or temperature variation can be induced. Moreover, an extrinsic contributions associated with domain wall motion would also promote the piezocatalysis.^[^ ^193^ ^]^ More recently, Wang et al.^[^ ^194^ ^]^ have reported that piezocatalysis can be used for nondestructive tooth whitening. The teeth contaminated by black tea, blueberry juice, wine, or a combination of those can be obviously whitened by the poled BaTiO_3_ nanoparticles after vibration for 3h whilst low damage to the enamel and biological cells. These researches suggest that piezocatalysis is not only promising for just dye degradation but also for tooth whitening.

##### Pyrocatalytic Dye Degradation

**Figure 42:**
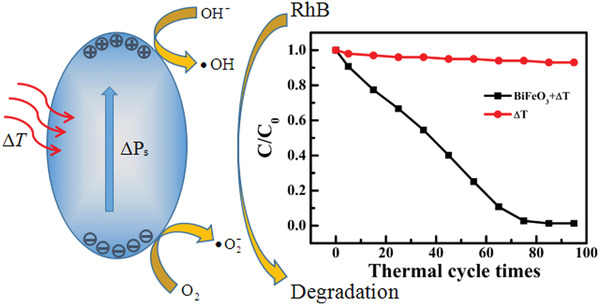
Schematic illustration of the pyrocatalytic dye degradation by using BiFeO_3_ nanoparticles. Reproduced with permission.^[^ ^195^ ^]^ Copyright 2016, Royal Chemistry Society.

As we detailed before, pyrocatalysis is another way of energy harvesting for dye degradation. Pyrocatalysis provides a sustainable way for reusable wastewater treatment technology through utilizing environmental day‐night temperature variations. As shown in **Figure** **42**, Wu et al.^[^ ^195^ ^]^ reported strong pyrocatalytic dye degradations with an ultrahigh degradation efficiency (90%) by using BiFeO_3_ nanoparticles under room temperature cold‐hot alternating excitations (between 27 °C and 38 °C). In addition to BiFeO_3_, the pyrocatalytic behavior of BaTiO_3_ has also been widely reported.^[^ ^196^, ^197^ ^]^ The pyroelectric catalytic reaction can be described as Equations ((20), (21), (22), (23)):

(20) $$\mathrm{BaTi}O_{3}\overset{\Delta \;T}{\to }\mathrm{BaTi}O_{3}\left(q^{+}+q^{-}\right)$$

(21) $$OH^{-}+q^{+}\to {}^{•}\mathrm{OH}$$

(22) $$O_{2}+q^{-}\to {O_{2}}^{-}$$

(23) $$OH^{•}/{O_{2}}^{-}+\mathrm{dyes}\to \;\text{dye}\;\mathrm{decomposition}$$

**Figure 43:**
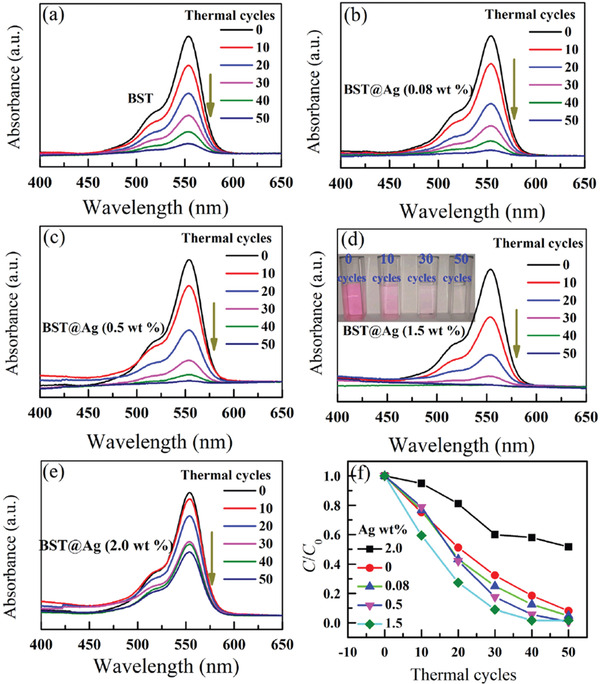
a–e) Absorption spectra of RhB solution mixed with Sr doped BaTiO_3_ nanoparticles coated with different concentration of Ag (BST@Ag) (0, 0.08, 0.5, 1.5, 2.0 wt%) under different thermal cycles. f) The degradation efficiency for different concentration of BST@Ag. Reproduced with permission.^[^ ^199^ ^]^ Copyright 2018, Elsevier.

Compared with photocatalysis and piezocatalysis, pyrocatalysis is decided by the variation of polarization which is generally much higher near *T*_C_.^[^ ^198^ ^]^ However, as shown in Figure 27, most high‐performance or widely used ferroelectrics show their *T*_C_ far from room temperature. By using defect engineering, Xu et al.^[^ ^199^ ^]^ reported a strong pyrocatalytic degradation efficiency of ≈99% for RhB by using Sr doped BaTiO_3_ coated with Ag electrode (BST@Ag). The doping of Sr effectively tuned the *T*_c_ close to room temperature (**Figure** **43**). The results also indicate the important role of Ag in improving the pyrocatalytic performance. This work suggests that defect‐engineered ferroelectrics is promising to explore high‐performance pyrocatalysis.

##### Photo‐Piezo‐Cocatalytic Dye Degradation

The photo‐piezo‐cocatalytic behavior can not only drive H_2_ generation process, but also be used to drive dye degradation upon coupling of solar and mechanical vibration.

**Figure 44:**
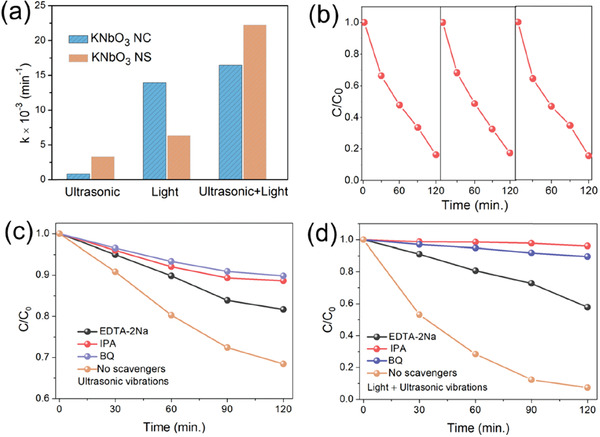
a) RhB degradation reaction kinetics based on KNbO_3_ nanocube (NC) and nanosheet (NS). b) The dye degradation cycling performance of the KNbO_3_ nanosheet under ultrasonic vibration + light illumination. Active species trapping degradation experiments for c) KNbO_3_ NS under ultrasound vibration and d) ultrasonic vibration + light illumination. Reproduced with permission.^[^ ^118^ ^]^ Copyright 2019, Elsevier.

The synergistic effect of photo‐piezo‐cocatalytic process provides a unique opportunity to enhance both H_2_ generation and organic dye degradation. Yu et al.^[^ ^118^ ^]^ took the KNbO_3_ as an example and further considered the effect of shape based on nanosheet (NS) and nanocube (NC). As shown in **Figure** **44**, it is obvious that the KNbO_3_ shows an enhanced reproducible performance of dye degradation under ultrasonic vibration + light illumination. The KNbO_3_ NS shows much higher performance due to much easier deformation under ultrasonic vibration, compared with that of the NC counterpart. The photo‐piezo‐cocatalytic performance can be further enhanced by forming a semiconductor/ferroelectric hybrid structure. For example, Li et al.^[^ ^158^ ^]^ reported that the RhB degradation performance of BaTiO_3_–Ag_2_O under ultrasound+illumination is much higher than that under single vibration or light illumination. The photo‐piezo‐cocatalysis was further demonstrated based on simulation result. The periodic acoustic pressure of ultrasonic wave and the extreme pressure from cavitation walls will induce a piezoelectric potential of ≈0.36 V inside BaTiO_3_ nanotubes along its spontaneous polarization direction, which is a rechargeable process for ferroelectrics. The polarization‐induced electric field can provide a driving force to separate the holes and electrons. As a result, the designed ferroelectrics can remain active for photo‐piezo‐cocatalysis. With this synergistic effect in mind, many researchers have developed more specific ferroelectrics for photo‐piezo‐cocatalysis.

**Figure 45:**
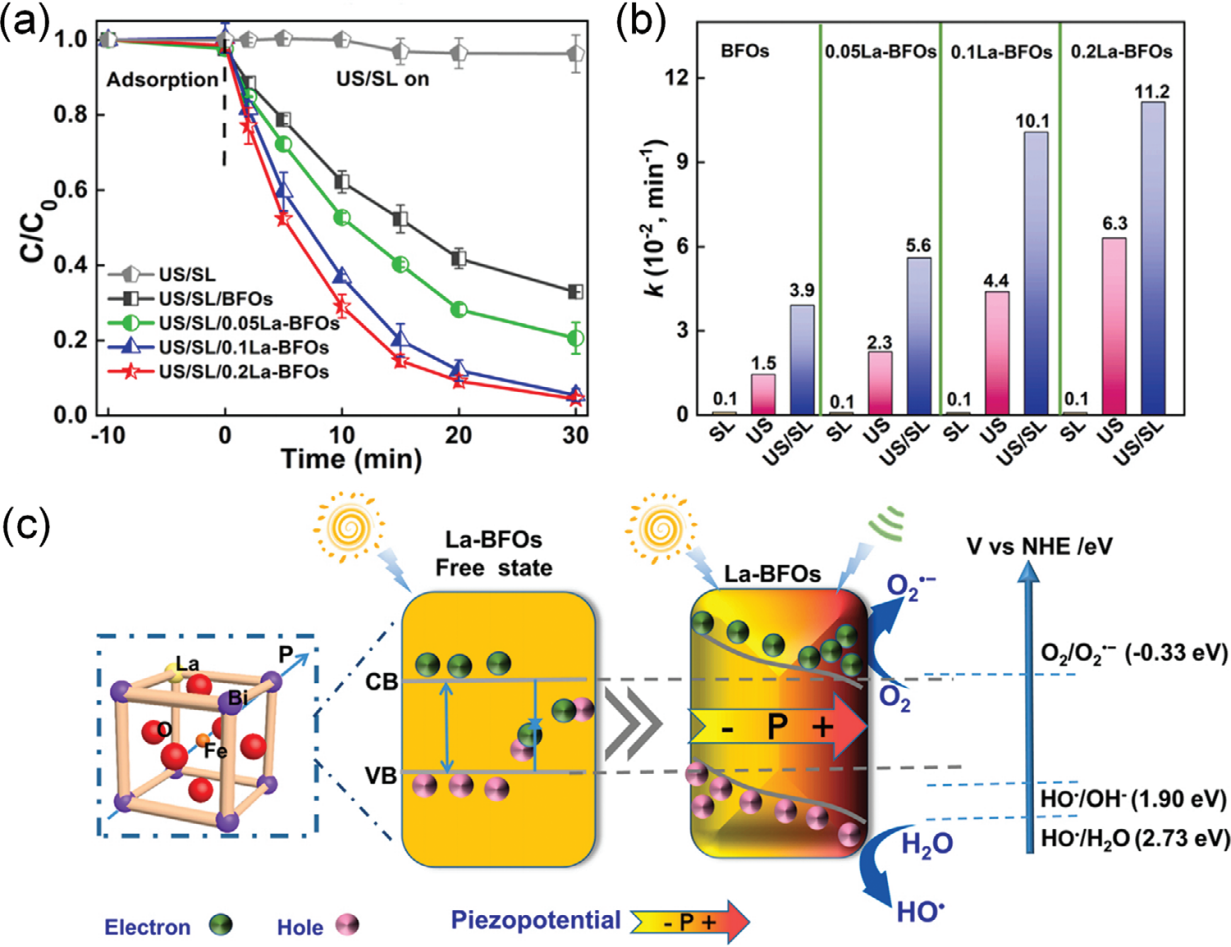
a) Photo‐piezo‐cocatalytic degradation of carbamazepine (CBZ) by La doped BiFeO_3_ (La‐BFOs) under ultrasound (US), solar light (SL), and US/SL conditions. b) Photo‐piezo‐cocatalytic CBZ degradation (TBA for HO^•^, p‐BQ for O_2_^•^, L‐his for ^1^O_2_, K_2_Cr_2_O_7_ for e^−^ and EDTA‐2Na for h^+^. c) Schematic illustration of the photo‐piezo‐cocatalysis mechanism. Reproduced with permission.^[^ ^181^ ^]^ Copyright 2022, Elsevier.

To intrinsically improve the photo‐piezo‐cocatalytic performance, bandgap, and piezoelectricity should be vital. Lan et al.^[^ ^181^ ^]^ reported a significantly enhanced photo‐piezo‐cocatalytic performance in La doped BiFeO_3_ with different doping levels and under different stimulations (**Figure** **45**a,b). Figure 45c shows that photocatalysis can break its limit after piezoelectric‐induced electric potential was introduced. Under ultrasound vibration, piezoelectric‐induced electric potential can be triggered to make a slight shift in the onset potential of current densities. The band bending can lift CB band edge to be more negative than the O_2_/O_2_^•^ redox potential, while VB band edge becomes more positive than HO^•^/OH^−^ or HO^•^/H_2_O redox potential. Moreover, the piezoelectric‐induced electric potential assists the charge separation and significantly enhances the CBZ degradation.

**Figure 46:**
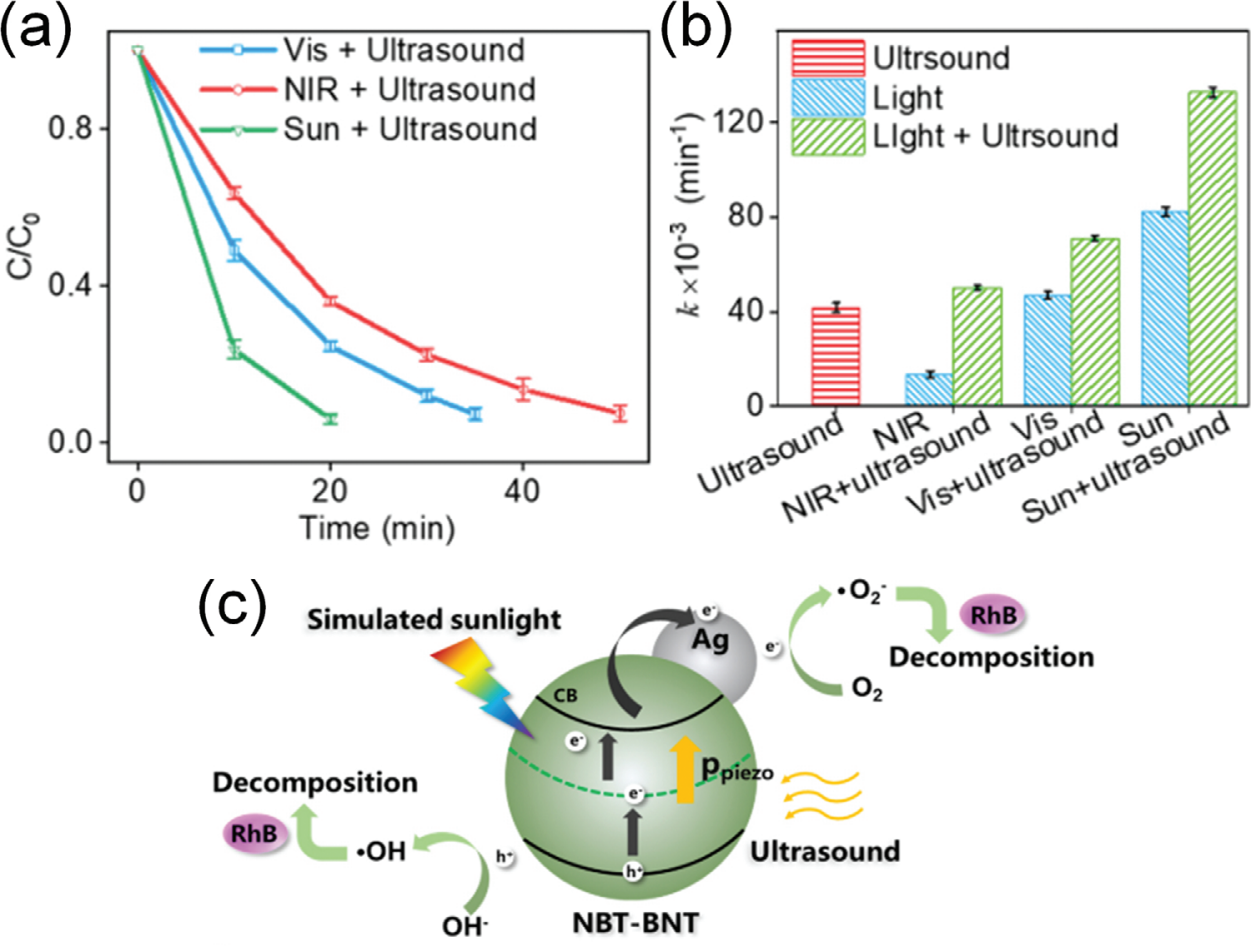
a) Photocatalytic RhB degradation performance of Ni doped Na_0.5_Bi_0.5_TiO_3_‐BaNi_0.5_Ti_0.5_O_3_ nanoparticles (NBT‐BNT NPs) under light and ultrasound vibration. b) Photo‐piezo‐cocatalytic RhB degradation rate under light + ultrasound vibration. c) Schematic illustration of the reaction mechanism by photo‐piezo‐cocatalysis. Reproduced with permission.^[^ ^119^ ^]^ Copyright 2021, Elsevier.

Recently, armed with photoferroelectrics with UV–Vis–NIR light absorption,^[^ ^90^ ^]^ we studied their photo‐piezo‐cocatalytic dye degradation behavior. By using Ni doped Na_0.5_Bi_0.5_TiO_3_‐BaNi_0.5_Ti_0.5_O_3_ nanoparticles (NBT‐BNT NPs) as an example, high‐performance RhB degradation can be achieved (**Figure** **46**a).^[^ ^119^ ^]^ The degradation rate (>100 10^−3^ min^−1^) under sun light + ultrasound reaches an optimal level (Figure 46b), which is significantly higher than that of the single‐source case and also higher than that of the La‐doped BiFeO_3_.^[^ ^181^ ^]^ The much narrow sub‐bandgap introduced by defects and higher piezoelectricity enabled by the MPB composition are responsible for the higher photo‐piezo‐cocatalytic performance. As shown in Figure 46c, the much narrow bandgap of 2.45 eV together with two additional sub‐bandgaps of 1.00 eV and 1.45 eV contribute to the enhanced light absorption. Considering the large family of MPB relaxor ferroelectrics that can be bandgap‐engineered, more research on the multiple‐catalytic dye degradation is highly expected.

##### Piezo‐Pyro‐Cocatalytic Dye Degradation

**Figure 47:**
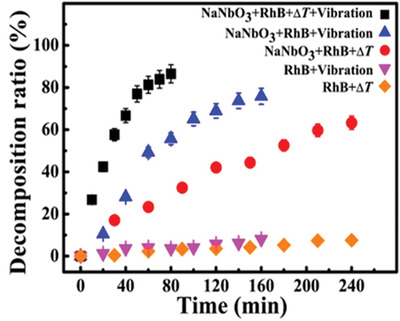
The piezo‐pyro‐cocatalytic performance for NaNbO_3_. Reproduced with permission.^[^ ^61^ ^]^ Copyright 2018, Elsevier.

From a practical application perspective, the key point is to further enhance the decomposition efficiency. Harvesting multisource energies have been proved to effectively increase the total degradation efficiency.^[^ ^61^, ^158^, ^200^, ^201^, ^202^ ^]^ Considering that all the ferroelectric materials exhibit pyroelectric properties, it is feasible to realize a highly efficient piezo/pyro‐bicatalysis for water treatment.^[^ ^61^, ^201^, ^203^ ^]^ You et al.^[^ ^61^ ^]^ investigated the catalytic dye degradation of NaNbO_3_ nanofibers under vibration + heat. As shown in **Figure** **47**, under ultrasonic vibration and temperature fluctuation (15–50 °C), the efficiency for the piezo‐pyro‐cocatalysis is reported to be ≈86.5%, which is much higher than that of pyrocatalysis (≈63.3%) and piezocatalysis (≈75.8%). Also, they proposed that the enhanced degradation efficiency via piezo‐pyro‐cocatalysis is mainly from the synergistic effect of piezocatalysis and pyrocatalysis rather than a simple additive effect. The abundant thermal energy in our environment makes piezo‐pyro‐cocatalytic dye degradation a sustainable way to cope with environmental pollution.

**Figure 48:**
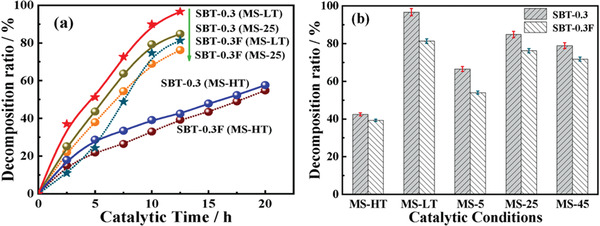
(a) Pyrocatalytic RhB degradation performance of Sr doped Ba_0.7_Sr_0.3_TiO_3_ (SBT) with (SBT‐0.3F) and without surfactant (SBT‐0.3) under conditions of magnetic stirring (MS), low temperature (LT), and high temperature (HT). b) Comparison of the pyrocatalytic RhB degradation of SBT‐0.3 and SBT‐0.3F under different conditions. Reproduced with permission.^[^ ^191^ ^]^ Copyright 2021, Elsevier.

To make full use of the pyrocatalysis, high pyroelectric coefficient would be favorable. Recently, by doping the BaTiO_3_ with Sr, superior pyeoelectric coefficient of 8 nC/(cm^2^K^1^) can be achieved.^[^ ^74^, ^199^ ^]^ Inspired by the enhanced pyroelectric effect in Sr doped BaTiO_3_ (SBT), Yang et al.^[^ ^191^ ^]^ reported an efficient water remediation by piezo‐pyro‐co‐catalysis based on BST NPs under magnetic stirring and low temperature (LT) or high temperature (HT) variations (**Figure** **48**). The magnetic stirring serves as a mechanical source. As can be seen from Figure 48a, the SBT sample under MS‐LT shows the highest piezo‐pyro‐cocatalytic performance. At the same time, the surfactant‐decorated sample with a reduced mechanical force transfer shows lower degradation performance. These results support the contribution of piezoelectric effect in enhancing the piezo‐pyro‐cocatalysis (Figure 48b). Since most ferroelectrics show *T*_C_ far away from room temperature, in order to develop other material systems, defect engineering and microstructure engineering would be helpful to promote piezo‐pyro‐cocatalytic dye degradation.

##### Photo‐Pyro‐Cocatalytic Dye Degradation

**Figure 49:**
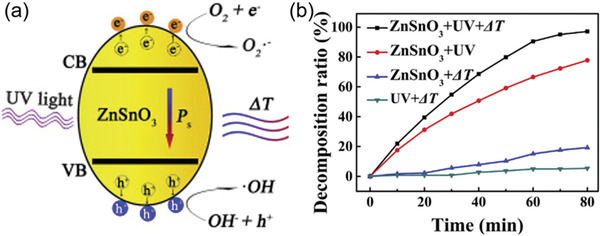
a) Illustrated principle and b) performance of the photo‐pyro‐cocatalytic dye degradation by using ZnSnO_3_ nanoparticles. Reproduced with permission.^[^ ^204^ ^]^ Copyright 2020, Elsevier.

Photo‐pyro‐cocatalytic process not only facilitate the H_2_ generation, but also enhance the dye degradation efficiency. As shown in **Figure** **49**, Chen et al.^[^ ^204^ ^]^ verified the photo‐pyro‐cocatalytic dye degradation of ZnSnO_3_ nanoparticles via sol‐gel method. A high degradation ratio of ≈98.1% was obtained, which is much larger than that of the single case of photocatalysis (≈76.8%) or pyrocatalysis (≈20.2%). These results suggest the application potential of photo‐pyro‐cocatalysis in treatment of wastewater. However, there is still a growth space for photo‐pyro‐cocatalytic performance since both bandgap and *T*_C_ can be tuned by defect engineering. Therefore, the limitation in using ferroelectrics for photo‐pyro‐cocatalysis can be removed, and more works in this field are highly expected in the near future.

##### Photo‐Piezo‐Pyro‐Cocatalytic Dye Degradation

**Figure 50:**
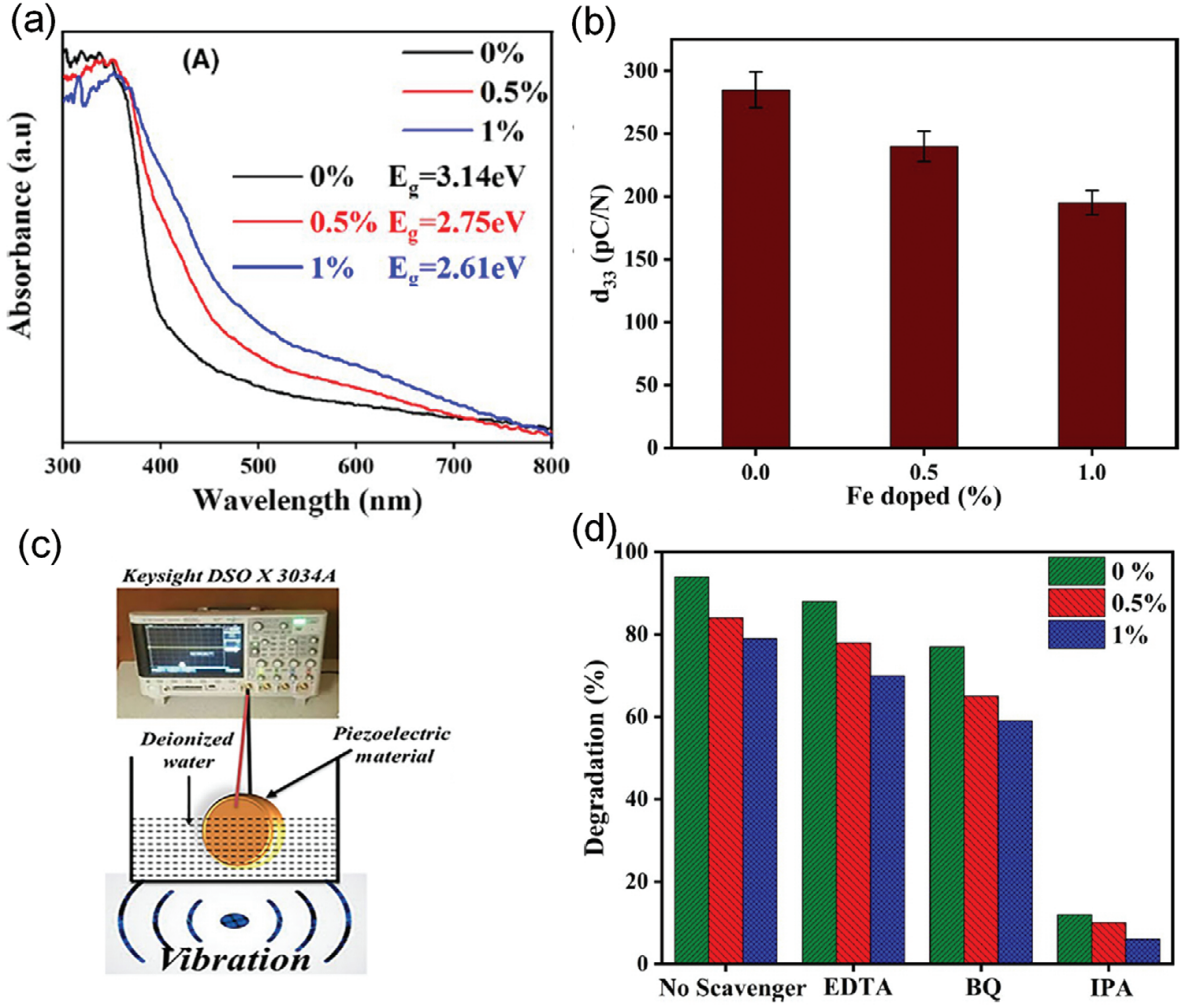
a) Extracted Tauc‐plots for Fe doped Ba_0.85_Ca_0.15_(Ti_0.9_Zr_0.1_)_1‐_ *_x_* Fe*x*O_3_ (*x* = 0, 0.5, and 1%) BCZTO‐Fe with calculated bandgaps indicated. b) Piezoelectric coefficient *d*_33_ as a function of doping level. c) Experimental setup of the photo‐piezo‐pyro‐cocatalysis. d) Comparison of the degradation efficiencies by measuring different scavengers. EDTA: Ethylenediaminetetraacetic acid, BQ: benzoquinone, IPA: isopropyl alcohol. Reproduced with permission.^[^ ^95^ ^]^ Copyright 2020, Wiley.

The combination of photocatalysis, piezocatalysis, and pyrocatalysis will be an interesting topic in the future given that defect engineering can tune the bandgap, piezoelectricity, and *T*_C_. Recently, Sharma and Vaish^[^ ^95^ ^]^ did a pioneer work in this field by using Fe doped Ba_0.85_Ca_0.15_(Ti_0.9_Zr_0.1_)_1‐_ *_x_* Fe*_x_* O_3_ ceramics. It is found that though the doping of Fe in the composition can enlarge the absorption (**Figure** **50**a), the piezoelectricity decreases as doping level increases (Figure 50b). Figure 50c shows the illustrated experimental setup for the experiments. Substantial photo‐piezo‐pyro‐cocatalytic dye degradation can be achieved (Figure 50d). However, the result shows that doping will decrease the catalytic performance. There are many reasons for the low efficiency after doping. First, the experiments use ceramic pellet, which makes it hard to have large surface area for the reaction. Second, the decreased *d*_33_ indicates a large leakage current after doping. To improve the catalytic performance, the host composition needs to be improved since not all system shows leakage after doping as we have reported.^[^ ^90^ ^]^ In addition, nanoscale particles should be better to perform the experiments compared with ceramic pellets.^[^ ^119^ ^]^

**Figure 51:**
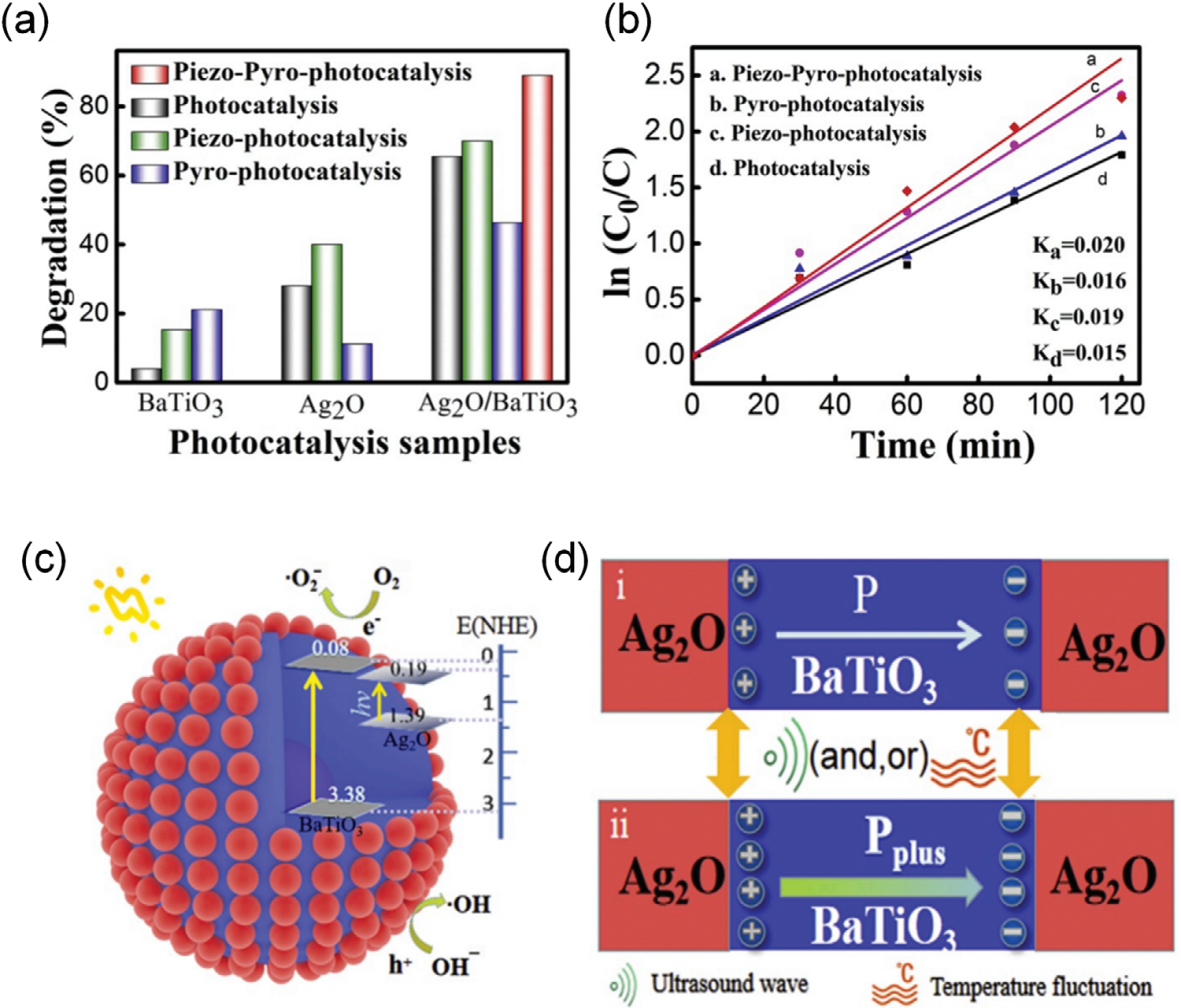
a,b) Photocatalytic dye degradation performance of Ag_2_O/BaTiO_3_ heterostructure microspheres by the piezo‐pyro‐phototronic synergistic effect. c) Schematic diagram of the microsphere, band structure, and working mechanism. d) Change of electric potential under different conditions. i and ii represent the electric potential under light case and light+ultrasound wave or light+temperature fluctuation. Reproduced with permission.^[^ ^203^ ^]^ Copyright 2020, Elsevier.

The enhancement in cocatalyst by harvesting different energy sources has inspired further research into the possibility of multicatalysis. Ag_2_O/BaTiO_3_ heterostructure composite processed by hydrothermal method is used as a representative material to study the photo‐piezo‐pyro‐cocatalytic dye degradation.^[^ ^203^ ^]^ The degradation rate of MO under different conditions is shown in **Figure** **51**. Among them, the coupling effect between semiconductor Ag_2_O, piezoelectricity, and pyroelectricity shows better performance. The highly efficient photoexcitation of Ag_2_O leads to an improved degradation efficiency. The decomposition of MO could be almost 100% finished within 60 min under piezo‐pyro‐photocatalysis. The degradation rate of Ag_2_O/BaTiO_3_ by photo‐piezo‐pyro‐cocatalysis is 0.020 min^−1^, which is nearly 1.3 times that of pyro‐photocatalysis (0.016 min^−1^) and 1.4 times of photocatalysis (0.015 min^−1^). This result makes it promising for photo‐piezo‐pyro‐cocatalysis based on photoferroelectrics.

#### 5.2.2 CO_2_ Reduction

The increase amount of CO_2_ has aroused much concern about global warming. Reducing/minimizing the level of CO_2_ in the atmosphere becomes an urgent need to protect our earth. Carbon capture/storage and carbon utilization are the two main solutions. Compared with carbon capture/storage, the utilization of CO_2_ into chemicals or fuels is promising to provide a sustainable and ecofriendly solution. This can be exampled by converting CO_2_ into formic acid (HCOOH), carbon monoxide (CO), ethylene (C_2_H_4_), ethanol (C_2_H_5_OH), and methane (CH_4_).^[^ ^205^, ^206^, ^207^ ^]^ Therefore, the conversion of CO_2_ into fuels not only forms a chemical recycling of CO_2_ but also creates economic value. CO_2_ reduction can be achieved by chemical reduction and electrochemical approach. The former generally needs higher temperature or higher pressure due to the high stability of CO_2_, which makes it costive and limits its commercialization. The latter approach has gained increasing attention by using catalyst and operating at ambient pressure and temperature.^[^ ^205^, ^206^, ^207^ ^]^ The electrochemical process based on ferroelectric catalyst is simple and can make use of renewable energy sources, such as solar and mechanical vibration.^[^ ^208^ ^]^ Therefore, electrochemical CO_2_ reduction has the potential to provide a sustainable, long‐term approach to reduce/manage atmospheric CO_2_ levels.

##### Piezocatalytic Driven CO_2_ Reduction

**Figure 52:**
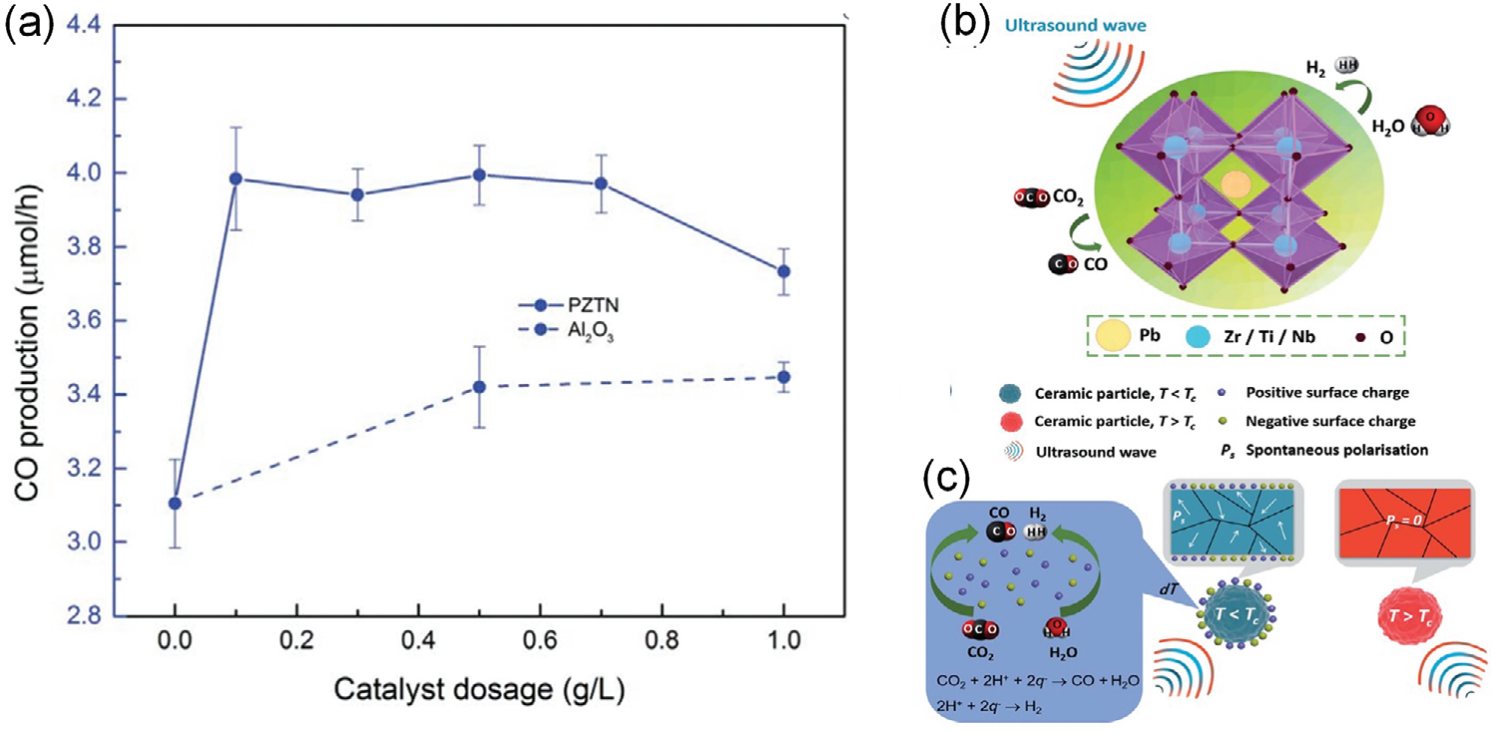
a) CO production by PZTN, the result of nonferroelectric Al_2_O_3_ was provided as a comparison. b) Schematic of mechanism for CO_2_ reduction and H_2_ generation based on the application of ultrasound vibration to ferroelectric PZTN where electrochemical reaction emerges during ultrasonic excitation. c) Enhanced CO production and H_2_ generation via piezo‐catalytic and sonochemical effects. Reproduced with permission.^[^ ^208^ ^]^ Copyright 2021, Royal Chemical Society.

Piezocatalytic CO_2_ reduction can be a by‐product of piezocatalytic water splitting. Zhang et al.^[^ ^208^ ^]^ reported an efficient CO_2_ reduction based on Nb doped Pb_0.99_Zr_0.95_Ti_0.05_Nb_0.02_O_3_ (PZTN) (**Figure** **52**a). The Nb doping can tune the *T*_C_ to close to room temperature,^[^ ^209^ ^]^ which gives rise to high piezoelectricity in order to trigger the reduction of CO_2_.

As shown in Figure 52b, with the application of ultrasound vibration, the change of spontaneous polarization excites charges to the particle surface to initiate redox reactions under periodic ultrasonic acoustic pressure (*σ*). The intrinsic charge carriers including  *q* ^+^ and  *q* ^−^  will transfer to the opposite surfaces of PZTN particles and react with CO_2_ and H^+^ to produce CO and H_2_, respectively. The reaction formulas have been shown as Equations (24), (25), (26).

(24) $$\mathrm{PZTN}\overset{\mathrm{vibration}}{\to }\mathrm{PZT}N_{+}\;q^{+}+q^{-}$$

(25) $$CO_{2}+2H^{+}+2q^{-}\to \mathrm{CO}+H_{2}O$$

(26) $$2H^{+}+2q^{-}\to H_{2}$$

Actually, the absolute potentials for CO_2_ reduction and water splitting are ≈0.38–‐0.61 V and ≈1.23 V, respectively. When the surface charges release, the electric potential can trigger the production of CO and H_2_ due to piezocatalysis in combination with sonochemical processes.

##### Pyrocatalytic CO_2_ Reduction

Pyrocatalytic CO_2_ reduction works upon the pyroelectric effect of ferroelectrics, in which the heat energy can be converted into electric energy via cooling–heating cycles.^[^ ^210^, ^211^, ^212^ ^]^ Since the variations of temperature are recurring phenomena in our environment,^[^ ^213^ ^]^ it would be a sustainable energy source.

**Figure 53:**
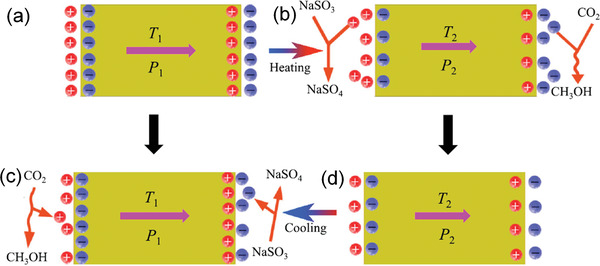
The illustrated mechanism of pyrocatalytic CO_2_ reduction by Bi_2_WO_6_ nanoplate. a) The temperature of catalyst remains constant, its spontaneous polarization in an equilibrium state with the bound charges. b) The rapid rise in temperature broke the balance, and thus induce CO_2_ reduction reaction. c) A new balance is established after the CO_2_ reduction reaction. d) When the temperature drops, the opposite charges transfer and lead to a new CO_2_ reduction process. Reproduced with permission.^[^ ^60^ ^]^ Copyright 2021, Springer Nature.

Xiao et al.^[^ ^60^ ^]^ found that the pyroelectric properties of ferroelectrics could be used for cost‐effective and ecofriendly CO_2_ reduction. For example, layered perovskite bismuth tungstate (Bi_2_WO_6_) nanoplate could harvest heat energy from temperature variation. An methanol yield as high as 55.0 µmol g^‐1^ can be achieved after experiencing 20 cycles of temperature variation (15 °C to 70 °C). The temperature variation should cover the *T*_C_ in order to increase pyroelectric charges and trigger the electrochemical process. As shown in **Figure** **53**, when there is no temperature variation, the internal polarization is balanced by external bound charges. The internal polarization reduces when the temperature increases and thus breaks the balance, which induces free charges. The negative charges will react with CO_2_ to form methanol and the free positive charges would transfer Na_2_SO_3_ to Na_2_SO_4_. In return, the decrease of temperature causes the increase of spontaneous polarization, which again breaks the balance and triggers CO_2_ reduction. Therefore, the cycle of the temperature variation can lead to continuous CO_2_ reduction.^[^ ^60^ ^]^ Since the *T*_C_ of ferroelectrics can be tuned by defect‐doping, more ferroelectrics would be available for use in pyrocatalysis and the research in the field of pyrocatalytic CO_2_ reduction is highly expected in the near future.

##### Piezo‐Pro‐Cocatalytic CO_2_ Reduction

The merit of multifunctionalities in ferroelectric gives the chance for piezo‐pyro‐cocatalytic CO_2_ reduction. When *T* < *T*_C_, the synergistic effect of pyroelectric charge and piezoelectric charge would continuously drive CO_2_ reduction. When *T* > *T*_C_, CO_2_ reduction would be slow since there is only sonochemical effect. The change in spontaneous polarization can drive the separation of surface charge, and the release of surface charge would reach the maximum at *T*_C_. Charge can be created by both ultrasonic vibration and temperature variation. Zhang et al.^[^ ^208^ ^]^ reported a polarization tunable piezocatalytic activity of Nb‐doped PZT with a low *T*_C_ of ≈38 °C and obtained a CO_2_ reduction rate of 789 µmol g^‐1^ h^‐1^, which is much larger than those obtained from just pyrocatalytic effects. Based on defect‐engineered ferroelectrics, piezo‐pyro‐cocatalytic CO_2_ reduction will gain more attention in the future.

## 6 Summary and Perspectives

Conventionally, the concept of ferroelectrics is well known in microelectronics and energy storage, such as capacitive electronics, capacitive energy storage, and ferroelectronics.^[^ ^214^, ^215^, ^216^, ^217^ ^]^ The separation of charge carriers due to spontaneous polarization makes ferroelectrics promising for catalytic dye degradation and H_2_ generation. This is further promoted thanks to the advances in tuning the bandgap and microstructure of ferroelectrics. For example, ferroelectrics can be bandgap engineered to have UV–Vis–NIR broadband absorption, which breaks the mainly UV absorption dilemma of ferroelectrics.^[^ ^90^ ^]^ Therefore, the broadband light photoferroelectrics are promising for many application potentials, including H_2_ generation, environmental multisource energy driven generator, dye degradation, and self‐powered environmental multisource signal sensing.

### 6.1 Environmental Multisource Energy Driven Generator

**Figure 54:**
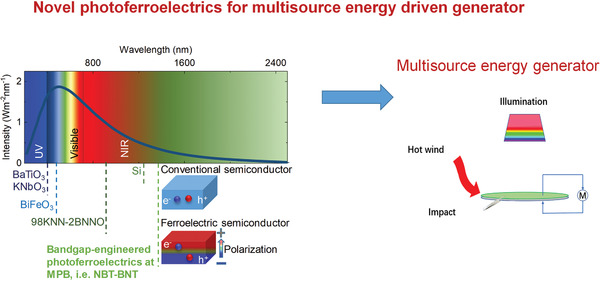
Novel photoferroelectrics for multisource energy driven generator. Left) Reproduced with permission.^[^ ^119^ ^]^ Copyright 2021, Elsevier; Right) Reproduced with permission.^[^ ^90^ ^]^ Copyright 2019, Wiley.

Own to high piezoelectricity, UV–Vis–NIR photovoltaic effect, and pyroelectric effect, novel photoferroelectrics ceramics or films can convert mechanical, solar, and heat into electricity. This kind of multisource energy driven generator can be used to power wearable electronics and intelligent devices, such as self‐driving portable electronics, and multi‐energy harvesters.^[^ ^111^, ^218^, ^219^ ^]^ As shown in **Figure** **54**, multisource energy‐driven nanogenerators (MEDNG) can be designed, which is expected to give higher power and efficiency.^[^ ^90^, ^119^ ^]^ Taking the Ni doped NBT‐BNT ceramic as an example, highly improved photocurrent can be easily induced by combination of solar light illumination, mechanical force, and heat.^[^ ^90^ ^]^ An interesting thing is that the behavior of each environmental energy source or signal is significantly different, which may provide potential to detect environmental signals according to their unique characteristic response. The advances in developing flexible PENG^[^ ^29^, ^109^, ^171^, ^220^, ^221^, ^222^, ^223^, ^224^ ^]^ can also be used to similarly develop flexible MEDNG. For example, by depositing photoferroelectric thin films on textile substrates, superflexible MEDNG can be designable.

### 6.2 Environmental Multisource Energy Driven Catalysis

**Figure 55:**
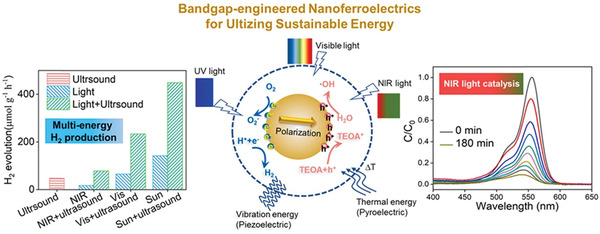
UV–Vis–NIR light responsive nano‐ferroelectric for efficient photo‐piezocatalytic water splitting and pollutant degradation. The left is catalytic performance, the middle is the mechanism, the right is the adsorption spectra of RhB solution mediated by NBT‐BNT NPs@Ag under NIR light. Reproduced with permission.^[^ ^119^ ^]^ Copyright 2021, Elsevier.

As shown in **Figure** **55**, by optimizing the properties of photoferroelectrics based on defect engineering or microstructure engineering, ferroelectrics with improved light absorption, high piezoelectricity, and high pyroelectricity can be designed for co‐catalysis.^[^ ^119^, ^208^, ^225^ ^]^ Photoferroelectrics can even show novel/outstanding NIR light catalytic performance in comparison with that of the conventional catalyst that generally absorbs light in the UV–vis range.^[^ ^119^ ^]^ Actually, different photoferroelectrics show different level of photocurrent *J*_sc_, from nA cm^‐2^ level in KNBNNO^[^ ^35^, ^92^ ^]^ to µA cm^‐2^ level in PbTiO_3_–Bi(Ni_1/2_Ti_1/2_)O_3_.^[^ ^134^ ^]^ Therefore, it is necessary to explore new photoferroelectrics with high photoelectric conversion efficiency.

**Figure 56:**
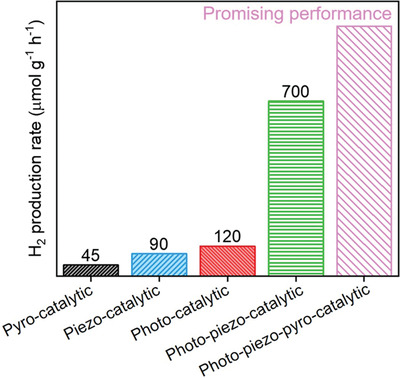
Illustration of the H_2_ generation single and multicatalytic conditions extracted from refs. [68, 87, 151, 172]. The much higher performance of photo‐piezo‐pyro‐cocatalytic behavior is highly expected.

From **Figure** **56**, it is obvious that the photo‐piezocatalytic behavior is not just supposition of photocatalysis and photocatalysis, it has an effect of 1+1>1 owing to their synergistic effect. Therefore, a promising performance of photo‐piezo‐pyro‐catalytic effect is highly expected in the near future.

### 6.3 Pyro‐Photoelectric Catalytic H_2_ Generation

**Figure 57:**
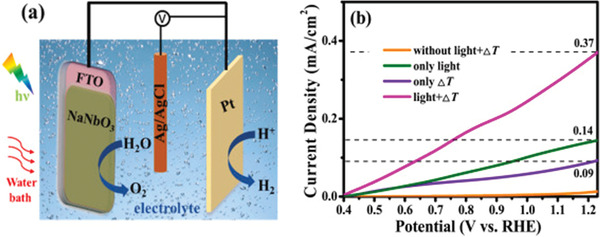
a) Working principle and b) current density of pyro‐photo‐electric catalysis using NaNbO_3_ under solar, cold‐hot cycles. Reproduced with permission.^[^ ^226^ ^]^ Copyright 2021, Elsevier.

Recently, Zhang et al.^[^ ^226^ ^]^ even reported a novel concept of catalytic process named “pyro‐photo‐electric catalysis” based on coupling of pyroelectric catalysis (PREC) and photoelectrochemical (PEC) catalysis by using NaNbO_3_ nanocubes. As shown in **Figure** **57**, high photocurrent density of ≈0.37 mA cm^‐2^ can be achieved at 1.23 V versus a reversible hydrogen electrode (RHE) under 20–50 °C heating–cooling cycles + light illumination. The photocurrent density is nearly 3.1 times larger than that of the pyrocatalytic case and 1.6 times larger than that of the photoelectrochemical case, respectively. The significantly higher catalytic efficiency arises from the increased carrier concentration, including photogenerated carriers and pyroelectric carriers, and pyroelectric potential assisted charge separation.

### 6.4 Self‐Powered Multisource Environmental Signal Sensing

Wearable electronics developed quickly from human‐motion sensors^[^ ^227^, ^228^, ^229^, ^230^, ^231^, ^232^ ^]^ to biomedical field^[^ ^233^, ^234^, ^235^ ^]^ and intelligent bionic robot industry.^[^ ^236^, ^237^, ^238^ ^]^ The fast development have greatly changed our life. Various kinds of sensors are required to be implantable or wearable for our body, which need to be powered by rechargeable batteries. In order to tackle the challenge of power supply and reduce the potential harm of the implantation or battery to our body, self‐powered sensing have aroused tremendous interest in recent years.^[^ ^239^, ^240^, ^241^, ^242^ ^]^ Among those solutions for power supply, PENG^[^ ^243^, ^244^, ^245^ ^]^ and triboelectric nanogenerators based sensors^[^ ^246^, ^247^, ^248^ ^]^ have been widely investigated.

**Figure 58:**
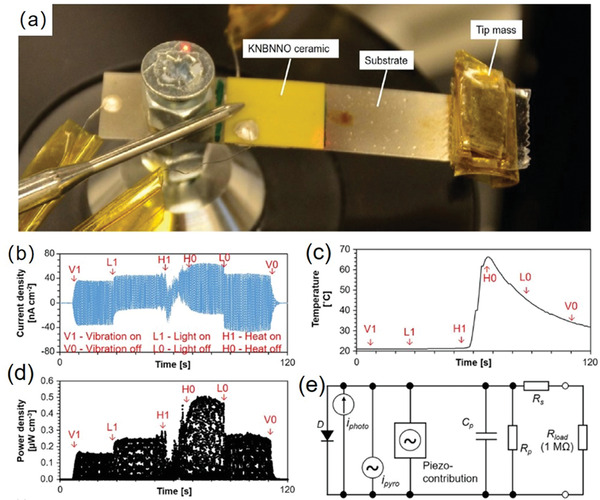
a) Picture of the fabricated energy harvesting‐sensing device (KNBNNO cantilever). b) Output current density and c) output power density of the KNBNNO cantilever with vibration, light, and heat excitations. d) Temperature profile of the measurement shown in (b,c). d) Equivalent circuit of the piezoelectric, photovoltaic, and pyroelectric effects. Reproduced with permission.^[^ ^252^ ^]^ Copyright 2020, Wiley.

However, compared with above‐mentioned single‐source energy harvesters (i.e.,PENG, TENG),^[^ ^249^, ^250^, ^251^ ^]^ photoferroelectric based multisource energy harvesting may drive conceptually new device design.^[^ ^252^ ^]^ For example, Bai et al.^[^ ^252^ ^]^ recently reported a demonstration of concept for an integrated multisource energy harvesting‐sensing system by setting a KNBNNO cantilever as an example (**Figure** **58**). The emerging multisource energy harvesting performance of photoferroelectrics is promising to improve the output performance of ferroelectric‐based sensors.^[^ ^253^, ^254^, ^255^ ^]^

As illustrated in Figure 58, the unique characteristic responses of photoferroelectrics to light, mechanical force, and heat variation make it promising for designing multisource energy harvesting‐sensing devices or self‐powered multisource environmental sensing.^[^ ^91^, ^93^ ^]^ Moreover, since photovoltaic effect can induce photostriction by inverse piezoelectric effect, the novel photoferroelectrics is promising to be used in light‐driven actuators in self‐powered devices or all‐optical switching with spintronics.^[^ ^256^, ^257^, ^258^ ^]^

### 6.5 Challenges and Chances

As a whole, with defect/microstructure engineering and novel preparation methods, ferroelectrics with enhanced photo‐absorption, piezoelectricity, and pyroelectricity are promising materials for emerging applications in energy and environmental pollution. However, there are still challenges. First, though the absorption window of photoferroelectrics has been largely enlarged from UV to visible/NIR range, their photocurrents are still low, compared with conventional semiconductors. For example, the photocurrent of commercial semiconductor‐based (i.e., Si) solar cell can easily reach mA cm^‐2^ level. Whereas the UV–Vis–NIR light responsive photoferroelectrics, such as NBT‐BNT, are currently still in 100 nA cm^‐2^ level. The low photocurrent will initially limit their efficiency in photoelectric conversion. One direct way to intrinsically increase the photocurrent is to further modify and optimize the band structure, such as introducing more gap states. Another intrinsic way is to explore ferroelectrics with high electron mobility in order to improve the charge separation. To extrinsically increase the photocurrent, transparent MPB ferroelectrics or thin films would be better, the former decreases the light scattering, while the latter would be favorable to reach an appropriate thickness to match the depletion region. Second, to measure and harvest the multisource energies, both the measurement system and multisource energy harvesting devices need to be either modified or designed. For example, the multisource energy harvesting‐sensing system still needs to solve technical issues for a more practical and further development as suggested by Bai et al.^[^ ^252^ ^]^ To better provide a multisource energy harvesting‐sensing system, multidisciplinary collaboration from material scientists to electronic and electrical engineers would be expected so as to complete device design, material preparation, and device fabrication. Finally, the defect‐engineered ferroelectrics to be used in catalysis need to be in nanosize in order to increase specific surface area. Therefore, new methods such as SHS method need to be modified and explored in order to improve the introducing of doping elements in the matrix.

In addition to the above potential applications, recently, ferroelectric‐based catalytic desulfurization,^[^ ^117^ ^]^ and organic synthesis^[^ ^259^, ^260^, ^261^ ^]^ are also gaining increasing attention, which are also could be important research directions. In a more broadened view, defect/microstructure engineering may also show promising application in modifying the properties of perovskite materials in addition to ferroelectric perovskite oxides, such as 2D transition metal dichalcogenides, back phosphorus, and so on.^[^ ^262^ ^]^

## Conflict of Interest

The authors declare no conflict of interest.
